# Computational Intelligence in Stochastic Reconstruction of Porous Microstructures for Image-Based Poro/Micro-Mechanical Modeling

**DOI:** 10.1007/s11831-025-10313-9

**Published:** 2025-08-26

**Authors:** Jinlong Fu, Wei Tan, Dunhui Xiao, Xiaoying Zhuang

**Affiliations:** 1 Department of Engineering Mechanics, School of Mechanics and Aerospace Engineering, Dalian University of Technology, Dalian, 116023 China; 2 School of Engineering and Materials Science, Faculty of Science and Engineering, Queen Mary University of London, London, E1 4NS UK; 3 School of Mathematical Sciences, Key Laboratory of Intelligent Computing and Applications (Ministry of Education), Tongji University, Shanghai, 200092 China; 4 Institute of Photonics, Faculty of Mathematics and Physics, Leibniz University Hannover, Hannover, 30167 Germany

## Abstract

Understanding microstructure-property relationships (MPRs) in random porous media is a fundamental challenge across numerous scientific and engineering disciplines. Image-based poro/micro-mechanical modeling offers a powerful noninvasive technique to investigate MPRs via numerical simulations. However, the stochastic nature and inherent randomness of porous media necessitate extensive datasets of 3D digital microstructures for reliable statistical analysis. Stochastic microstructure reconstruction provides an efficient and cost-effective approach to generate large numbers of virtual microstructures using limited statistical information from real porous materials, establishing it as a critical tool for advancing research in this field. This review presents a comprehensive examination of stochastic reconstruction methodologies, spanning traditional algorithm-based methods and emerging computational intelligence-based approaches. Particular emphasis is placed on computational intelligence-based approaches, such as generative adversarial networks, while also discussing the foundational contributions and limitations of traditional methods. These advancements have significantly enhanced the fidelity, efficiency and scalability of microstructure reconstruction, enabling robust statistical investigations of MPRs, such as Monte Carlo analysis. Despite substantial progress, challenges such as data scarcity, high computational costs, limited interpretability, and the need for physically realistic reconstruction remain, especially for complex pore network systems. Emerging trends, including physics-aware machine learning and hybrid AI frameworks integrating domain-specific knowledge, offer promising avenues to overcome these limitations. By bridging disciplinary gaps, this review provides a roadmap for future research in stochastic microstructure reconstruction, facilitating deeper insights into MPRs and broadening applications across various scientific and engineering domains.

## Introduction

Random porous media are ubiquitous across natural environments, engineering applications, and scientific disciplines. Notable examples include rocks [1], soils [2], concretes [3], lightweight materials [4], membranes [5], porous electrodes [6], heterogeneous catalysts [7], porous scaffolds [8], and biological tissues [9]. The microstructural characteristics of porous media are widely recognized as fundamental determinants of their macroscopic physical properties [10–12]. For instance, transport phenomena such as fluid permeation, mass diffusion, electrical conduction, and heat transfer within porous media are strongly influenced by microstructural features. These correlations dictate key transport properties, including permeability [13, 14], effective diffusivity [15], formation resistivity factor [16], and thermal conductivity [17]. Beyond transport properties, microstructural characteristics significantly impact the behaviors, performance and functionality of porous materials across diverse domains. For example, the internal microstructure of lightweight materials [18] plays a crucial role in determining their mechanical properties, such as impact resistance. In cementitious materials, pore structure governs chemical interactions with the environment, influencing structural degradation and durability [19]. For porous rocks and concretes, compressive and tensile strengths are highly sensitive to pore morphology and architecture [20]. In solid oxide fuel cells, the microstructure of electrodes critically affects energy storage performance and conversion efficiency [21]. For heterogeneous catalysts, pore architecture directly determines reaction efficiency by controlling reactant transport and catalytic activity [22]. In biological systems, the hierarchical structure of porous scaffolds and bone tissues plays a vital role in processes such as cell migration, nutrient diffusion, waste removal, and bone remodeling [23, 24]. These examples underscore the pivotal role of microstructural characteristics in determining the macroscopic behavior and functionality of porous media in both natural and engineered systems.

Understanding the *microstructure-property relationships* (MPRs) of random porous media remains one of the most fundamental questions across numerous research disciplines [10–12], including geoscience, civil engineering, materials science, mineral engineering, energy storage, chemical engineering, and biomedical engineering. These MPRs are invaluable for modeling and predicting the macroscopic physical properties of porous media directly from measurable microstructural features, without relying on expensive and time-consuming experiments, which are critical for optimizing the performance and functionality of porous materials. Moreover, efficient and reliable predictions of the time-dependent properties of porous media are crucial for reducing uncertainties in engineering applications [25, 26]. By accurately capturing the evolution of these properties over time [27, 28], such predictions enable better design, optimization, and risk management across a wide range of industries. However, the inherent randomness and stochastic nature of porous media pose substantial challenges to precise microstructural analysis and accurate MPR modeling. These challenges remain unresolved due to limitations in experimental techniques and analytical theories [29–33]. Accurate characterization and a deep understanding of porous media are essential to bridge the gap between microstructural features and macroscopic physical properties [34–37].

### Image-Based Poro/Micro-Mechanical Modeling

**Fig. 1:**
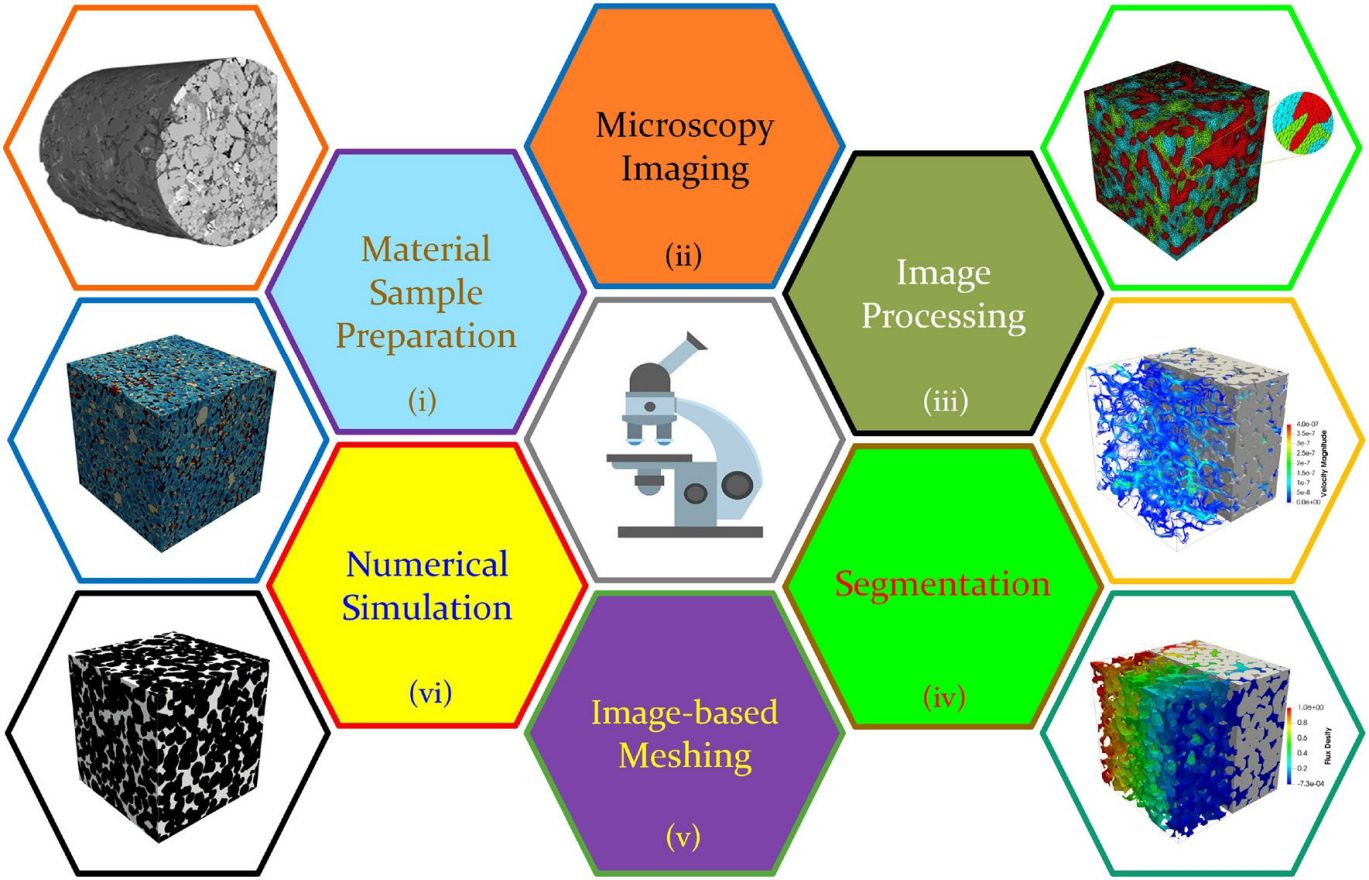
The workflow of image-based poro/micro-mechanical modeling for porous media typically involves six key steps: (i) Sample preparation, (ii) Microscopy imaging, (iii) Image processing, (iv) Segmentation, (v) Meshing, and (vi) Numerical simulation

In recent decades, significant progress has been made in digital microscopic imaging and computer-aided numerical simulation. Image-based poro/micro-mechanical modeling [33, 37–40], as illustrated in Fig 1, offers a versatile, non-destructive, and non-invasive approach for investigating the MRPs in various porous media. Modern microscopy imaging techniques have been able to digitize the geometry of opaque porous media, usually called microstructures, into 2D or 3D images at different resolution levels and various length scales [36, 37, 41]. Optical microscopy [36], scanning electron microscopy (SEM) [42], backscattered electron imaging (BSE) [43], atomic force microscopy (AFM) [44] and transmission electron microscopy (TEM) [45] are commonly used techniques to provide 2D visualizations of porous microstructures. High-quality 3D digital microstructures can be acquired through X-ray micro-computed tomography (micro-CT) [46, 47], nuclear magnetic resonance (NMR) imaging [41], and focused ion beam scanning electron microscopy (FIB-SEM) [48]. Although the imaging mechanisms of various microscopy techniques are different, the acquired digital images at the same resolution level are comparable [49]. Digital microstructures can be used for a variety of prediction, simulation, design and diagnostic purposes, which radically boosts microstructural analyses in porous media research [33, 37–40].

**Fig. 2:**
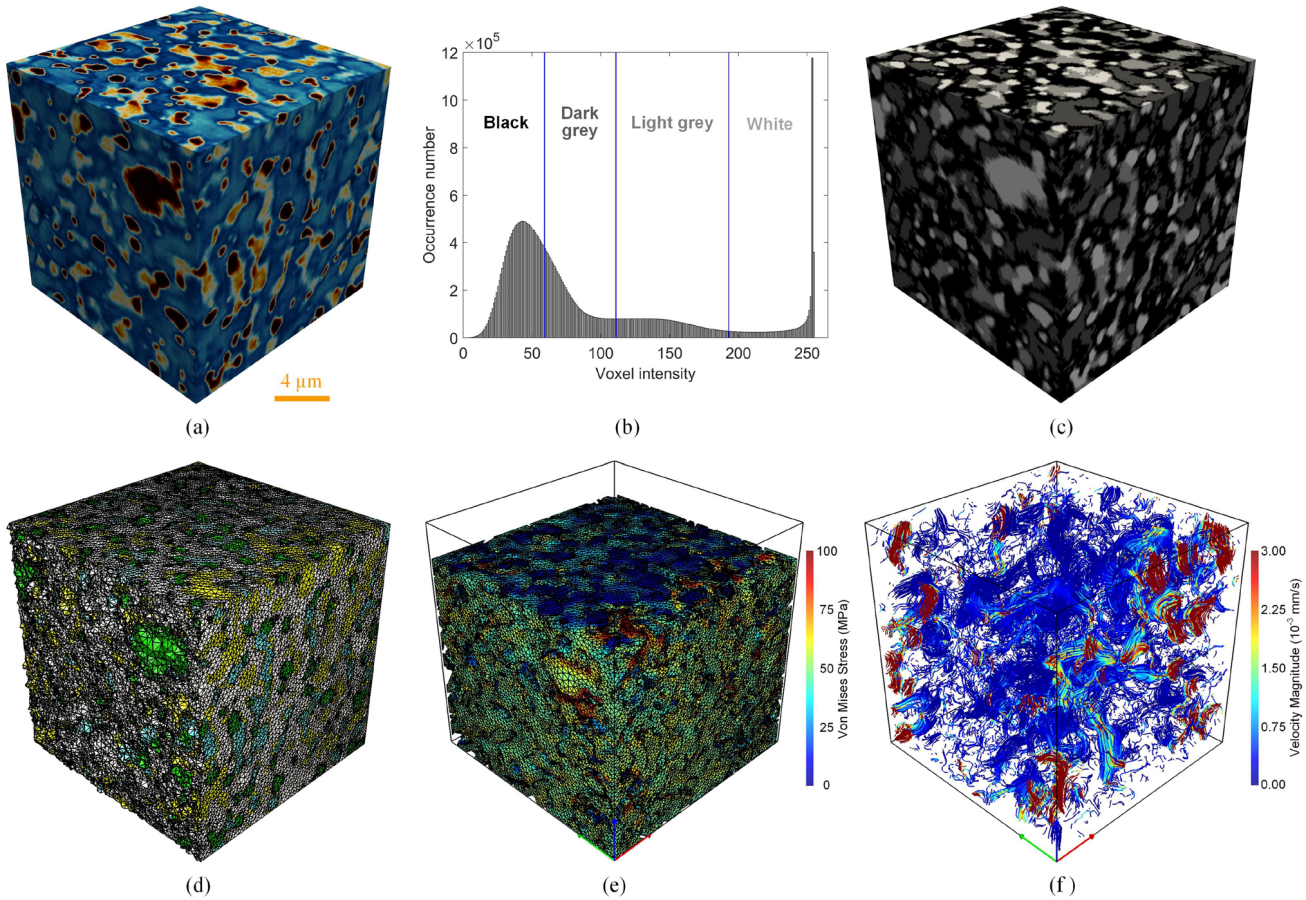
Graphical illustration of the image-based poro/micro-mechanical modeling workflow for random porous media: (**a**) SEM-BEI imaging of a multiphase material with sub-micron resolution; (**b**) The histogram of voxel intensities used for phase thresholding; (**c**) The segmented digital microstructure showing four distinct material phases, obtained through a histogram-based segmentation method in *ImageJ*; (**d**) The unstructured mesh generated using *Gmsh* while preserving the complicated pore-solid geometry; (**e**) Micro-mechanical modeling of solid deformation under axial compression using FEM in *Abaqus*; and (**f**) Pore-scale simulation of fluid flow through the porous domain under an applied pressure gradient using FEM in *Simpleware*

As illustrated in Fig 2, high-quality digital microstructures provide high-fidelity geometric models for numerical simulations of various physical processes occurring at the pore/micro scale, enabling to evaluate effective macroscopic properties and explore critical physical phenomena [10, 31, 39, 50, 51]. Image-based meshing techniques [52–54] are able to discretize digital microstructures into computational elements while preserving the pore geometry. Governing partial differential equations can then be solved or approximated on the computational mesh using numerical techniques such as the Finite element method (FEM) [55, 56], Lattice Boltzmann method (LBM) [14, 57], Finite difference method (FDM) [58], Finite volume method (FVM) [59], Discrete element method (DEM) [60, 61], and Phase field method (PFM) [62, 63]. Compared to experimental measurements, image-based poro/micro-mechanical modeling offers much greater flexibility. They allow systematic variations in pore geometries, material properties, applied forces, operating conditions, and boundary conditions, providing insights into their influence on physical behaviors and macroscopic responses [64, 65].

Furthermore, the link between microstructure and macroscopic behavior can be rigorously established through multiscale analysis [66, 67] and mathematical homogenization techniques [68, 69]. These methods provide a formal framework for deriving effective material properties from pore-scale characteristics by assuming scale separation between the micro and macro domains. For instance, asymptotic homogenization enables the derivation of constitutive laws, such as Darcy’s law for fluid flow [70] or Hooke’s law for elasticity [71], by solving local cell problems over a representative microstructural domain. Similarly, numerical multi-scale methods [72, 73], such as FE$^2$ [74] or concurrent schemes [75], allow the coupling of microscale simulations with macroscale behavior in complex, non-periodic microstructures. These approaches are crucial for predicting macroscopic responses such as permeability, effective stiffness, and transport coefficients based on morphological and topological features of material microstructures.

### Stochastic Microstructure Reconstruction

Due to the stochasticity and heterogeneity inherent in pore microstructures, the macroscopic physical properties of porous media (such as permeability, effective diffusivity, electrical conductance, thermal conductivity, and mechanical strength) can fluctuate significantly and exhibit strong uncertainty [10, 11, 31]. Investigating the MPRs of random porous media is fundamentally a statistical learning problem that requires a *complete computational dataset*, which extends far beyond a single representative sample [34, 76, 77]. While advanced microscopy imaging techniques have enabled unprecedented insights into microstructural features, they are often associated with high costs, restricted accessibility, and technical limitations, especially when dealing with opaque and fragile materials [36, 37]. Furthermore, the requirement for extensive datasets of 3D digital microstructures to support reliable statistical analyzes [78–80] further compounds these limitations, particularly in the context of Monte Carlo analysis.

To address this issue, there has been growing interest in leveraging computational methods to generate virtual microstructure samples using the limited geometry and morphology information obtained from measurements and experimental observations [81, 82]. *Stochastic microstructure reconstruction* [34, 83–87] utilizes limited statistical descriptions of real microstructures to synthesize virtual samples while preserving statistical equivalence between them. Compared to microscopy imaging techniques, stochastic reconstruction methods offer a more efficient and economical approach to generating large datasets of microstructure samples. These synthesized samples can form a pseudo-complete computational dataset, enabling comprehensive statistical analyses of MPRs.

For many porous materials, only 2D cross-sectional images of internal microstructures can be acquired from the state-of-the-art microscopic imaging techniques [88]. This limitation is primarily due to several technical and physical barriers: First, the ability to accurately capture the 3D spatial morphology of representative volume elements (RVEs) in highly heterogeneous porous media remains limited, not only due to technological constraints, but also because of fundamental physical interactions between imaging radiation (e.g., X-rays or electrons) and complex sample materials, which can restrict resolution, contrast, and depth penetration [77, 89]; Second, the microstructures of organic materials, such as polymer nanocomposites, are susceptible to alteration or damage caused by radiation, sputtering, heating, or charging during volumetric imaging processes [90]; Third, preparing certain materials, such as soft or delicate specimens, for 3D serial sectioning can be extremely challenging [91]. In contrast, acquiring high-quality 2D cross-sectional images of representative area elements (RAEs) using surface imaging devices is significantly more cost-effective and technically feasible.

**Fig. 3:**
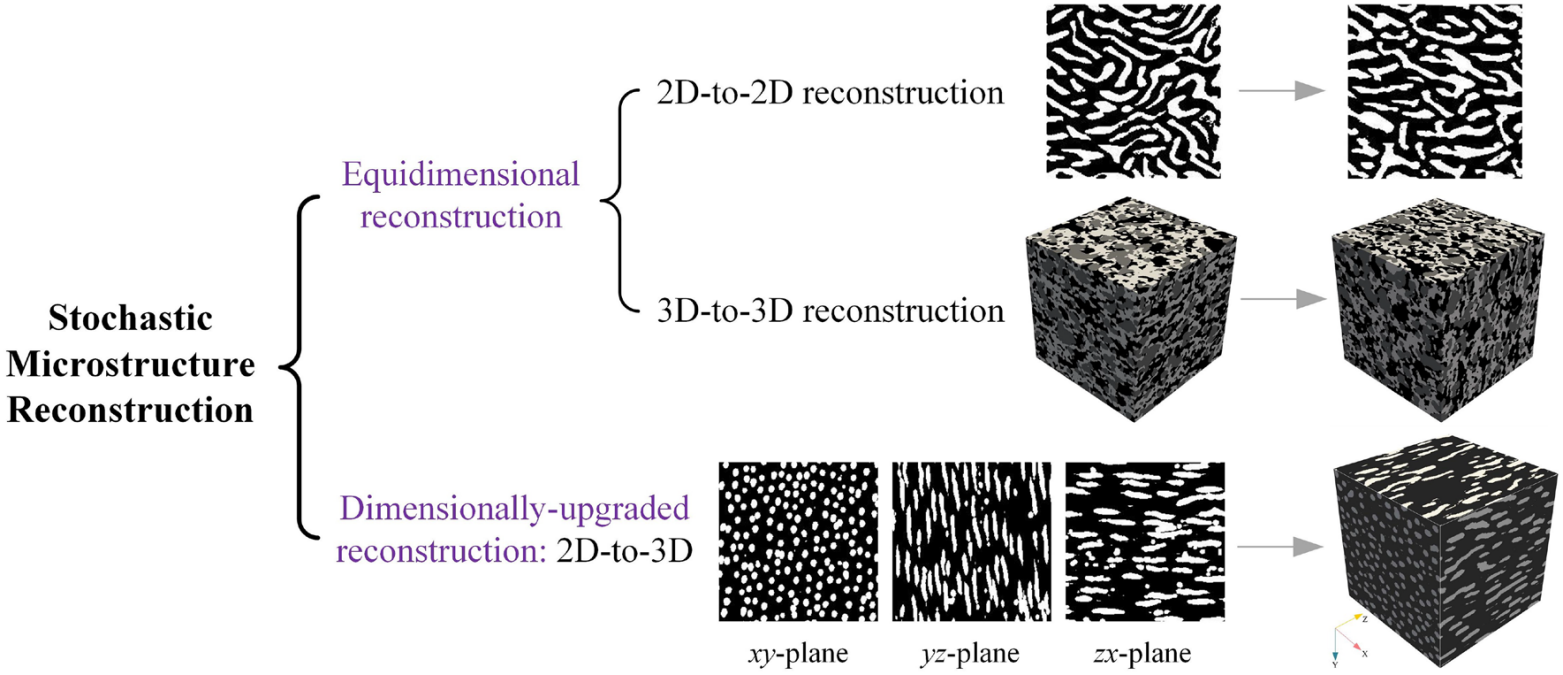
The categories of stochastic microstructure reconstruction for random porous media: (i) Equidimentional reconstruction: 2D-to-2D, and 3D-to-3D; and (ii) Dimensionally-upgraded reconstruction: 2D-to-3D

However, 3D poro/micro-mechanical modeling is often indispensable for gaining a deeper understanding of the physical phenomena occurring within porous media [92, 93]. This necessity arises because significant differences exist between 2D thin sections and the original 3D architectures, particularly in terms of percolation, permeability, and pore network connectivity [77, 94]. To address these discrepancies, stochastic reconstruction methods capable of inferring and synthesizing equivalent 3D microstructures from available 2D slices hold great practical value [77, 91, 95, 96]. In this work, stochastic reconstruction of random porous microstructures is broadly categorized into two primary types: *Equidimensional reconstruction* and *Dimensionally-upgraded reconstruction*, as illustrated in Fig 3. In general, stochastic microstructure reconstruction provides a cost-effective alternative to expensive microscopic imaging techniques. This approach enables the efficient generation of an extensive dataset of virtual microstructure samples, facilitating the large-scale investigation of MPRs.

### Scope, Objectives and Arrangement

Over the past few decades, a diverse range of computational algorithms has been developed and applied to stochastic microstructure reconstruction for random porous media. These include traditional methods such as stochastic optimization-based reconstruction [84], random field transformation [83], sphere packing [97], sequential indicator simulation [98], multiple-point statistics [99], and texture synthesis [76]; as well as more recent machine/deep learning-based approaches like support vector machines [100], decision trees [101], deep belief networks [102], feed-forward neural networks [77], generative adversarial networks (GANs) [103], convolutional neural networks (CNNs) [104], transfer learning [105], and recurrent neural networks [106]. In this review, microstructure reconstruction methods are broadly classified into two main categories: *Traditional algorithm-based reconstruction* and *Computational intelligence-based reconstruction*, based on the computational approach utilized for microstructural characterization and synthesis.

- **Traditional algorithm-based reconstruction**: Explicitly characterizes real microstructures by extracting the key statistical features of interest, and then generates virtual microstructure samples that are statistically equivalent or mathematically consistent to the reals.
- **Computational intelligence-based reconstruction**: Usually extracts and learns morphological features from real microstructures in an uninterpretable or implicit manner, enabling the reproduction of detail-similar and morphologically realistic microstructure samples.

Given the technical complexity and interdisciplinary nature of stochastic microstructure reconstruction, this review aims to deliver a thorough and nuanced overview of the field, encompassing traditional algorithms and modern computational intelligence techniques. Traditional algorithm-based approaches often allow direct control over physically interpretable microstructural features, such as grain size distributions, fiber orientations, pore shapes, pore connectivity, or specific surface areas, through explicit statistical descriptors or input parameters. In contrast, many deep learning-based methods (e.g., GANs or CNNs) tend to learn such microstructural features implicitly, which may limit direct tunability without further conditioning mechanisms. This distinction is critical for practical applications where intuitive and interpretable control over microstructural features is required for inverse design or materials optimization.

Stochastic microstructure reconstruction lies at the intersection of materials science, computational mechanics, and artificial intelligence (AI), requiring innovative approaches to address its inherent challenges. This review emphasizes the growing significance of machine/deep learning-based reconstruction methods, which have revolutionized the field by enabling the synthesis of highly detailed, statistically equivalent, and morphologically realistic microstructures. The review seeks to summarize the existing advancements, highlighting the evolution of methodologies from early statistical models and stochastic optimization techniques to state-of-the-art neural networks such as generative adversarial networks and variational autoencoders. It highlights significant progress, particularly the transformative impact of these advanced methods on characterization accuracy, reconstruction fidelity, computational efficiency, and large dataset generation for reliable investigation of MPRs. It also identifies the key challenges (such as data scarcity and interpretability issues), and the need for physically realistic reconstruction of random microstructures.

The remainder of this review is organized as follows: Section 2 introduces traditional algorithm-based microstructure reconstruction methods, providing brief recaps of several representative approaches. Section 3 systematically categorizes emerging computational intelligence-based reconstruction methods, drawing from a comprehensive literature survey. Section 4 outlines key metrics and methodologies for validating reconstructed microstructure samples. Section 5 presents an in-depth discussion on evaluation criteria of effective reconstruction methods. Finally, Section 6 explores future research directions and concluding remarks on computational intelligence-based microstructure reconstruction.

## Traditional Algorithm-Based Microstructure Reconstruction

The internal microstructures of random porous media usually exhibit remarkable complexity and pronounced stochasticity [10, 31, 107], encompassing variations in pore size, shape, connectivity and component distribution. Due to this inherent randomness, it is virtually impossible to find two natural porous media samples with identical microstructural characteristics in every detail. This complexity underscores the significance of the concept of *statistically equivalent* microstructures, which has become a cornerstone of stochastic microstructure reconstruction [11, 34]. Statistically equivalent microstructures replicate the key statistical and morphological features of real samples without requiring exact replication of every detail, providing a practical and scalable framework for analyzing and simulating the physical behaviors of porous media.

Over the past few decades, a diverse array of computational algorithms has been integrated into stochastic microstructure reconstruction, and representative methods are summarized in Table 1. Among these methods, stochastic optimization-based reconstruction (SOR), random field transformation (RFT), process-based reconstruction (PR), random set-based reconstruction (RSR), and spinodoid topology generation (STG) have emerged as the most widely applied approaches. Traditional algorithm-based reconstruction methods have historically played a pivotal role in synthesizing virtual microstructure samples that capture the statistical essence of real materials. In this section, representatives of traditional reconstruction methods are revisited (including SOR, RFT, PR, RSR and STG), aiming to establish a comprehensive understanding of their principles and contributions. This exploration not only highlights their strengths and limitations but also sets the stage for delving into advanced computational intelligence-based approaches, demonstrating the evolution and growing sophistication of stochastic microstructure reconstruction methodologies.

**Table 1:** Traditional algorithm-based methods for stochastic microstructure reconstruction

Stochastic reconstruction method	Computational algorithm	Reconstruction category	References
Stochastic optimization-based reconstruction (SOR)	Simulated annealing; Genetic algorithms; Particle-swarm algorithm; Tabu search	Equidimensional; Dimensionally-upgraded	[84, 108–112].
Random field transformation (RFT)	Truncation of Gaussian random field; Poisson field transformation; Nonlinear Gaussian field transformation	Equidimensional; Dimensionally-upgraded	[83, 95, 113–116].
Process-based reconstruction (PR)	Random sphere packing; Forming process simulation	Dimensionally-upgraded	[97, 117–120].
Random set-based reconstruction (RSR)	Boolean operations of random sets	Equidimensional	[121–124].
Multiple-point statistics method	Search tree and sequential sampling; Markov chain Monte Carlo simulation	Equidimensional; Dimensionally-upgraded	[89, 99, 125, 126].
Texture synthesis method	Markov random field texture modeling; Texture optimization	Equidimensional; Dimensionally-upgraded	[76, 127, 128].
Phase recovery reconstruction	Fast Fourier transformation	Equidimensional; Dimensionally-upgraded	[129–131].
Patch-based reconstruction	Cross-correlation function	Dimensionally-upgraded	[132, 133].
Random generation-growth method	Cluster growth theory	Equidimensional; Dimensionally-upgraded	[134–136].
Sequential indicator simulation	Sequential indicator simulation	Dimensionally-upgraded	[98, 137].
Maximum entropy method	Markov random field modeling	Equidimensional	[138–140].
Grain packing-based reconstruction	Random grain packing algorithm	Equidimensional; Dimensionally-upgraded	[141–144].
Spinodoid topology generation (STG)	Anisotropic spinodal decomposition	−	[71, 145–148].

### General Workflow

**Fig. 4:**
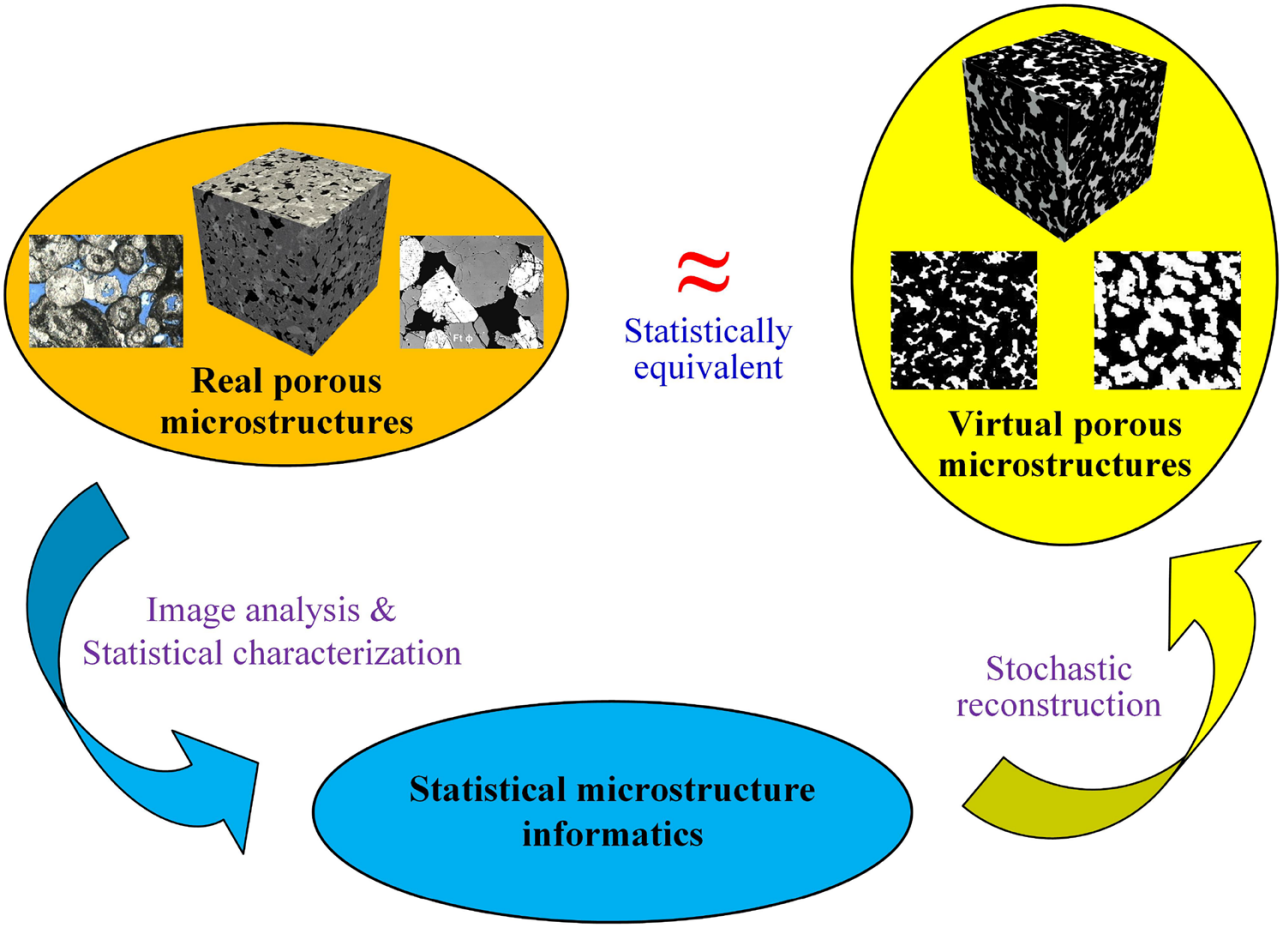
The general workflow of traditional microstructure reconstruction methods

The general workflow of traditional algorithm-based reconstruction methods for random porous microstructures is graphically illustrated in Fig 4, which typically consists of two main steps:

- **Statistical characterization** is to quantify and extract key statistical information from real microstructures through image analysis. Emphasis is often placed on morphological statistics that significantly influence the physical properties of interest, ensuring that microstructural characterization remains relevant to the target application while reducing unnecessary complexity.
- **Stochastic reconstruction** is to generate virtual microstructure samples using the extracted morphological statistics as reconstruction objectives. Rather than replicating real microstructures in exact detail, the reconstruction process aims to maintain statistical equivalence with the target features, effectively capturing the inherent randomness and complexity of porous media.

Such a systematic framework ensures that the reconstructed virtual microstructure samples are statistically faithful representations of real samples, providing a solid foundation for advanced analysis and modeling of MPRs in random porous media. Traditional methods share a common pathway but differ in the computational algorithms employed to statistically characterize and synthesize microstructures. By prioritizing statistical equivalence over exact replication, traditional methods strike a balance between realistic representation and computational efficiency. This focus enables the capture of critical microstructural features that drive material properties/behaviors while minimizing unnecessary complexity.

### Stochastic Optimization-Based Reconstruction

Stochastic optimization-based reconstruction (SOR) was first introduced by Yeong and Torquato in 1998 [84]. The core concept of SOR is to generate virtual microstructures via a stochastic optimization process, where the statistical information extracted from real microstructures is defined as the optimization objective(s) [84, 108, 149, 150]. To achieve this, an energy function $\mathcal {E}$, is formulated to quantify the statistical discrepancy between the trial realization and the real microstructures, given by:

1 $${\mathcal{E}} = \sum\limits_{{i = 1}}^{n} {\omega _{i} } \left\| {D_{i}^{{({\mathrm{trail}})}} - D_{i}^{{({\mathrm{real}})}} } \right\|_{2},$$

where $D_i^{(\text {trial})}$ and $D_i^{(\text {real})}$ represent the *i*th statistical descriptors extracted from the trial realization and the real microstructure, respectively; *n* denotes the total number of used descriptors; and the weight coefficient $\omega _i$ determines the relative importance of each descriptor in the optimization process.

**Fig. 5:**
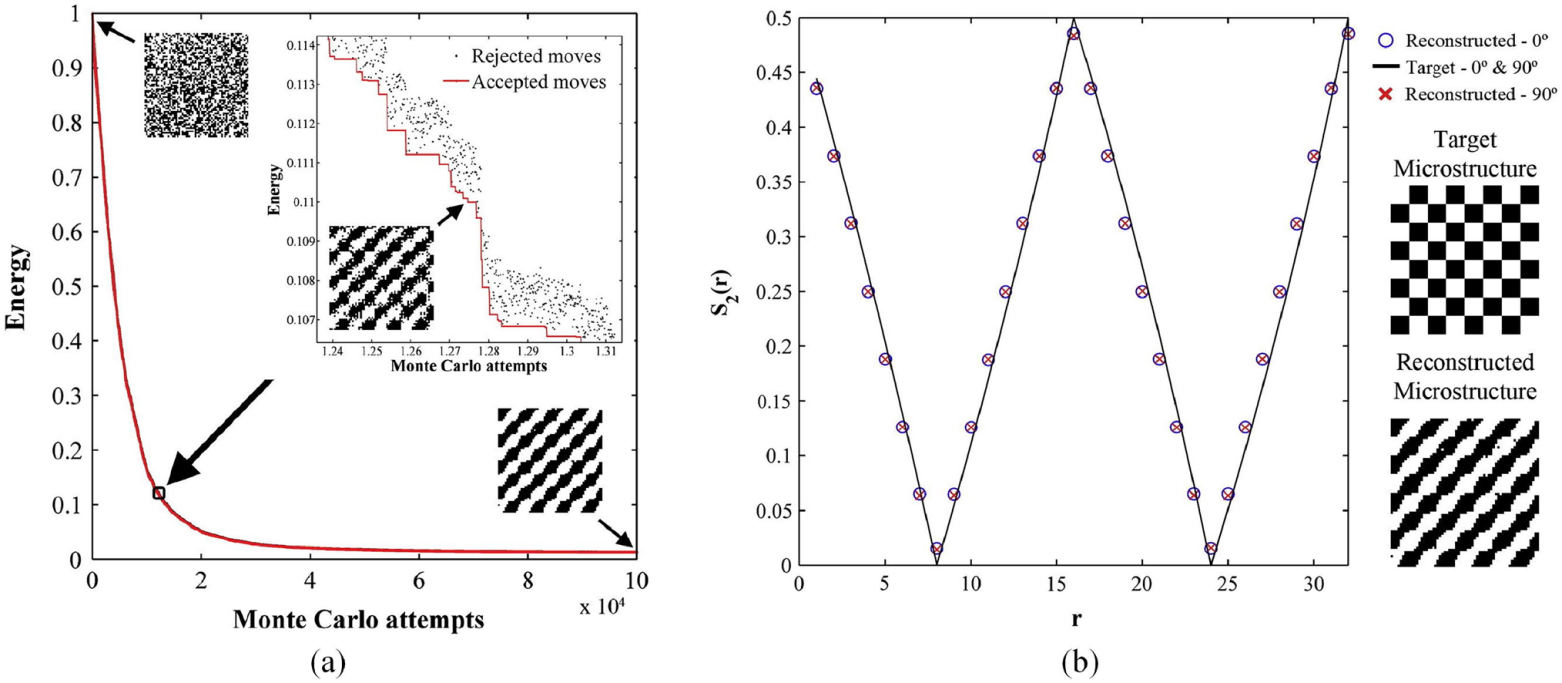
(**a**) Evolution of energy function $\mathcal {E}$ during the process of iteratively switching pixel values of different phases; and (**b**) Two-point correlation function $S_2(r)$ is set as the optimization objective for stochastic microstructure reconstruction [152]

The goal of the optimization procedure is to minimize the energy function $\mathcal {E}$ by iteratively adjusting the phase values of the pixels or voxels within the trial virtual microstructure [151–153], as schematically illustrated in Fig 5. During this iterative process, the trial realization gradually evolves, reducing the statistical discrepancy with the target features. After a sufficient number of iterations, the trial realization converges to the desired statistical properties. At this stage, the reconstructed microstructure can be considered statistically equivalent to the real microstructure, effectively replicating its key features while accommodating inherent stochastic variability.

**Fig. 6:**
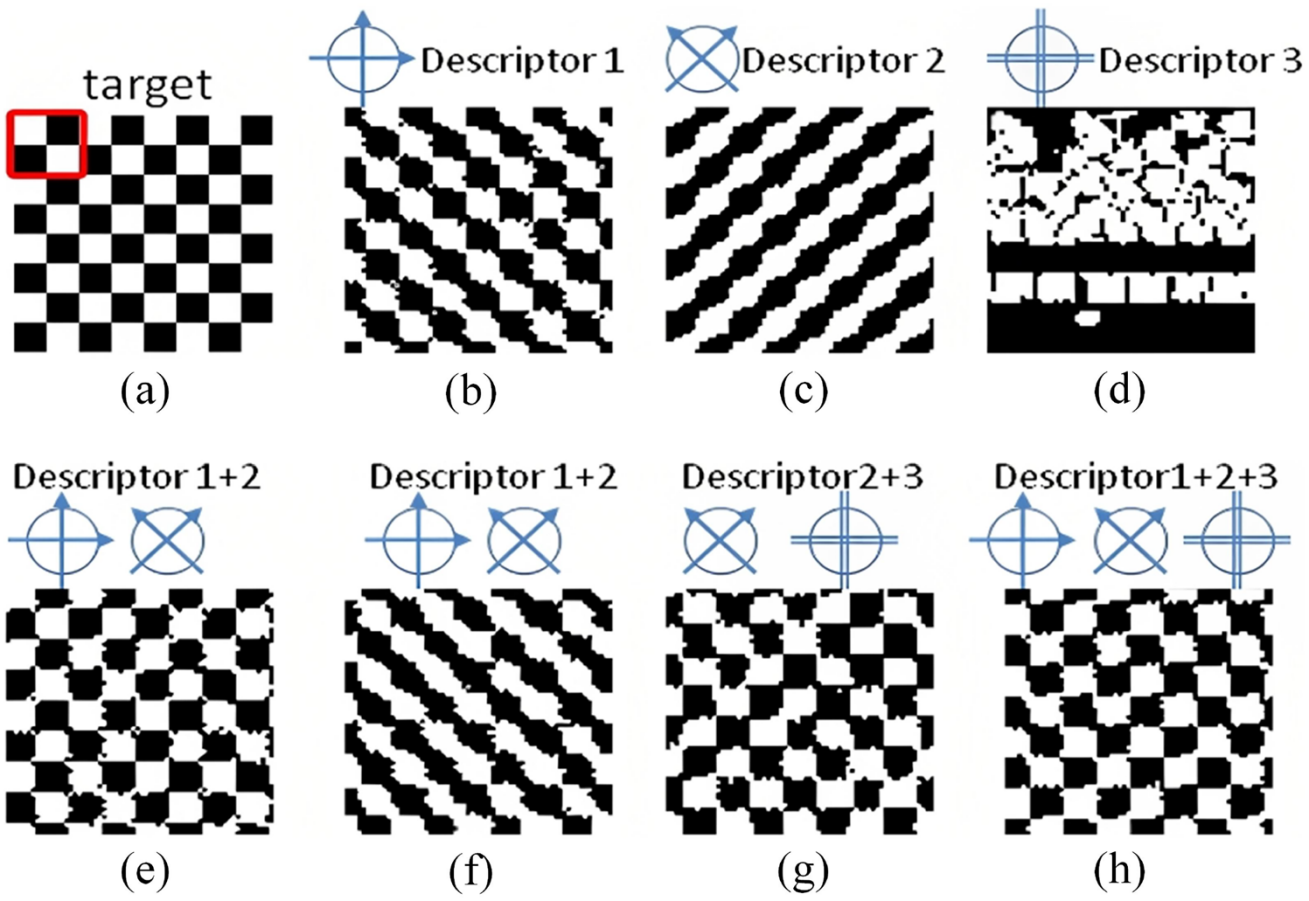
(**a**) The target microstructure; and (**b**)-(**h**) The reconstructed microstructures with different target descriptors [152]

SOR offers remarkable flexibility in defining the optimization objectives by allowing the incorporation of any desired statistical descriptors or their combinations [154–156]. This enables users to determine which statistical characteristics of the real microstructure will be preserved in the reconstructed samples [157–159], at least in principle. As illustrated in Fig 6, three distinct statistical descriptors are extracted from the real microstructure and employed as the target statistical features for SOR. Each descriptor captures unique statistical information, leading to reconstructed microstructures with varying morphological patterns. This diversity highlights the impact of different descriptors on the appearance and characteristics of the reconstructed samples. In general, the inclusion of more comprehensive statistical information in the optimization objective results in reconstructed microstructures that closely resemble the real microstructure. By capturing a broader range of microstructural informatics, the fidelity of the reconstructed samples improves, making SOR a powerful and adaptable tool for stochastic microstructure reconstruction.

To date, the simulated annealing algorithm [84, 108, 160, 161] has remained the most widely used optimizer for driving the iterative procedure in SOR. In addition, alternative stochastic optimization tools, such as the genetic algorithm [110, 162] and the particle swarm algorithm [111], have also been integrated into SOR, offering flexibility and diversity in the optimization approach. Further advancements in SOR have incorporated various computational techniques to improve reconstruction efficiency and accuracy. These developments include multi-scale techniques [112, 163, 164], GPU-based parallel computing [109], dilation-erosion processing [165, 166], stochastic fusion [167], and multi-stage reconstruction [168]. These enhancements address computational challenges, enabling SOR to handle more complex microstructures with improved performance.

Moreover, SOR has been successfully combined with other reconstruction approaches to create hybrid methods, such as the process-based method [169, 170] and the random field transformation method [153, 155]. In these hybrid methods, SOR is typically used to refine the initial microstructure realizations generated by other techniques. Compared to standalone SOR, these hybrid methods are significantly more efficient. The initial microstructure realizations from the process-based or random field transformation methods provide high-quality starting points, reducing the number of iterations required for convergence. As a result, hybrid methods effectively balance computational cost and accuracy, making them a practical and powerful solution for stochastic microstructure reconstruction.

### Random Field Transformation

Random field transformation (RFT) was first introduced for 2D reconstruction of porous microstructures by Joshi (1974) [171], and later extended to 3D microstructure reconstruction by Quiblier (1984) [95] and Adler et al. (1990) [172]. The fundamental concept of RFT is to model the porous microstructure as a second-order stationary random field, described by the following equations:

2 $$\begin{aligned} \left\{ \begin{aligned}&\phi ={E}\Big [\mathcal {I}({\textbf {x}})\Big ]\,,\\&\sigma ^2={E}\left\{ \Big [\mathcal {I}({\textbf {x}})-\phi \Big ]^2\right\} \,,\\&R_z({\textbf {r}})=\frac{{E}\bigg \{\Big [\mathcal {I}({\textbf {x}})-\phi \Big ]\Big [\mathcal {I}({\textbf {x}}+{\textbf {r}})-\phi \Big ]\bigg \}}{\sigma ^2}\,;\\ \end{aligned} \right. \end{aligned}$$

where $\phi$ denotes the porosity (volume fraction); $\mathcal {I}({\textbf {x}})$ is the indicator function to represent the (binary) porous microstructure; ${\textbf {x}}$ denotes the spatial coordinates of image pixels/voxels; $E(\cdot )$ is the expectation operator; $\sigma ^2$ is the variance; and $R_z({\textbf {r}})$ is the autocorrelation function, describing the spatial correlation between microstructural features separated by a length vector ${\textbf {r}}$.

The procedure of stochastic microstructure reconstruction via linear transformation of Gaussian random field is summarized as follows [95, 116, 171]:

- Step 1: Extract the autocorrelation function $R_z({\textbf {r}})$ from the real porous microstructure ${\boldsymbol{Z}}$;
- Step 2: Generate a Gaussian noise field of size $l\times w\times h$ as the initial guess ${\boldsymbol{Y}}_0$, with a mean of 0 and a variance of 1;
- Step 3: Compute the linear filter ${\boldsymbol{a}}$, based on the relationship between the reference autocorrelation function $R_z({\textbf {r}})$ and the autocorrelation function $R_y({\textbf {r}})$ of the underlying Gaussian random field ${\boldsymbol{Y}}$, and then construct the ${\boldsymbol{Y}}$ from ${\boldsymbol{Y}}_0$:
  3 $$\begin{aligned} {\boldsymbol{Y}}(i ,j ,k) = \sum _r\sum _s\sum _t{\boldsymbol{a}}(r,s,t)\,{\boldsymbol{Y}}_0(i+r, j+s, k+t)\,, \end{aligned}$$
- Step 4: Convert the Gaussian random field ${\boldsymbol{Y}}$ into a discrete-valued field ${\boldsymbol{Y}}_{\textrm{d}}$ through a level-cut operation:
  4 $$\begin{aligned} {\boldsymbol{Y}}_{\textrm{d}}(i, j, k)=\left\{ \begin{aligned}&1,\ \ \ \text {if}\ f\big ({\boldsymbol{Y}}(i, j, k)\big )\le \phi \,,\\&0,\ \ \ \text {otherwise}\,;\\ \end{aligned} \right. \end{aligned}$$
  where $f(y)=\frac{1}{\sqrt{2\pi }}$ $\int _{-\infty }^{y}{e}^{-y/2}{\textrm{d}}y$ is the cumulative probability density function of ${\boldsymbol{Y}}$.

The reconstructed microstructure sample ${\boldsymbol{Y}}_{\textrm{d}}$ will have a porosity $\phi$, a standard deviation $\sqrt{\phi -\phi ^2}$, and an autocorrelation $R_z({\textbf {r}})$. Consequently, the reconstructed ${\boldsymbol{Y}}_{\textrm{d}}$ is statistically equivalent to the real microstructure ${\boldsymbol{Z}}$ in terms of both first-order and second-order statistics.

In the procedures described above, the most complex and computationally demanding task is calculating the matrix of the linear filter ${\boldsymbol{a}}$ in Eq. (3), as it involves solving a large system of nonlinear equations [95, 171]. To enhance reconstruction efficiency and accuracy, numerous variations of the original RFT method have been proposed. For instance, Ioannidis et al. (1997) [173] and Liang et al. (1998) [83] employed discrete Fourier transformation (DFT) to directly derive the correlated random field ${\boldsymbol{Y}}$ from its autocorrelation function, thereby avoiding the need to calculate the linear filter ${\boldsymbol{a}}$ in Eq. (3). Grigoriu (2003) [113] introduced the filtered Poisson field as a replacement for the Gaussian random field to model two-phase microstructures. Gao et al. (2021) [174] developed an explicit reconstruction method based on a second-order non-Gaussian random field for hyperuniform biphase materials, which eliminates the need for additional tuning or iterative procedures. Furthermore, Li et al. (2023) [93] integrated the watershed algorithm into the RFT-based reconstruction, aiming to identify and eliminate the isolated solid clusters.

In addition, nonlinear transformation of Gaussian random field has also been developed to generate porous microstructure samples, where iterative algorithms are employed to construct the underlying Gaussian random field ${\boldsymbol{Y}}$ [114, 175, 176]. The process begins with an initial power spectral density (PSD) structure, and an iterative algorithm continuously updates the PSD of the Gaussian field until the target is met. However, the most computationally expensive aspect of this approach lies in determining the non-negative definite covariance function of the correlated Gaussian field, making it slow for practical applications. To address these challenges, Feng et al. (2014, 2016) [115, 177] proposed a new nonlinear transformation algorithm that significantly improves reconstruction efficiency. In this method, the relationship between the correlation of the binary field and the Gaussian field is derived explicitly, eliminating the need for the costly iterative procedure.

### Process-Based Reconstruction

Process-based reconstruction (PR) was initially introduced by Bakke and Øren (1997) [117] for constructing 3D microstructures of porous sandstones. Since its inception, various enhancements and adaptations have been proposed by researchers such as Biswal et al. (1999) [118], Kainourgiakis et al. (2000) [119], Øren and Bakke (2002) [97], Kikkinides et al. (2003) [178], and Siddique et al. (2012) [120]. The fundamental idea of PBR is to stochastically reconstruct 3D rock microstructures by mimicking the key geological formation processes, including sedimentation, compaction, and diagenesis. Petrographical information essential for stochastic microstructure reconstruction, such as porosity and grain size distribution, is derived from 2D thin sections of actual rock samples through image analysis.

**Fig. 7:**
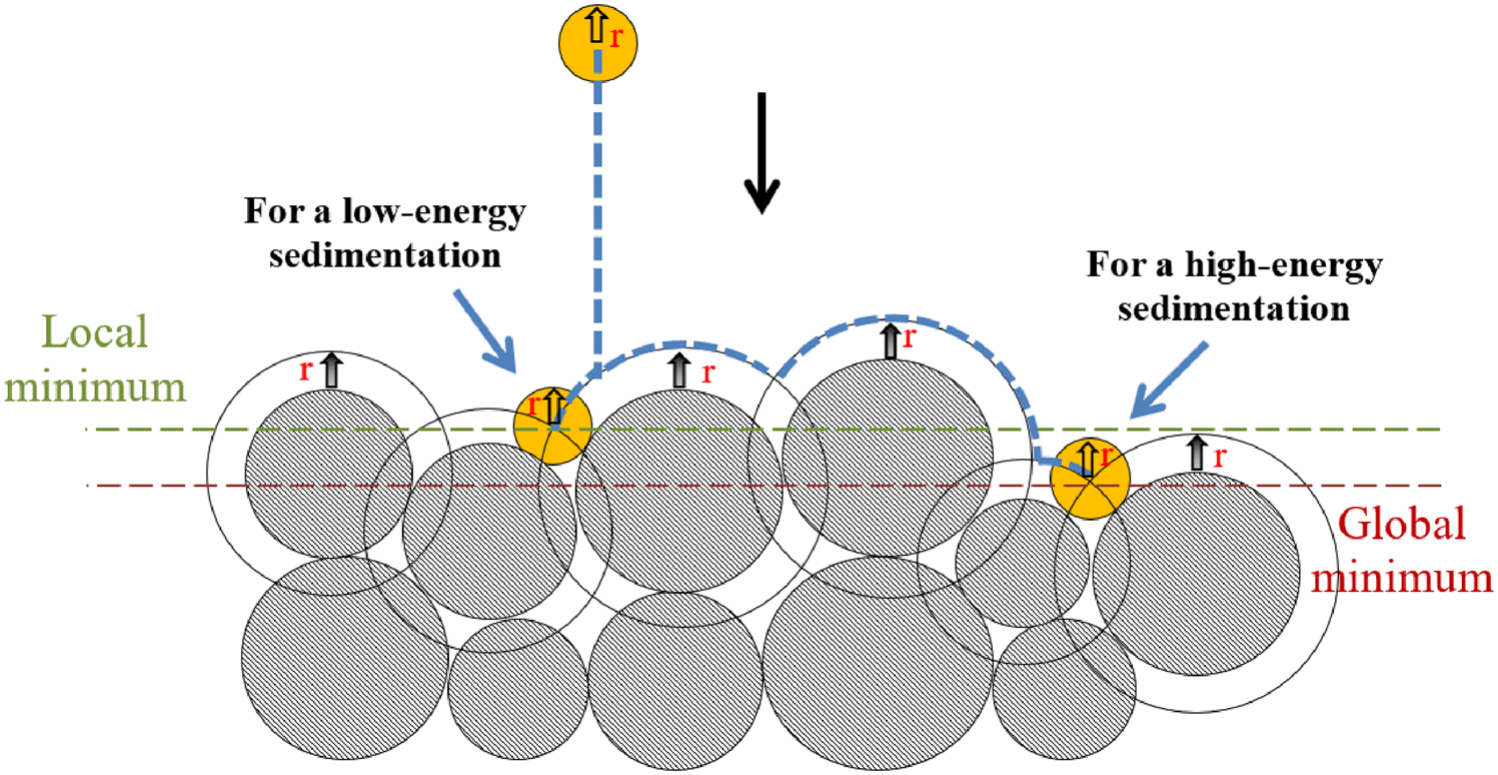
Schematic illustration of grain sedimentation process
- **Compaction process**: Following sedimentation, the grains undergo a compaction process to simulate the effects of vertical stress from overburden. This process alters the positions of grains according to the following linear rule:
  5 $$\begin{aligned} z=z_0\big (1-\beta _z\big )\,, \end{aligned}$$
  where $z_0$ denotes the vertical position of the grain at the initial state, *z* denotes the vertical position after compaction, and $\beta _z$ characterizes the degree of compaction (as illustrated in Fig 8);

The classical PBR proposed by Øren and Bakke (2002) [97] provides a structured procedure for generating 3D rock microstructures, and its main steps are summarized as follows:

- **Extracting petrographical information**: Key petrographical parameters are derived from real rock microstructures through image analysis. This includes data on porosity, grain size distribution, the amount of cement and clay, and the degree of compaction. These measurements serve as the foundation for accurately simulating the geological processes involved.
- **Grain sedimentation process**: Using the measured grain size distribution, grains are stochastically selected and deposited into a predefined bounding box to simulate sedimentation. This is achieved through a sequential deposition algorithm, where grains are dropped randomly while adhering to the constraints of the grain size distribution (as illustrated in Fig 7).

**Fig. 8:**
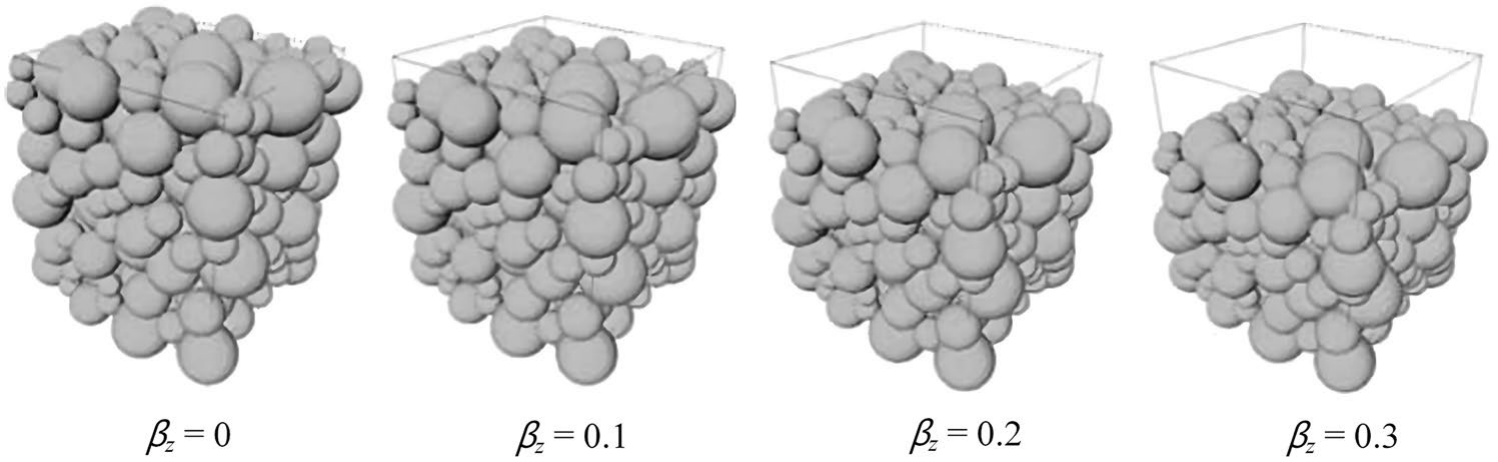
Linear compaction modeling with different degree of compaction [97]
- **Diagenesis process**: In this step, quartz cement overgrowth is applied to the compacted model to simulate the natural geological process of cementation. This process is governed by the following equation:
  6 $$\begin{aligned} R({\textbf {r}})=R_0+\textrm{min}\Big [\alpha l({\textbf {r}})^{\gamma },\,l({\textbf {r}})\Big ]\,, \end{aligned}$$
  where $R_0$ denotes the original grain radius, $l({\textbf {r}})$ is the distance between the original grain surface and the Voronoi polyhedron surface along the direction ${\textbf {r}}$, $\alpha$ is the factor controlling the amount of cement growth, and the coefficient $\gamma$ controls the direction of cement growth. The process captures the natural variability of cement growth in different directions, as illustrated in Fig 9.

**Fig. 9:**
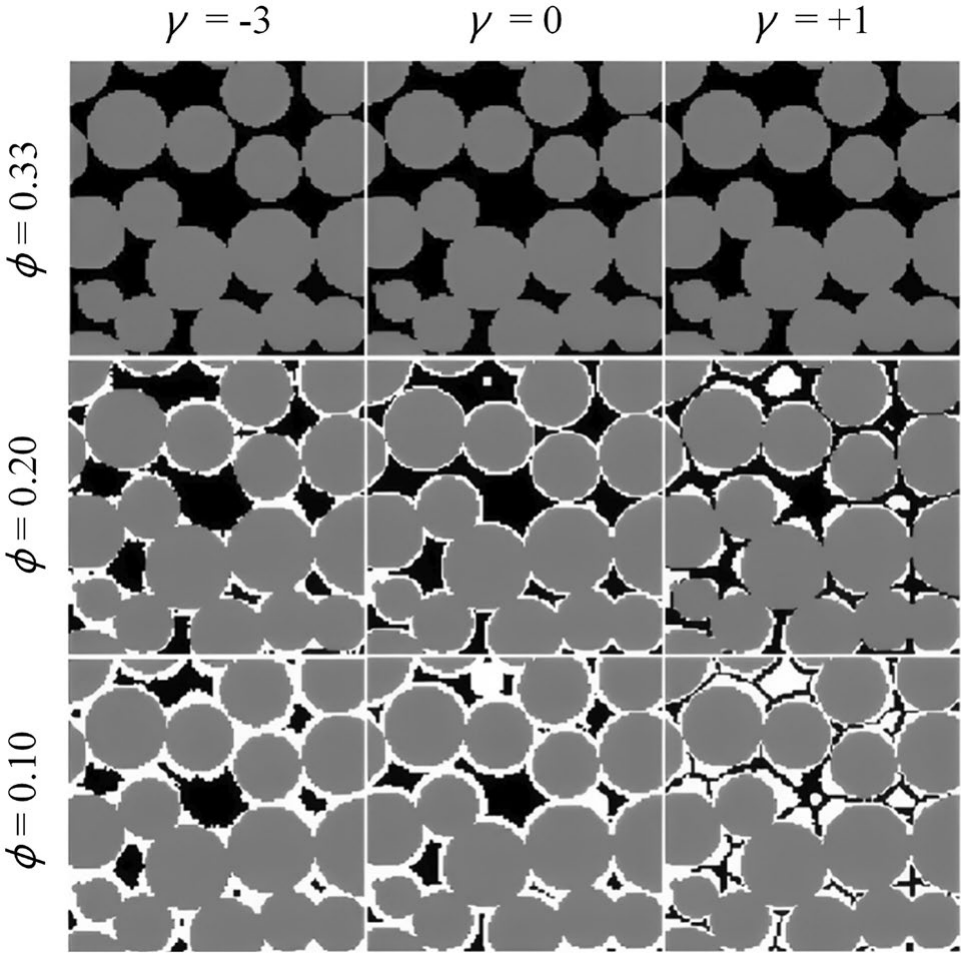
Graphical illustration of quartz cement overgrowth (white) at different values of porosity $\phi$ and orientation coefficient $\gamma$ [97]

Early implementations of PBR commonly relied on simplified sequential deposition algorithms, where particles were randomly placed according to prescribed grain size distributions without accounting for inter-particle interactions. While computationally efficient, these models often failed to reproduce realistic porosity and grain arrangement due to their inability to mimic the physics of sedimentation, such as collision, rolling, and rearrangement of grains. To address these shortcomings, discrete element method (DEM)-based approaches have been increasingly integrated into PBR frameworks [179–181]. DEM explicitly simulates particle–particle contact forces and motion under gravity or other applied loads, enabling dynamic rearrangement and compaction that more faithfully replicates natural or industrial sedimentation processes [60, 61]. As a result, DEM-enhanced PBR can generate more mechanically consistent and statistically accurate microstructures, particularly for granular materials where contact mechanics significantly influence pore morphology and connectivity.

### Random Set-Based Reconstruction

Random set [182] is a mathematical model to represent irregular shapes in mathematical morphology, stochastic geometry and spatial statistics. As the most popular random set model, the Boolean model [121–124] has been applied to stochastically generate porous microstructure samples. A Boolean model *X* is defined as the union of independent objects $A_x$ located at the Poisson point $\mathcal {P}$ [183]:

7 $$\begin{aligned} X = \bigcup _{x\, \in \mathcal {P}}A_x\,, \end{aligned}$$

where $A_x$ denotes the object located at *x*.

The basic procedure of stochastic microstructure reconstruction using the Boolean model is summarized as follows:

- Step 1 is a Poisson point process $\mathcal {P}$ to determine the spatial distribution of Poisson points;
- Step 2 is to place a family of mutually independent objects $A_x$ at the locations of Poisson points.

The random objects are generated based on the statistical information extracted from the real microstructures, such as particle/grain size distribution, porosity, pore-solid surface area, and Euler characteristics.

**Fig. 10:**
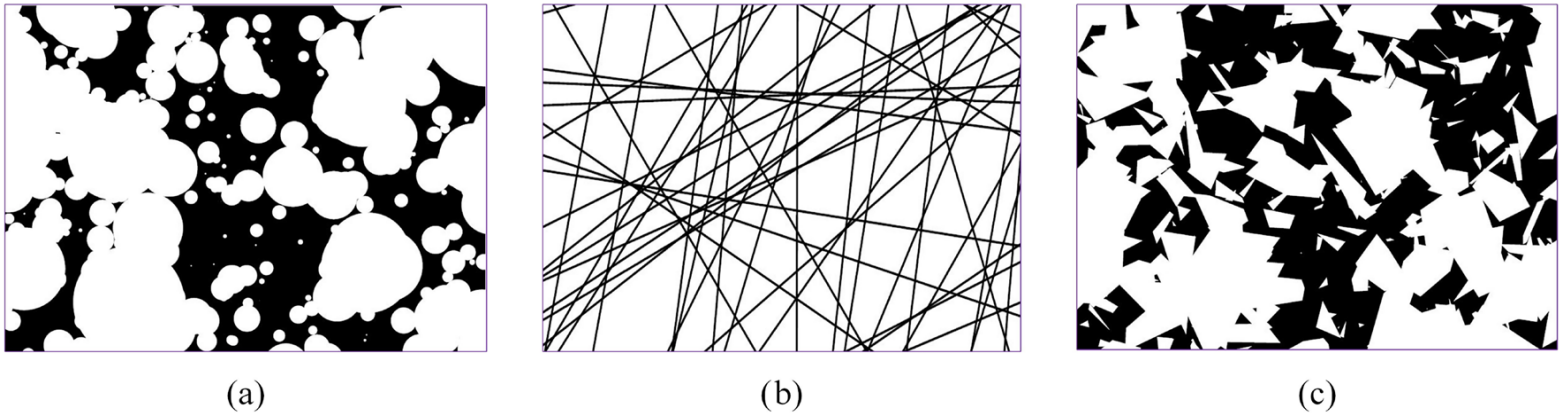
Boolean models [183] of (**a**) Poisson discs, (**b**) Poisson polygons, and (**c**) Poisson lines

Random set-based reconstruction is generally straightforward and computationally efficient. It is well-suited for random media with relatively simple morphologies, such as composites containing spherical, fibrous, or polygonal inclusions, as well as Voronoi cell microstructures (see Fig 10). However, for random porous microstructures with more intricate geometries, the placement and manipulation of random set objects become significantly more challenging. This complexity can result in reduced efficiency and diminished applicability for such cases.

### Spinodoid Topology Generation

Spinodal decomposition [71, 145, 146] has been becoming an emerging methodology to randomly generate porous microstructures with bio-inspired characteristics. Spinodoid topologies are inspired by microstructures formed during spinodal decomposition, a phase separation process governed by the Cahn–Hilliard equation [184, 185]. To bypass computationally intensive phase-field simulations, a Gaussian random field (GRF) approximation is adopted for the phase concentration $\varphi ({\boldsymbol{x}})$ at position ${\boldsymbol{x}}$:

8 $$\begin{aligned} \varphi ({\boldsymbol{x}}) = \sqrt{\frac{2}{N}} \sum _{i=1}^{N \gg 1} \cos \Big (\beta {\boldsymbol{n}}_i \cdot {\boldsymbol{x}} + \gamma _i\Big )\,, \end{aligned}$$

where *N* is the number of superimposed plane waves with wavenumber $\beta$, while ${\boldsymbol{n}}_i \sim \mathcal {U}\Big (S^2\Big )$ and $\gamma _i \sim \mathcal {U}\Big (\big [0, 2\pi \big )\Big )$ represent uniformly distributed wavevector orientations and phase angles, respectively. The GRF yields a stochastic, isotropic bicontinuous topology when wavevectors ${\boldsymbol{n}}_i$ are uniformly distributed on the unit sphere $S^2$.

**Fig. 11:**
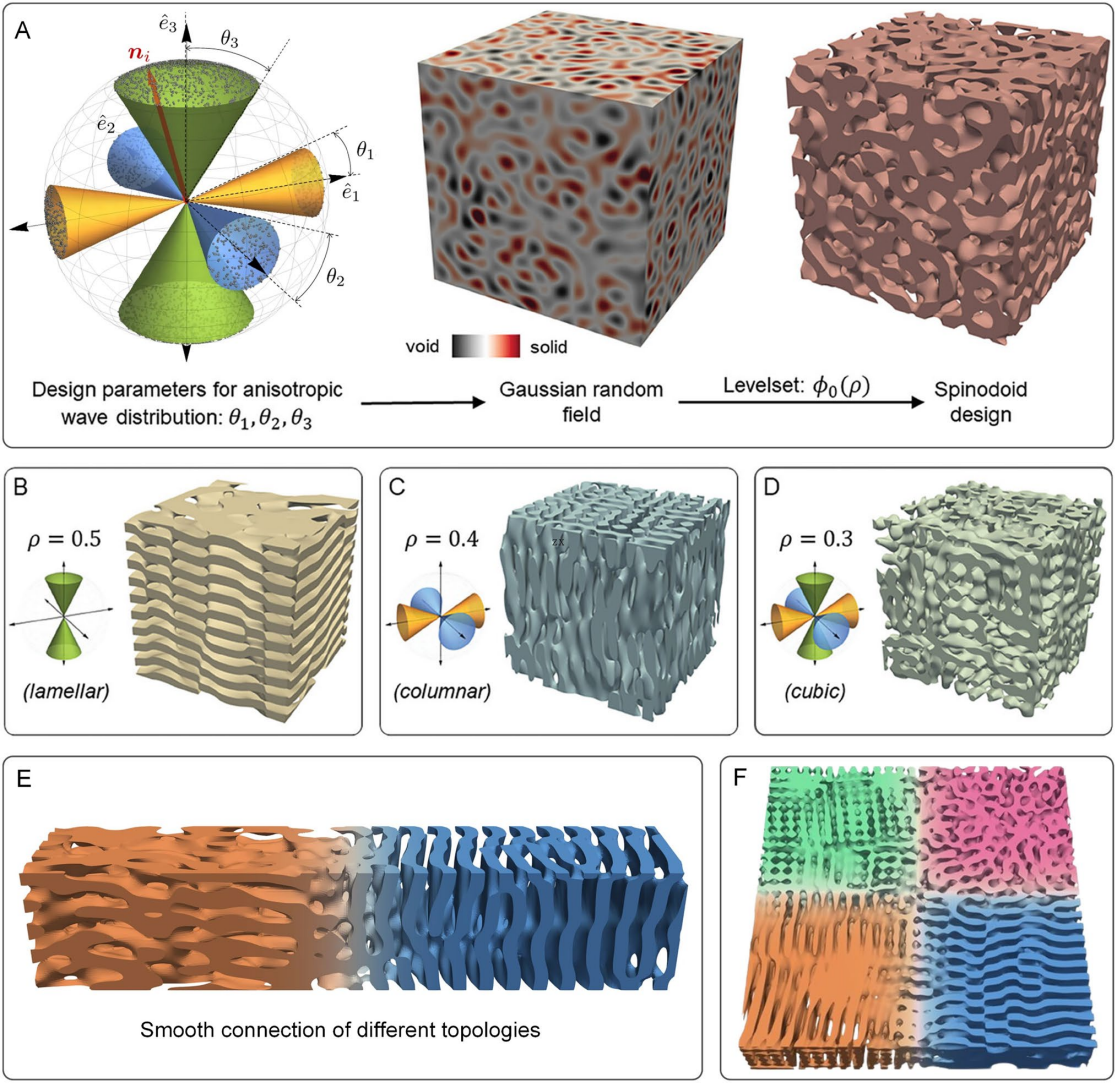
The workflow of spinodoid topology generation and representative 3D microstructure samples [71, 146]

To introduce anisotropy, the wavevector orientations are constrained within conical regions aligned with the Cartesian axes $\big \{\hat{{\boldsymbol{e}}}_1, \hat{{\boldsymbol{e}}}_2, \hat{{\boldsymbol{e}}}_3\big \}$. The modified orientation distribution function is defined as [147, 148]:

9 $$\begin{aligned} {\boldsymbol{n}}_i \sim \mathcal {U}\Bigg (\bigg \{{\boldsymbol{k}} \in S^2 : \bigcup _{j=1}^3 \left( \big |{\boldsymbol{k}} \cdot \hat{{\boldsymbol{e}}}_j\big |> \cos \theta _j\right) \bigg \}\Bigg ), \end{aligned}$$

where $\theta _1, \theta _2, \theta _3 \in [0] \cup [\theta _{\min }, \pi /2]$ are cone angles controlling directional bias (see Fig 11A). Smaller angles restrict wavevectors closer to the corresponding axis, inducing anisotropic microstructures.

Porous microstructures are generated by thresholding the GRF with a level set $\varphi _0$:

10 $$\begin{aligned} \xi ({\boldsymbol{x}}) = {\left\{ \begin{array}{ll} 1 & \text {if } \varphi ({\boldsymbol{x}}) \le \varphi _0, \\ 0 & \text {otherwise}, \end{array}\right. } \end{aligned}$$

where $\varphi _0 = \sqrt{2}\, \textrm{erf}^{-1}(2\rho - 1)$ maps the relative density $\rho$ to the GRF’s quantile. This ensures $\rho$ corresponds to the volume fraction of the solid phase. To avoid disjoint domains, $\rho \ge \rho _{\min } = 0.3$ and $\theta _j \ge \theta _{\min } = \pi /6$. The design parameters ${\boldsymbol{\Theta }} = (\rho , \theta _1, \theta _2, \theta _3)^\textsf{T}$ enable seamless grading and tunable anisotropy. By varying $\theta _j$, distinct topology classes emerge: lamellar, columnar, cubic and isotropic. This parametric framework [4, 186] efficiently explores the design space while ensuring smooth spatial transitions, as shown in Fig 11B–D.

Generally, porous microstructures with spinodoid topology provide the following distinct advantages over periodic cellular microstructures:

- Enhanced mechanical resilience: With nearly zero mean curvature, spinodoid microstructures are designed to minimize stress concentrations, reducing the risk of localized junction failure.
- Superior mass-transport performance: Their highly interconnected pore networks enable efficient fluid and mass transport, enhancing permeability and diffusion properties.
- Diverse bio-inspired morphologies: A wide range of spinodoid topologies can be efficiently generated to mimic the complex microstructural characteristics of natural structures or tissues.

### Merits and Limitations

As listed in Table 1, traditional algorithm-based reconstruction methods offer distinct advantages for specific random microstructures in terms of characterization accuracy and reconstruction efficiency. For instance, the SOR approach [84, 108, 112] provides high flexibility by allowing any combination of statistical descriptors as optimization objectives, enabling the incorporation of various statistical features into reconstructed microstructures. However, its iterative stochastic nature makes it highly time-consuming and computationally intensive, as statistical descriptors must be repeatedly calculated at each step. Additionally, reconstruction quality depends on the initial guess, and no optimization algorithm guarantees global convergence. The RFT approach [95, 113, 115] offers high reconstruction efficiency, but the reconstructed microstructures are limited to preserving first- and second-order statistics, which is far from sufficient to capture the complex microstructural features of heterogeneous porous materials. The PR method [97, 118, 120] shows promise in generating rock microstructures by maintaining long-range pore connectivity. However, it is primarily effective for porous sandstones with well-defined granular matrices and struggles with porous rocks that have undergone complex geological transformations. The STG method [71, 145, 146] is restricted in its ability to generate diverse topologies, primarily producing lamellar, columnar, cubic, and isotropic microstructures. The lack of a clear framework for selecting the design parameters $(\theta _1, \theta _2, \theta _3)$ makes STG difficult to precisely generate microstructure samples with desired morphological features and statistical characteristics.

In general, traditional algorithm-based reconstruction methods are mathematically rigorous and can be well interpreted. As illustrated in Fig 4, these methods explicitly characterize real porous microstructures, enabling the stochastic synthesis of new samples while preserving key microstructural features. By adjusting reconstruction targets or relevant parameters, the preserved characteristics can be systematically modified. However, random porous media exhibit highly complex pore network systems, comprising interconnected, isolated, and dead-end pores with irregular geometries. The pore sizes can span several orders of magnitude, and the pore-solid interfaces are typically rough. These complexities make it extremely challenging, if not impossible, to fully characterize random porous microstructures in an explicit manner. As a result, most traditional reconstruction methods can only generate virtual microstructure samples that are statistically/mathematically equivalent to the real ones, rather than reproduce detail-similar microstructure samples that can be used for MRP analysis through image-based poro/micro-mechanical modeling. Moreover, these methods are primarily designed for bi-phase porous microstructures and struggle with reconstructing multiphase random media [187]. Further advancements are needed to develop a universal approach for efficiently generating high-fidelity porous microstructures while capturing high-order statistical information, geometric intricacies, and topological characteristics.

## Computational Intelligence-Based Microstructure Reconstruction

This section focuses on computational intelligence approaches, which encompass both machine learning and deep learning methods. Machine learning (ML) refers to algorithms that learn patterns from data, often requiring manually engineered features and including models such as support vector machines, decision trees, and shallow neural networks. Deep learning (DL), as a specialized subfield of ML, relies on multi-layer neural networks (such as convolutional neural networks and generative adversarial networks) to automatically extract hierarchical features from raw data. In the context of this review, ML methods are characterized by explicit feature design and statistical modeling, whereas DL methods provide end-to-end learning capabilities suited for complex microstructure reconstruction tasks. Distinctions between these categories are made where relevant to emphasize their respective strengths and application domains.

The rapid advancements in computational intelligence [188, 189] are unlocking new opportunities for stochastic characterization and reconstruction of random porous microstructures. These advancements are driven by explosive progress in AI algorithms, AI processors, data storage, and computing power, making data-driven approaches more accessible and powerful than ever before. One of the most compelling advantages of deep learning algorithms [190, 191] is their ability to operate in a feature-free manner, eliminating the requirement for manual feature design, extraction and selection. This automation not only enhances efficiency but also enables the discovery of intricate, high-dimensional patterns in complex microstructures that would be challenging to capture using traditional algorithms. Due to these advantages, a wide array of computational intelligence-based approaches [77, 101, 103, 104, 192] have emerged for characterizing and reconstructing random porous microstructures. As summarized in Table 2, emerging computational intelligence-based reconstruction methods have been categorized based on the underlying machine/deep learning algorithms employed. In this section, a comprehensive overview of the most representative AI-based microstructure reconstruction methods will be provided, with discussions of their theoretical foundations, algorithmic frameworks, and reconstruction performance. Special attention will be given to their ability to capture complex geometric and topological features, improve computational efficiency, and enhance microstructural fidelity in comparison to traditional approaches.

**Table 2:** Computational intelligence-based methods for stochastic microstructure reconstruction

${\textbf {Machine/deep learning algorithm}}$		${\textbf {Reconstruction type}}$	${\textbf {Validation}}$	${\textbf {Representative references}}$
Support vector machine (SVM)		Dimensionally-upgraded	Morphological descriptors; Physical properties	[100, 193, 194]
Decision tree (DT)		Equidimensional	Morphological descriptors	[101, 195]
Statistics-informed neural network (SINN)		Equidimensional	Morphological descriptors; Physical properties	[192, 196]
		Dimensionally-upgraded	Morphological descriptors; Physical properties	[51, 77, 82]
Convolutional neural network (CNN)		2D-to-2D	Morphological descriptors; Physical properties	[197]
		3D-to-3D	Morphological descriptors; Physical properties	[198–201]
Convolutional deep belief network (CDBN)		2D-to-2D	Morphological descriptors; Physical properties	[102, 202]
Transfer learning (TL)		2D-to-2D	Morphological descriptors	[203–205]
		3D-to-3D	Morphological descriptors; Physical properties	[206]
		Dimensionally-upgraded	Morphological descriptors; Physical properties	[105]
Variational auto-encoder (VAE)		2D-to-2D	Morphological descriptors; Physical properties	[207–210]
		3D-to-3D	Morphological descriptors; Physical properties	[211–213]
Generative adversarial network (GAN) and its variants	Standard GAN	3D-to-3D	Morphological descriptors	[214–218]
		3D-to-3D	Morphological descriptors; Physical properties	[103, 219–221]
		2D-to-2D	Morphological descriptors; Physical properties	[222–224]
		Dimensionally-upgraded	Morphological descriptors; Physical properties	[225–227]
	Conditional GAN (CGAN)	3D-to-3D	Morphological descriptor	[228, 229]
		2D-to-2D	Morphological descriptors	[230–232]
		2D-to-2D	Morphological descriptors; Physical properties	[233–236]
		Dimensionally-upgraded	Morphological descriptors; Physical properties	[237]
	Wasserstein GAN (WGAN)	2D-to-2D	Morphological descriptors	[230, 238]
		3D-to-3D	Morphological descriptors; Physical properties	[239–242]
		Dimensionally-upgraded	Morphological descriptors; Physical properties	[232, 243, 244]
	StyleGAN	2D-to-2D	Morphological descriptors; Physical properties	[245, 246]
		3D-to-3D	Morphological descriptors	[247]
	SAGAN	Equidimensional	Morphological descriptors; Physical properties	[248, 249]
	BicycleGAN	2D-to-2D	Morphological descriptors; Physical properties	[233, 234]
		Dimensionally-upgraded	Morphological descriptors; Physical properties	[250]
	Pix2Pix GAN	3D-to-3D	Morphological descriptors; Physical properties	[251]
	Multi-scale GAN	3D-to-3D	Morphological descriptors; Physical properties	[252–255]
		Dimensionally-upgraded	Morphological descriptors	[256]
Sequence	Recurrent neural networks (RNN)	3D-to-3D	Morphological descriptors; Physical properties	[257–259]
	Generative pre-training	3D-to-3D	Morphological descriptors; Physical properties	[260]
	Transformer	Dimensionally-upgraded	Morphological descriptors; Physical properties	[261]
Generative flow network (GFN)		Dimensionally-upgraded	Morphological descriptors	[262, 263]
Diffusion-based generative model		2D-to-2D	Morphological descriptors; Physical properties	[264]
		Dimensionally-upgraded	Morphological descriptors; Physical properties	[265]
Hybrid modeling	VAE + GAN	2D-to-2D	Morphological descriptors; Physical properties	[266]
		3D-to-3D	Morphological descriptors; Physical properties	[267]
		Dimensionally-upgraded	Morphological descriptors; Physical properties	[268–270]
	AE + GAN	3D-to-3D	Morphological descriptors; Physical properties	[271, 272]
		Dimensionally-upgraded	Morphological descriptors; Physical properties	[273]
	SinGAN + Attention	3D-to-3D	Morphological descriptors; Physical properties	[252–254]
	InfGAN + StyleGAN	3D-to-3D	Morphological descriptors; Physical properties	[274]
	StyleGAN + CycleGAN	2D-to-3D	Morphological descriptors; Physical properties	[275]
	VQVAE + PixelCNN	2D-to-2D	Morphological descriptors	[276]
	WGAN + CNN	3D-to-3D	Morphological descriptors; Physical properties	[277]
		2D-to-2D	Morphological descriptors; Physical properties	[278]
	GAN + Actor-critic (AC)	3D-to-3D	Morphological descriptors	[279]
	CGAN + TSS	Equidimensional	Morphological descriptors; Physical properties	[237]
	RFT + CGAN	Equidimensional	Morphological descriptors; Physical properties	[236]

### General Workflow

**Fig. 12:**
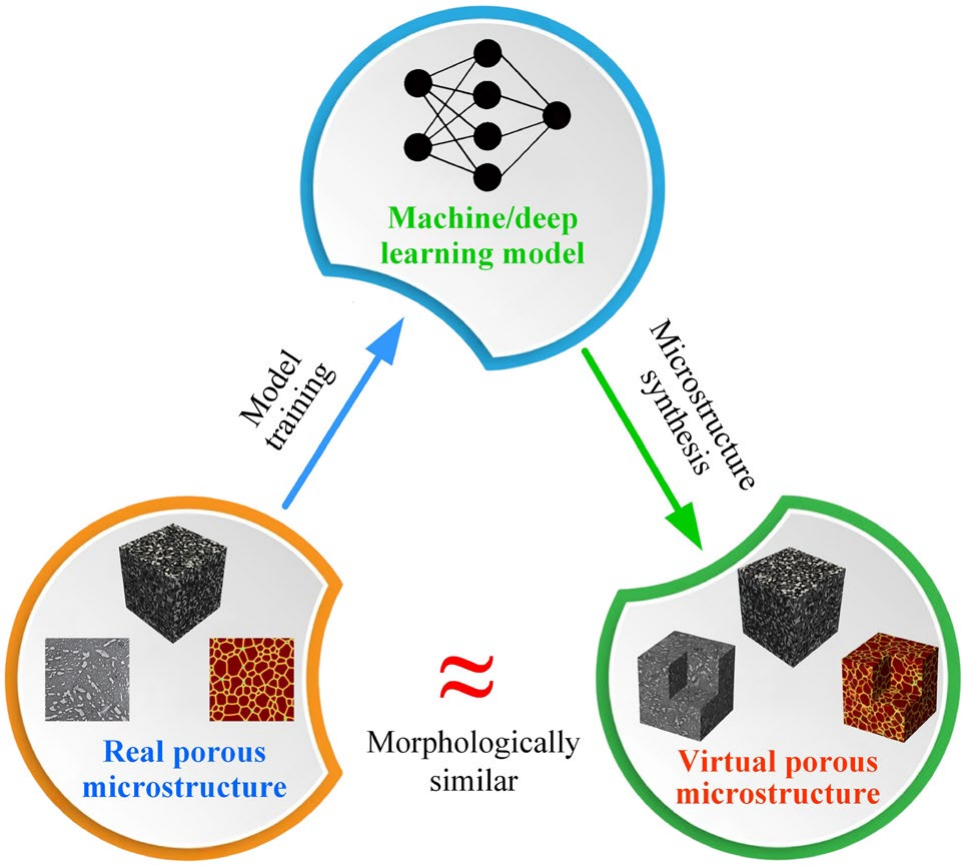
The general workflow of computational intelligence-based methods for stochastic microstructure reconstruction

As illustrated in Fig 12, computational intelligence-based methods for stochastic microstructure reconstruction follow a two-stage workflow:

- **Machine learning-based characterization**: This phase involves training machine/deep learning models to quantitatively characterize real microstructures. These models learn and encode essential morphological features in an implicit and often inexplicable manner, capturing high-dimensional characteristics without relying on explicit mathematical descriptors.
- **Microstructure synthesis**: Once trained, these machine/deep learning models can function as an information library or a generative tool, enabling stochastic synthesis of virtual microstructure samples while preserving and reproducing the morphological patterns learned from the real microstructures.

Regarding to microstructural characterization, simple machine learning models, such as support vector machine [100, 193], decision trees [101, 195] and feedforward neural networks [77, 192], rely on manual feature design and extraction. This requires domain expertise to identify and encode critical microstructural attributes (e.g., porosity, surface area density, and multi-point statistics) as the input features. In contrast, deep learning models such as generative adversarial networks [103, 250] and convolutional neural networks [104, 276] leverage multiple convolutional layers to automatically extract hierarchical morphology features from raw digital microstructure images. This eliminates the need for manual feature selection and allows deep learning models to characterize complex morphological patterns directly from image data.

### Support Vector Machine-Based Reconstruction

Support vector machine (SVM) [100, 193] represents one of the earliest attempts to apply computational intelligence for the stochastic characterization and reconstruction of heterogeneous microstructures. This method relies on a pre-constructed library of 3D microstructures with diverse morphological characteristics, which can be generated using Monte Carlo techniques [280]. Given a 2D thin-section image, the SVM-based approach enables the reconstruction of statistically equivalent 3D microstructure samples through the following four key steps:

- Database creation: Construct a comprehensive microstructure library containing a wide range of 3D porous microstructures with varying morphological features;
- Feature extraction: Extract morphological descriptors (e.g., porosity, autocorrelation function, grain size distribution) from both the input 2D target image and the microstructure database;
- SVM model training: Train an SVM classifier to learn correlations between the 2D image descriptors and the stored 3D microstructures;
- Microstructure searching: Use the trained SVM model to identify and retrieve the most statistically equivalent 3D microstructure(s) from the database.

**Fig. 13:**
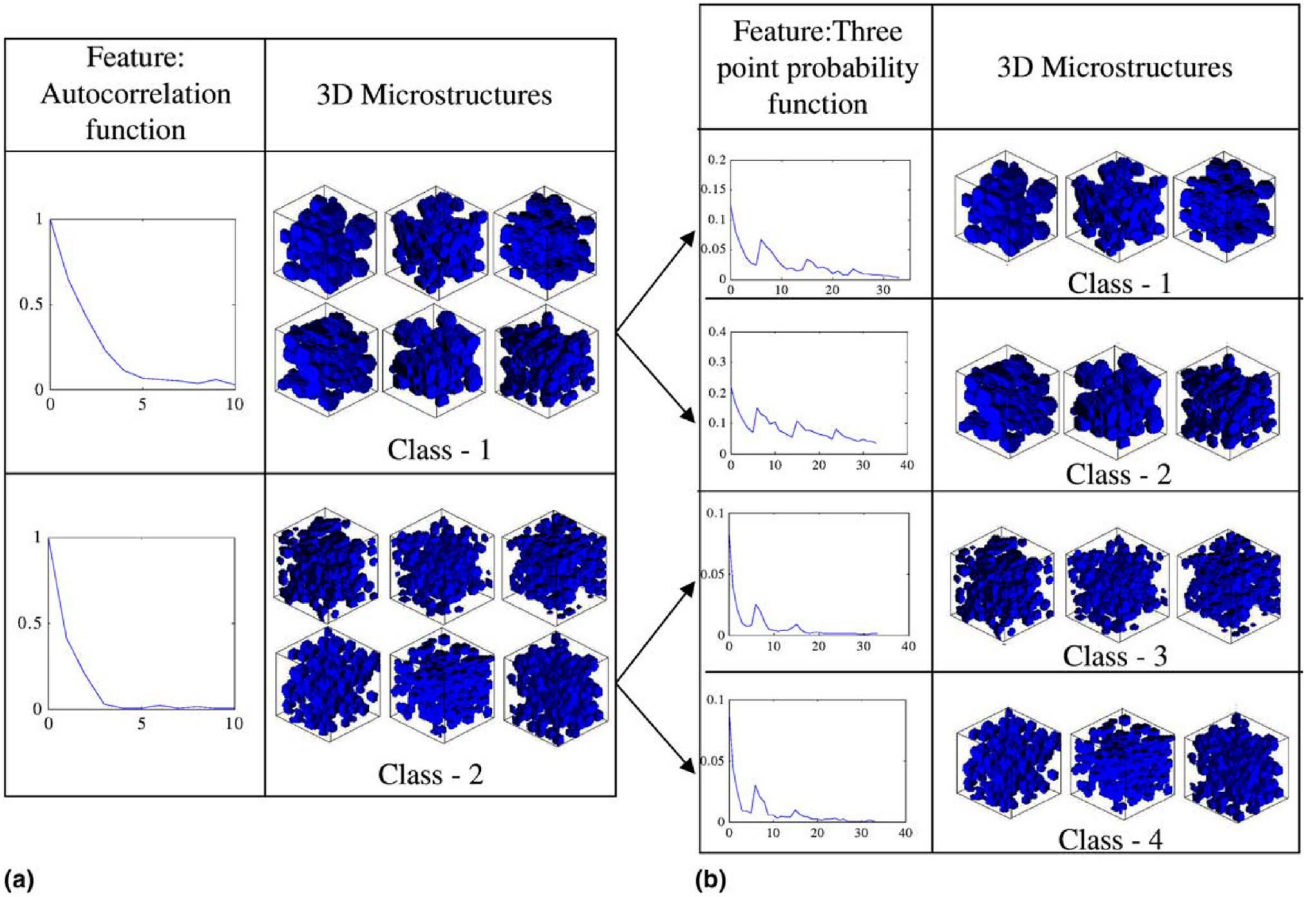
Hierarchical multi-class classification in SVM-based microstructure reconstruction: (**a**) Level 1: 3D microstructure classes based on autocorrelation function; (**b**) Level 2: sub-classes based on three-point probability function [100]

SVM is a powerful supervised learning algorithm that can map input data ${\textbf {x}}$ to a higher-dimensional feature space using a transformation function, ${\textbf {z}}=\mathcal {H}({\textbf {x}})$. In this transformed space, an optimal hyperplane is constructed to achieve non-linear separation of the data. Within the SVM-based microstructure reconstruction framework, the trained model serves as a classifier, ensuring that the selected 3D microstructures from the database closely match the desired statistical features extracted from the input 2D thin-section image, as illustrated in Fig 13. The goal of training an SVM model is to determine the optimal hyperplane that separates different microstructure classes. This hyperplane is represented by the following equation:

11 $$\begin{aligned} {\textbf {w}}\cdot \mathcal {H}({\textbf {x}})+b=0 \,, \end{aligned}$$

where ${\textbf {w}}$ is the weight vector that defines the orientation of the hyperplane; *b* is the bias term, which shifts the hyperplane; and $\mathcal {H}({\textbf {x}})$ represents the mapping function that transforms input features into a higher-dimensional space. Once the SVM model is trained by optimizing ${\textbf {w}}$ and *b*, it can be used for classification. Given a new microstructural feature vector ${\textbf {x}}^{\textrm{T}}$ to the SVM model yields the sign of ${\textbf {w}}\cdot \mathcal {H}({\textbf {x}})+b$, which indicates the class of 3D microstructures that closely match the input feature. The decision function can be mathematically expressed as follows:

12 $$\begin{aligned} \mathcal {S}\mathcal {V}\mathcal {M}\,\big ({\textbf {x}}\big )={\textrm{sgn}}\Big [{\textbf {w}}\cdot \mathcal {H}({\textbf {x}})+b\Big ] \,, \end{aligned}$$

where $\textrm{sgn}(\cdot )$ returns the sign of the function value, indicating the class of the most statistically equivalent 3D microstructure in the database.

By leveraging SVM classification, this approach enables efficient selection of realistic 3D porous structures from a predefined library, ensuring that the reconstructed samples accurately reflect the morphological and statistical properties of the target microstructure. However, the effectiveness of SVM heavily depends on the quality and diversity of the microstructure database. the selection of kernel functions also has a great influence on enhances the SVM model’s ability to capture complex microstructural patterns. For more complex porous microstructures, deep learning-based methods may offer superior scalability and feature extraction capabilities, overcoming some of SVM’s limitations.

### Decision Tree-Based Reconstruction

Inspired by texture synthesis techniques, Bostanabad et al. [101, 195] introduced a decision tree-based approach for statistical characterization and stochastic reconstruction of random microstructures. In this method, a random microstructure is treated as a realization of a specific stochastic process, where the full joint probability distribution function (PDF) $P({\boldsymbol{X}})$ of pixel/voxel variables serves as an ideal statistical model for microstructure characterization. However, deriving the full joint PDF from a limited number of real microstructure samples is infeasible due to the high dimensionality of the data. To address this challenge, the random microstructure ${\boldsymbol{X}}$ is assumed to follow a stationary Markov random field (MRF), which simplifies the statistical modeling process. Mathematically, the MRF assumption can be expressed as:

13 $$\begin{aligned} P\left( X_{ij}\big |{\boldsymbol{X}}^{(-ij)}\right) =P\,\Big (X_{ij}\big |{\boldsymbol{N}}_{ij}\Big )\,, \end{aligned}$$

where $X_{ij}$ denotes the pixel located at the *i*-th line and *j*-th column of the image ${\boldsymbol{X}}$, ${\boldsymbol{N}}_{ij}$ denotes its surrounding neighboring pixels within a sufficiently large range, and ${\boldsymbol{X}}^{(-ij)}$ denotes the set of all pixels in ${\boldsymbol{X}}$ excluding $X_{ij}$.

**Fig. 14:**
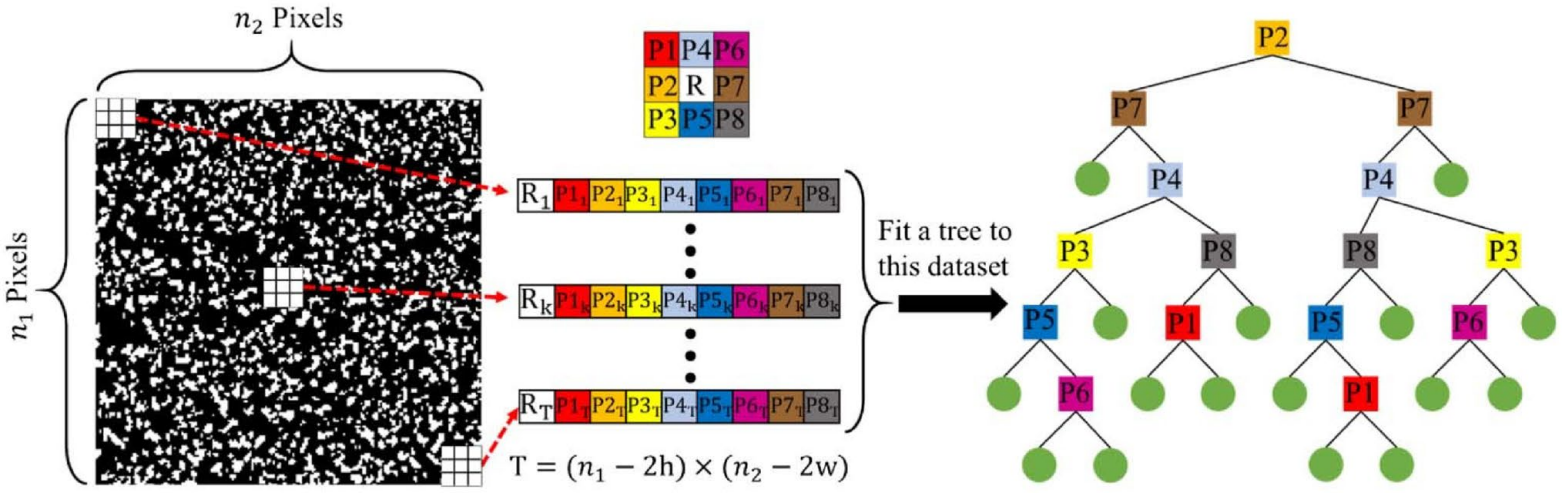
Graphical illustration of statistical microstructure characterization by training a decision tree-based classification model [101]

The key advantage of the MRF framework is its ability to construct a global microstructure representation by leveraging local statistical dependencies. This property enables decision tree-based methods to effectively capture complex microstructural patterns while requiring only a relatively small dataset for training. As illustrated in Fig 14, a predefined data template scans the training image to collect local morphology patterns. The collected data events are paired observations, which contain the phase values of the central pixel and their neighborhoods. These data events can be used as labeled training data to fit a decision tree (DT) model via supervised learning, where the response variable is the phase value of the central pixel and the predictor variables are the phase values of its surrounding neighborhoods. The class probability stored in the fitted decision tree model is an accurate approximation of the conditional PDF. The set of conditional distributions embedded in the decision tree model provides an implicit representation of the full joint distribution, which statistically characterizes the morphology patterns within the training image.

Once the decision tree is properly trained, it can be used to estimate the conditional probability $P\big (X_{ij}|{\boldsymbol{N}}_{ij}\big )$ by inputting any given neighboring phase values. This estimated probability distribution then serves as a basis for generating new pixel values through random sampling. By iteratively applying this procedure, an entirely new microstructure can be synthesized, where the phase values of all pixels are determined sequentially. The overall sequential sampling process follows:

14 $$\begin{aligned} P\big ({\boldsymbol{X}}\big )&=P\big (X_{11}\big )\,P\big (X_{12}|X_{11}\big )\,P\big (X_{13}|X_{11},X_{12}\big )\cdots P\big (X_{ij}|X_{11},X_{12},\cdots X_{i(j-1)}\big )\cdots \\&=P\big (X_{11}\big )\,P\big (X_{12}|{{\boldsymbol{X}}}_{(<12)}\big )\,P\big (X_{13}|{{\boldsymbol{X}}}_{(<13)}\big )\cdots P\big (X_{ij}|{{\boldsymbol{X}}}_{(<ij)}\big )\cdots \,, \end{aligned}$$

This sequential approach ensures that each pixel is generated based on the learned statistical dependencies from the real microstructure, enabling the decision tree-based method to reconstruct microstructures with realistic spatial correlations and morphological characteristics.

The decision tree-based method has been further extended to enable 3D-to-3D microstructure reconstruction [195], demonstrating its ability to generate reconstructed microstructures that closely resemble real ones in terms of morphological patterns. Moreover, it effectively preserves key spatial correlation characteristics of the original microstructure, including the two-point correlation function, two-point cluster correlation function, and lineal path function. However, despite its success in capturing morphological and statistical features, the method has not been rigorously validated in terms of preserving the physical properties of the real microstructures. Additionally, due to the underlying stationary Markov random field (MRF) assumption, this approach may struggle to capture long-range correlations and heterogeneity, which are critical for accurately representing complex random microstructures. Another significant limitation is computational efficiency. The sequential voxel-wise generation process makes the method computationally expensive, particularly for large-scale 3D microstructures that consist of millions of voxels. This inherently high computational cost can significantly hinder its applicability to large and complex microstructure datasets.

### Statistics-Informed Neural Network-Based Reconstruction

Building upon the ideas of texture synthesis and decision tree-based methods, Fu et al. [77, 82, 192] introduced a novel computational framework for stochastic characterization and microstructure reconstruction using supervised learning. This framework integrates morphological statistics $P\left( X_{ij}\big |{{\boldsymbol{N}}}_{ij}\right)$ of random microstructures into a feedforward neural network (FNN). The fitted FNN model is a vector-valued surrogate to approximate the ${{\boldsymbol{N}}}_{ij}$-$X_{ij}$ mapping for the training image ${{\boldsymbol{X}}}$, which can be mathematically expressed as follows:

15 $$\begin{aligned} \mathcal {F}\mathcal {N}\mathcal {N}\Big ({{\boldsymbol{N}}}_{ij};{{\boldsymbol{W}}}, {{\boldsymbol{b}}}\Big ):~ {{\boldsymbol{N}}}_{ij}\in \mathbb {R}^{d_N}\rightarrow X_{ij}\in \mathbb {R}^{d_C}\,, \end{aligned}$$

where $\mathcal {F}\mathcal {N}\mathcal {N}\big (\cdot \big )$ denotes the approximation function of the FNN model. In essence, the FNN model fitted by the data events $\left( X_{ij}, {{\boldsymbol{N}}}_{ij}\right)$ can be considered as an implicit representation of the CPDF $P\left( X_{ij}\big |{{\boldsymbol{N}}}_{ij}\right)$, and it is therefore referred to $\textit{statistics-informed neural network}$ (SINN). The classical MRF assumption only focuses on the relationship between one central pixel/voxel $X_{ij}$ and its surrounding neighboring pixels/voxels ${\boldsymbol{N}}_{ij}$. Fu et al. [192] extended this classical MRF assumption to more general forms, where the correlation between multiple central pixels/voxels ${\boldsymbol{C}}_{ij}$ and their surrounding neighboring pixels/voxels ${\boldsymbol{N}}_{ij}$ can be considered. The purpose of extending the MRF assumption is two-fold: (i) to characterize the long-distance correlations through a hierarchical approach; and (ii) to improve reconstruction efficiency by updating multiple pixels/voxels at each iteration step.

**Fig. 15:**
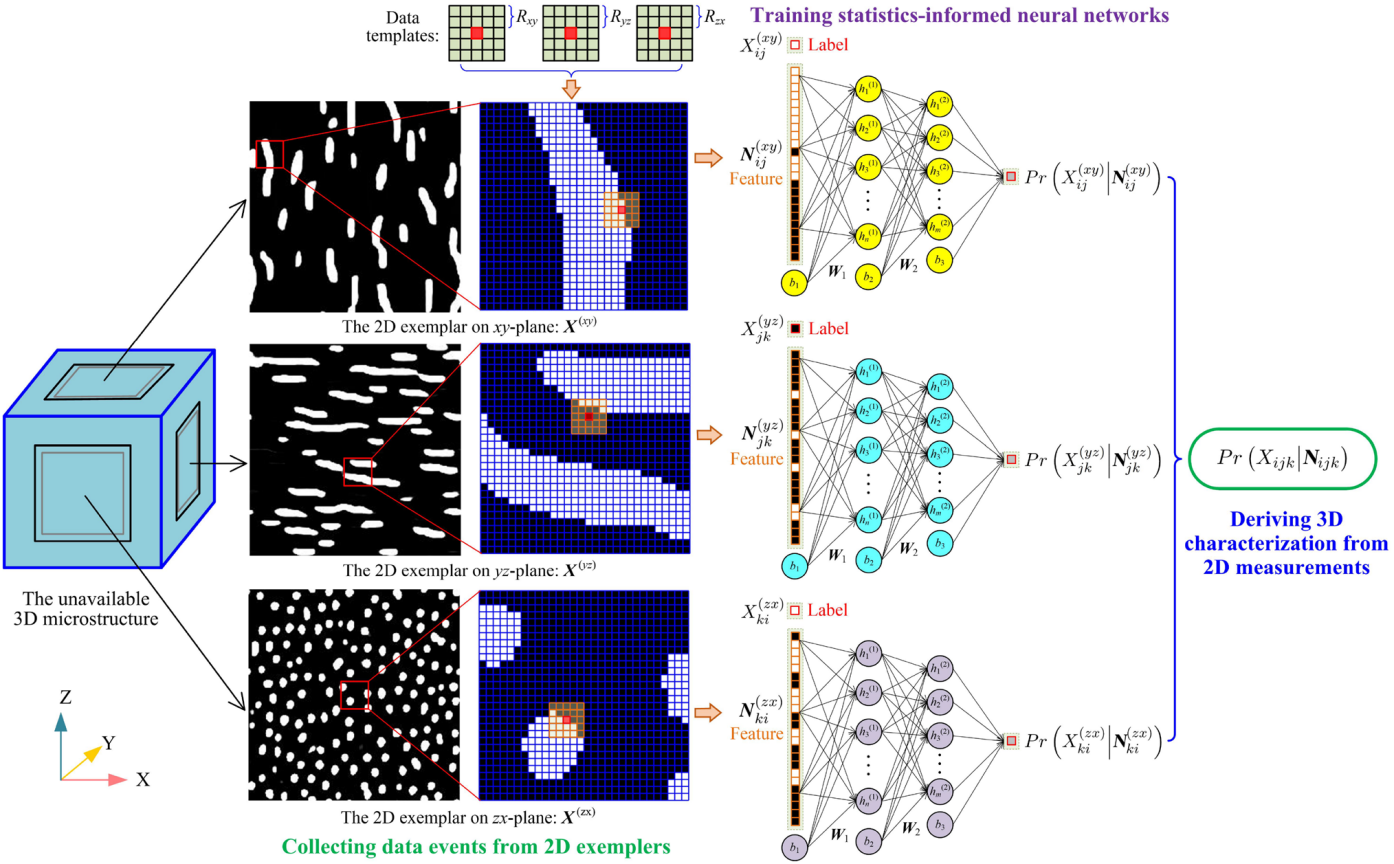
The framework of microstructural characterization using statistics-informed neural network (SINN) [82]

Moreover, a new SINN-based method for 2D-to-3D microstructure reconstruction was also developed by Fu et al. [77], where the morphological statistics on the 2D slice(s) are integrated into FNN models. The workflow of stochastic characterization of random microstructures by training SINN models is graphically explained in Fig 15. To bridge the gap between 2D characterization and 3D reconstruction, a special 3D data template composed of three 2D *L*-shaped data templates on orthogonal planes is designed. And then, the morphological statistics $P\Big (Y_{ijk}\big |{{\boldsymbol{N}}}_{ijk}\Big )$ in 3D space can be derived from the 2D morphological statistics on three perpendicular planes through weighted average:

16 $$\begin{aligned} P\left( Y_{ijk}\Big |{{\boldsymbol{N}}}_{ijk}\right)&=P\left( Y_{ijk}\Big |{{\boldsymbol{N}}}_{ijk}^{(xy)}\cup {{\boldsymbol{N}}}_{ijk}^{(yz)}\cup {{\boldsymbol{N}}}_{ijk}^{(zx)}\right) \\&\approx \dfrac{1}{3}\cdot \bigg [P\left( Y_{ijk}\Big |{{\boldsymbol{N}}}_{ijk}^{(xy)}\right) +P\left( Y_{ijk}\Big |{{\boldsymbol{N}}}_{ijk}^{(yz)}\right) +P\left( Y_{ijk}\Big |{{\boldsymbol{N}}}_{ijk}^{(zx)}\right) \bigg ]\,, \end{aligned}$$

where ${{\boldsymbol{N}}}_{ijk}^{(xy)}$, ${{\boldsymbol{N}}}_{ijk}^{(yz)}$ and ${{\boldsymbol{N}}}_{ijk}^{(zx)}$ are the neighboring voxels on *xy*-, *yz*- and *zx*-planes, respectively. With the 3D morphological statistics $P\Big (Y_{ijk}\big |{{\boldsymbol{N}}}_{ijk}\Big )$, new statistically equivalent 3D microstructures can be synthesized by sequentially generating voxel values from probability sampling.

**Fig. 16:**
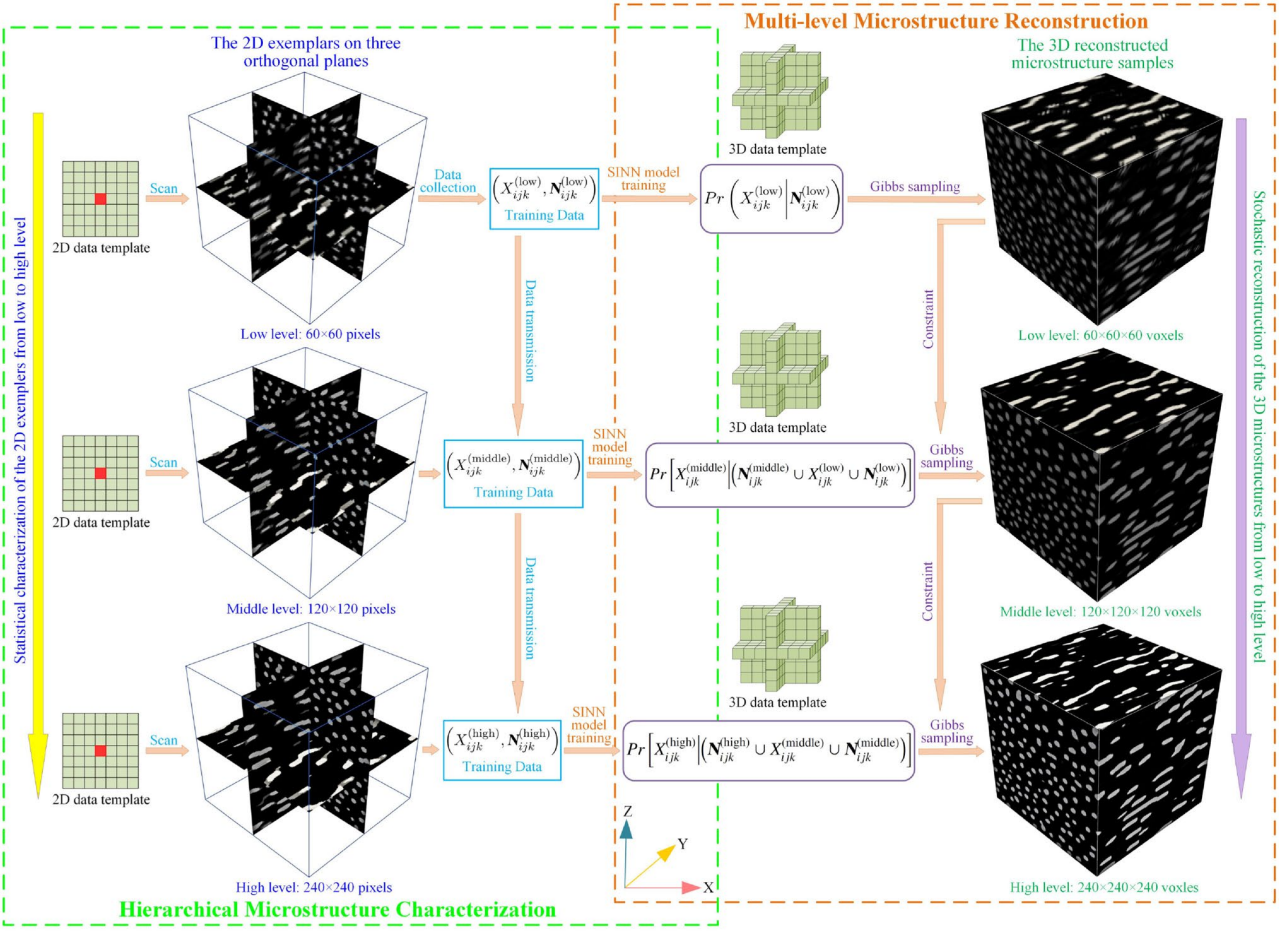
Graphic illustration of the hierarchical SINN-based approach for reconstructing 3D microstructures from 2D slices [82]

Stochastic reconstruction of porous microstructures must account for long-range connectivity and large-scale correlations, as transport properties are primarily governed by the global percolation characteristics of the pore network. To address this challenge, Fu et al. [82, 192] developed hierarchical SINN-based reconstruction approaches, specifically designed to generate well-connected porous microstructures with improved transport properties. As illustrated in Fig 16, these hierarchical methods employ a multi-level statistical learning framework. Morphological statistics are characterized at different scales by training a series of SINN models, enabling the reconstruction process to progressively capture and preserve local, regional, and global microstructural metrics. This structured approach ensures that the synthesized porous microstructures accurately replicate the morphological features and transport behavior of real materials.

The effectiveness of SINN-based hierarchical reconstruction has been rigorously validated, demonstrating statistical equivalence between the generated and real microstructures in terms of both geometric descriptors and transport properties. However, it is important to note that these methods may be less effective for highly heterogeneous porous media, as they rely on a stationary MRF assumption to model internal structures. In cases where extreme heterogeneity exists, additional adaptations or alternative modeling approaches may be required to achieve optimal reconstruction fidelity.

### Convolutional Neural Network-Based Reconstruction

**Fig. 17:**
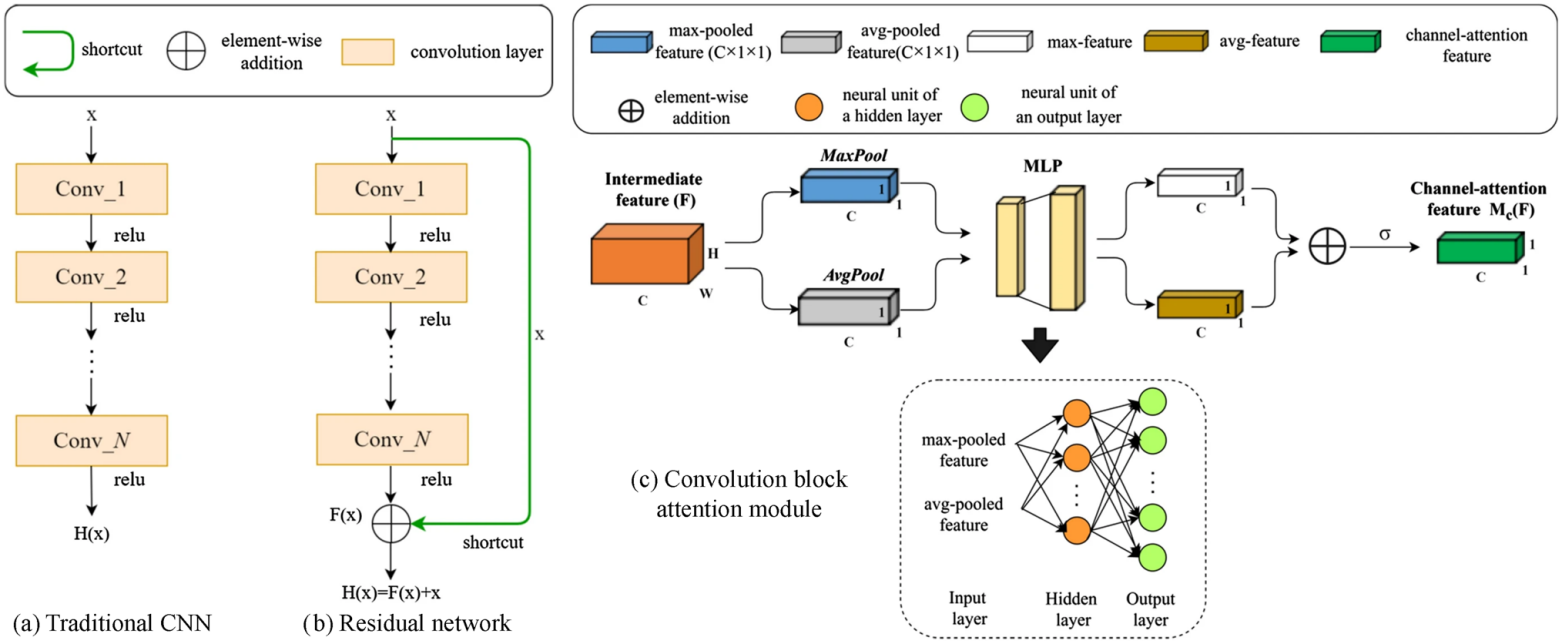
The architectures of the deep residual deconvolution network (DRDN) and convolution block attention module(CBAM) [199]

Convolutional neural networks (CNNs) [281] are particularly well-suited for image-based tasks due to their ability to learn spatial hierarchies of features through convolutional filters and pooling layers. These models process input images in a localized and hierarchical manner, enabling automatic extraction of meaningful patterns such as edges, textures, and shapes. CNNs have been widely adopted in computer vision and have laid the groundwork for microstructure reconstruction, because of their strong capacity of capturing complex morphological features in a data-driven and often feature-free fashion. Generally, the learning ability of CNN for complicated features heavily depends on the number of hidden layers. However, when the number of hidden layers increases to a certain amount, the accuracy of a CNN model will be saturated at first and drop sharply later. To quantitatively characterize and effectively learn the important microstructural features of random porous media via CNN, Zhang et al. [198, 199] adopted the deep residual deconvolution network (DRDN) and convolution block attention module (CBAM) to overcome the degradation problem of CNN. As shown in Fig 17a, b, the architectures of the traditional CNN and the developed DRDN are graphically illustrated. Compared to the traditional CNN, a shortcut connection is added in the DRDN to make the output of convolution layers equal to the original input. This shortcut connection is of great importance to avoid model degradation and improve training stability, based on which identity mapping can be achieved.

As shown in Fig 17c, a CBAM model consists of a channel attention module and a spatial attention module, and it learns"what"through the former and"where"(spatial information) through the latter. Inputting an intermediate feature ${\textbf {F}}\in \mathbb {R}^{C \times H \times W}$ (where *C* for channel, *H* for height and *W* for width) into a CBAM model can yield a 1D channel attention feature and a 2D spatial attention feature, and the entire process can be mathematically expressed as follows:

17 $$\begin{aligned} \left\{ \begin{aligned}&{\textbf {F}}^{\prime } = M_{\textrm{c}}\big ({\textbf {F}}\big ) \otimes {\textbf {F}} \,,\\&{\textbf {F}}^{\prime \prime } = M_{\textrm{s}}\big ({\textbf {F}}^{\prime }\big ) \otimes {\textbf {F}}^{\prime }\,, \end{aligned}\right. \end{aligned}$$

where $\otimes$ denotes element-wise multiplication, $M_{\textrm{c}}(\cdot )$ represents the function of the channel attention module, $M_{\textrm{c}}(\cdot )$ represents the function of the spatial attention module. The channel-attention feature $M_{\textrm{c}}({\textbf {F}})$ and the spatial channel-attention feature $M_{\textrm{s}}({\textbf {F}}^{\prime })$ can be computed via the following equations:

18 $$\begin{aligned} \left\{ \begin{aligned} M_{\textrm{c}}\big ({\textbf {F}}\big )&= \sigma \bigg \{MLP\Big [AvgPool\big ({\textbf {F}}\big )\Big ] + MLP\Big [MaxPool\big ({\textbf {F}}\big )\Big ]\bigg \}\,,\\ M_{\textrm{s}}\big ({\textbf {F}}^{\prime }\big )&= \sigma \bigg \{f^{7\times 7}\Big [AvgPool({\textbf {F}}^{\prime }\big )\Big ] + MaxPool\big ({\textbf {F}}^{\prime }\big )\bigg \}\,,\\ \end{aligned} \right. \end{aligned}$$

where $\sigma (\cdot )$ represents the sigmoid activation function, $f^{7\times 7}(\cdot )$ represents the convolution operation using a kernel of $7 \times 7$, *MLP* denotes the operation of multi-layer perception, $MaxPool(\cdot )$ denotes the maximum pooling operation, and $MaxPool(\cdot )$ denotes the average pooling operation.

**Fig. 18:**
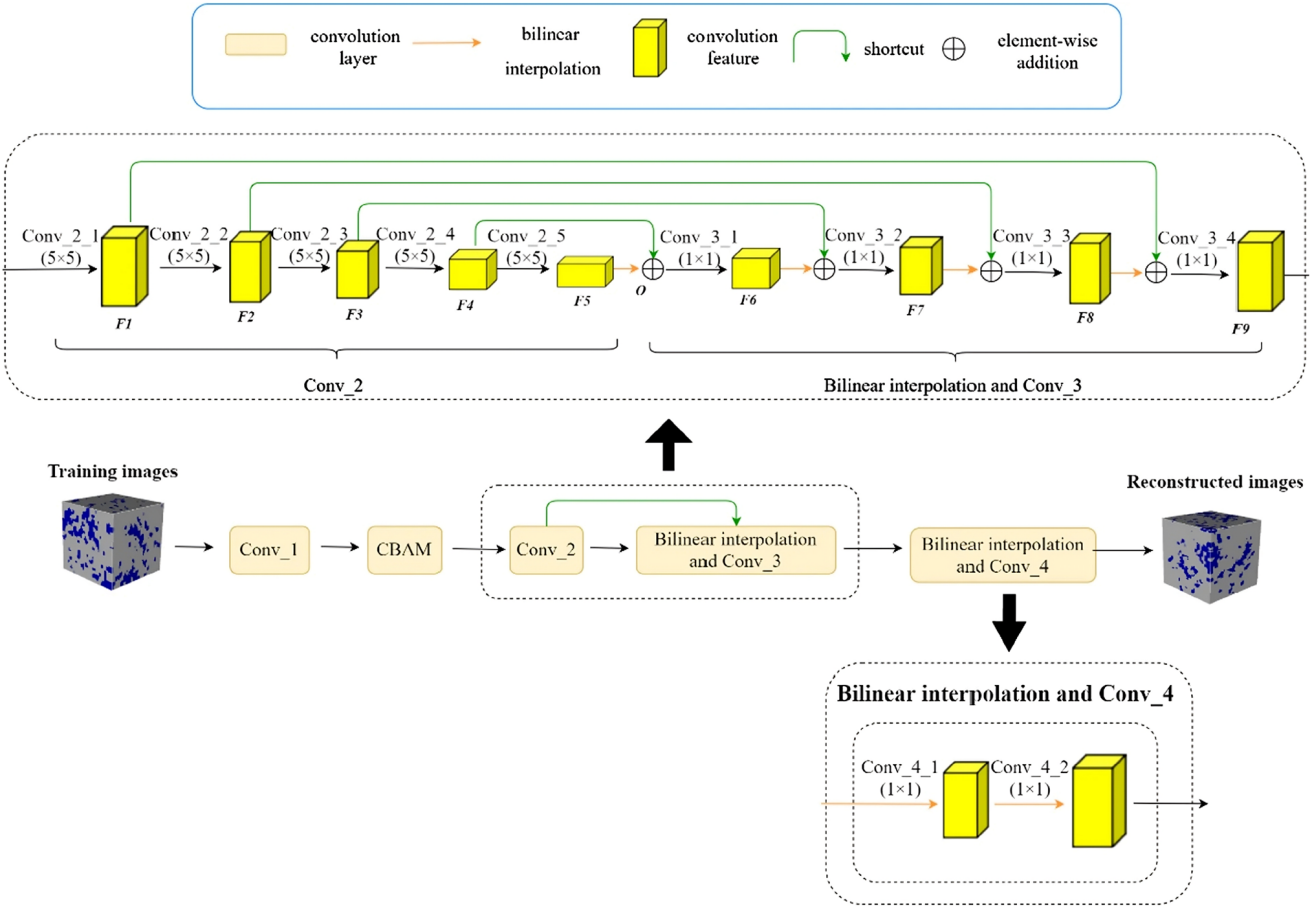
The workflow of microstructural characterization and stochastic reconstruction using the CNN-based method [199]

The workflow of this new CNN-based method [198, 199] in microstructure characterization and stochastic reconstruction is graphically explained in Fig 18. The network model extracts microstructural features from the 3D training images of porous media, and newly reconstructed images of the same size can be outputted from it. The least-square error ($L_2$) loss function is used to minimize the discrepancy between the training and generated images, in which network parameters are iteratively updated until the loss function reaches the convergence conditions. This new CNN-based method exhibits better performance than the direct sampling and traditional CNN-based methods, in aspects of computational efficiency and reconstruction accuracy. In addition, a new convolutional autoencoder-based method was developed by Niri et al. [200] for stochastic characterization and reconstruction of electrode microstructures, and Jung et al. [201] combined convolutional autoencoder with Bayesian optimization to generate dual-phase microstructures with martensite fraction.

### Convolutional Deep Belief Network-Based Reconstruction

**Fig. 19:**
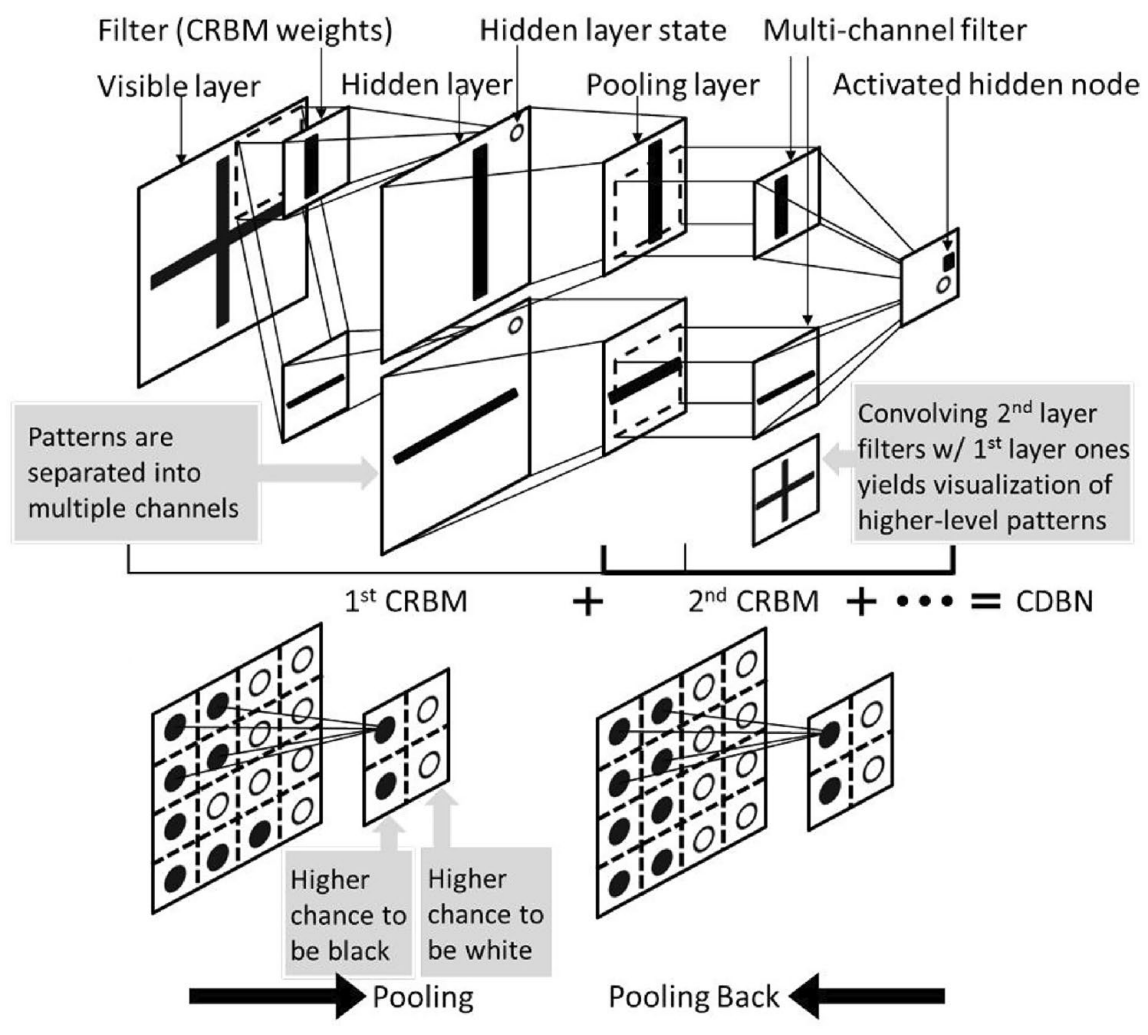
Graphic illustration of microstructural feature extraction using the convolutional deep belief network [102]

Convolutional deep belief networks (CDBNs) have been employed for stochastic characterization and microstructure reconstruction of heterogeneous materials, as demonstrated by Cang et al. [102, 202]. This approach extracts and represents microstructural features at multiple length scales, enabling the capture of large-scale morphological characteristics and long-distance correlations. As illustrated in Fig 19, a CDBN model consists of multiple stacked layers of convolutional restricted Boltzmann machines (CRBMs) and fully connected restricted Boltzmann machines (RBMs). The CRBM layers progressively extract hierarchical spatial features from the input data, while the RBM layers refine and encode these features into a compact latent representation. This structured feature-learning process allows the trained CDBN to effectively map high-dimensional input images (3D digital microstructures) to low-dimensional latent variables, providing a powerful framework for quantitative microstructural characterization.

CDBN functions as a generative model, enabling the random generation of microstructure samples within the learned latent feature space. The process involves randomly assigning binary values to the final layer of the trained CDBN model, followed by an inverse sampling procedure through a deconvolution process to reconstruct new microstructures. Studies have demonstrated that the CDBN-based method can effectively generate virtual microstructures that closely resemble real ones across various morphologies. Moreover, it has been observed that the mean critical fracture strength of the reconstructed microstructures is comparable to reference values, indicating that mechanical integrity is reasonably preserved. However, the current version of the CDBN-based method can only be used for 2D-to-2D microstructure reconstruction, and the reconstruction accuracy can not be guaranteed because key parameters such as threshold are often determined empirically. Future improvements should focus on extending CDBN-based methods to equidimensional reconstructions (2D-to-3D and 3D-to-3D) and optimizing parameter selection strategies through adaptive learning techniques to enhance reconstruction precision.

### Transfer Learning-Based Reconstruction

**Fig. 20:**
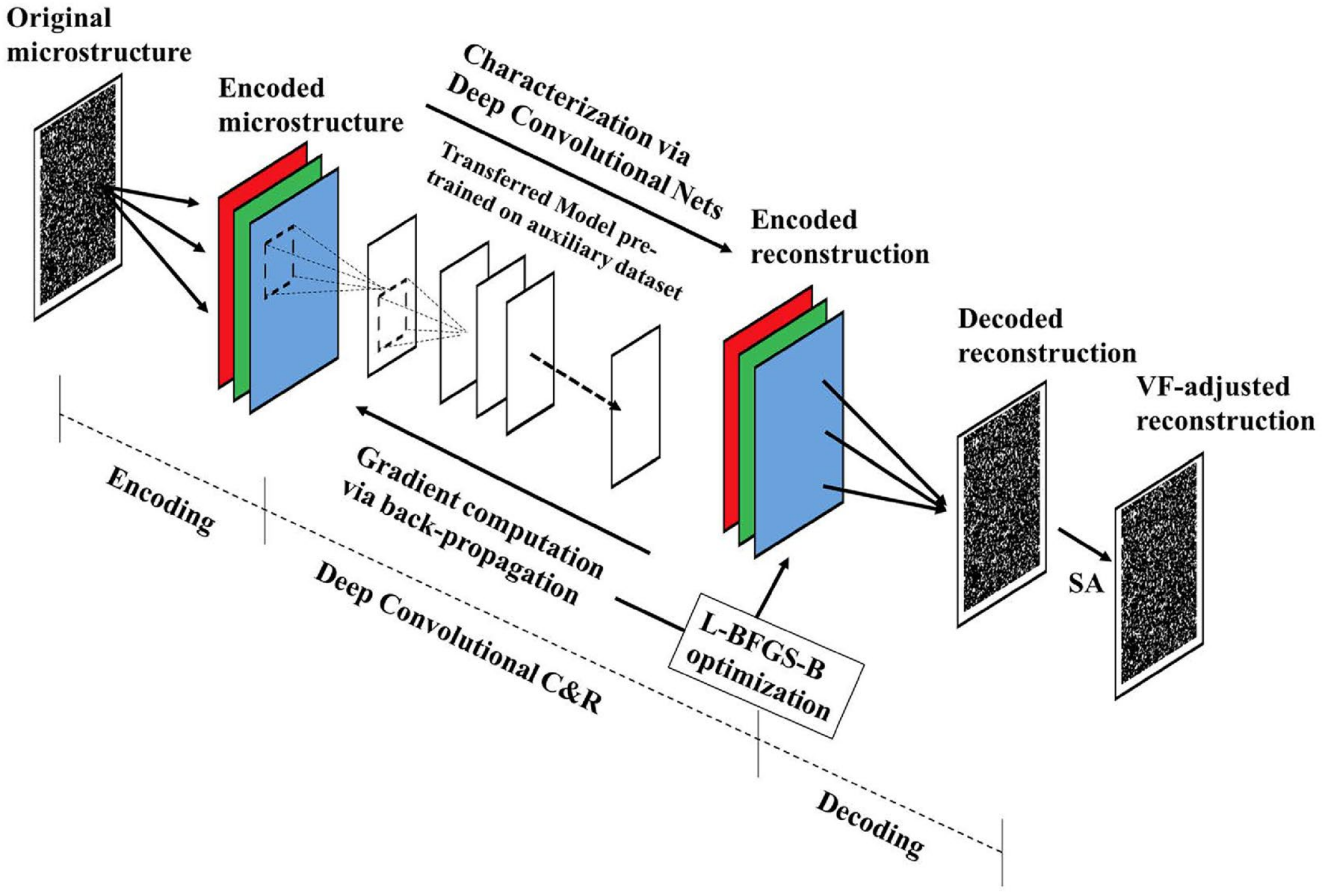
The framework of microstructural characterization and stochastic reconstruction using the transfer learning-based approach [203]

Transfer learning (TL) [282] avoids the training process by reusing the pre-trained deep/machine learning models on new problems. Transferring information that has been learned from previous tasks to new tasks has the potential to improve computational efficiency and generalization significantly. Li et al. [203] proposed a framework based on VGG19 (a pre-trained deep CNN model using ImageNet) for stochastic characterization and microstructure reconstruction, and its workflow is graphically shown in Fig 20. In this method, microstructure reconstruction is cast as an optimization problem to minimize loss function that measures the difference between the characterized information obtained from the exemplars and the reconstructed microstructures. An encoder is added before the VGG19 model to obtain the 3-channel representations of the original image (the digital microstructure) so that the dimensionality of the input data can fit the requirements of the VGG19 model. And a decoder follows the VGG19 model to recover reconstructed microstructures from the latent features.

A Gram-matrix which is used for measuring the difference between image textures is integrated into each layer of the deep CNN to quantify the statistical equivalence between the original and the reconstructed microstructures. The gram-matrixes in the *i*th layer corresponding to the original and reconstructed microstructures are defined as the sum of the inner product between the feature map at the *p*th and *q*th filter at all positions:

19 $$\begin{aligned} \left\{ \begin{aligned} G_{pq}^i = \sum _{r} F_{pr}^i F_{qr}^i\,,\\ \widetilde{G}_{pq}^i = \sum _{r} \widetilde{F}_{pr}^i \widetilde{F}_{qr}^i\,, \end{aligned} \right. \end{aligned}$$

The total loss is the sum of loss in all layers, which is defined as:

20 $$\begin{aligned} \mathcal {L} = \sum _{i} E_i = \frac{1}{4 N_i^2 M_i^2} \sum _{p,q} \left( G_{pq}^i - \widetilde{G}_{pq}^i \right) ^2\,, \end{aligned}$$

where $N_i$ is the number of filters, $M_i$ is the size of the vectorized feature in *i*th layer, $F_{pr}^i$ and $\widetilde{F}_{pr}^i$ are the activation functions of the *p*th filter at position *r* corresponding to the original and reconstructed microstructures respectively. And the gradient $\frac{\partial \mathcal {L}}{\partial x}$ of the loss function can be decomposed by the chain rule:

21 $$\begin{aligned} \frac{\partial \mathcal {L}}{\partial x} = \frac{\partial \mathcal {L}}{\partial E} \cdot \frac{\partial E}{\partial \widetilde{F}} \cdot \frac{\partial \widetilde{F}}{\partial x}\,, \end{aligned}$$

The gradient of the Gram-matrix with respect to the reconstructed image pixel is calculated via back-propagation, and it is then fed into nonlinear optimization to update the image reconstruction result. The above operations are carried out interactively until convergence is reached. This transfer learning-based method [203] shows excellent performance in generating visually similar and statistically equivalent microstructures with different morphologies, but it is only applicable for 2D-to-2D microstructure reconstruction.

**Fig. 21:**
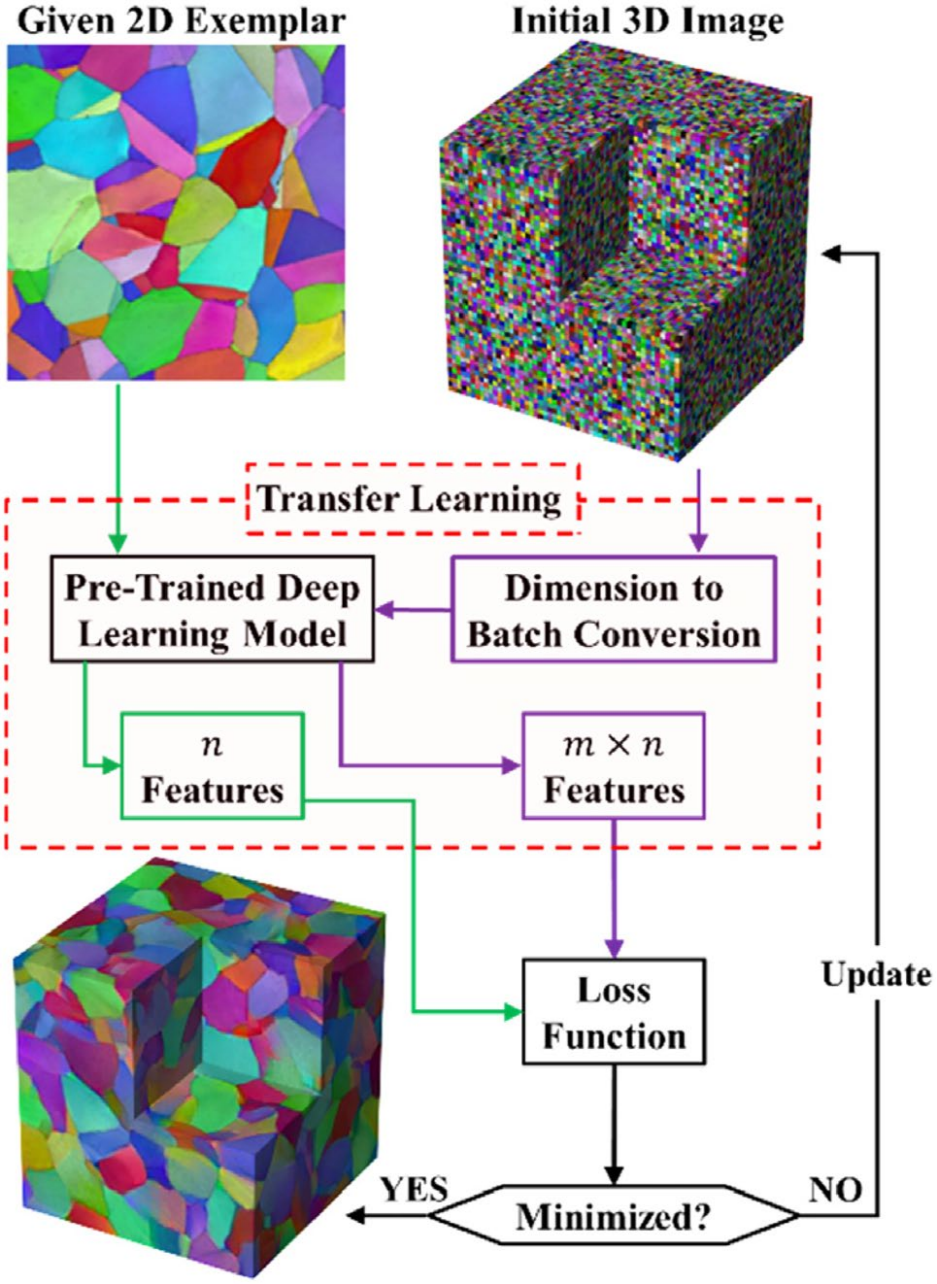
The workflow of 2D-to-3D microstructure reconstruction via transfer learning [105]

Bostanabad et al. [105] developed another transfer learning-based method for 2D-to-3D microstructure reconstruction, where the pretrained VGG19 network with a permutation operator was also utilized. The workflow of 2D-to-3D microstructure reconstruction via transfer learning is graphically illustrated in Fig 21. By adding a permutation operator before the first convolution layer of the VGG19 model, this 2D pre-trained deep CNN model becomes able to accept a 4D tensor of size $b\times h\times w\times c$ (where *b* is the batch size, *h* is the image height, *w* is the image width, and *c* is the number of channels). The permutation operator simultaneously considers 2D cross-sections along three orthogonal directions and converts them to batch from VGG19’s perspective, through which the underlying 3D feature map can be approximated from dependent 2D feature maps. The 3D microstructure of size $s^3$ can be transferred to a total of $3 \times s \times n$ response maps, where *n* is the number of filters. The loss function for RBG images is defined as the sum of Gram-matrixes, given by:

22 $$\begin{aligned} \mathcal {L}\big (\mathcal {X}\big )=\sum _{b=1}^{5} \sum _{l=1}^{l_b} \sum _{d=1}^{3\times s_{b}} \sum _{ij} \left( C_{ij}^{dlb} - G_{ij}^{lb}\right) ^2+\lambda p\big (\mathcal {X}\big )\,, \end{aligned}$$

where $\mathcal {X}$ denotes the RGB triplets in a 3D microstructure, and the optimal $\mathcal {X}$ can be obtained by minimizing the loss function. This transfer learning-based method has been validated by a series of microstructures with distinct morphologies, and the results demonstrate that it has good reconstruction accuracy and computational efficiency.

In addition, Bhaduri et al. [205] further developed the transfer-learning method that was originally proposed by Li et al. [203] to be an optimization-based reconstruction procedure, where an overall loss function that consists of Gram matrix loss, total variation loss and two-point probability loss is integrated into the transfer-learning model. Lubbers et al. [204] applied VGG-19 to infer the low-dimensional microstructure representation and synthesize morphologically similar images. Du et al. [206] developed a deep transfer learning method to reconstruct 3D porous microstructure from 3D training images. Generally, these transfer learning-based methods are able to generate morphologically similar microstructures based on a single given target microstructure from arbitrary material systems, but the pre-trained deep/machine learning models are often used as a"black box", which makes it difficult to understand the relationships between the network architectures and the microstructure characteristics.

### Variational Autoencoder-Based Reconstruction

**Fig. 22:**
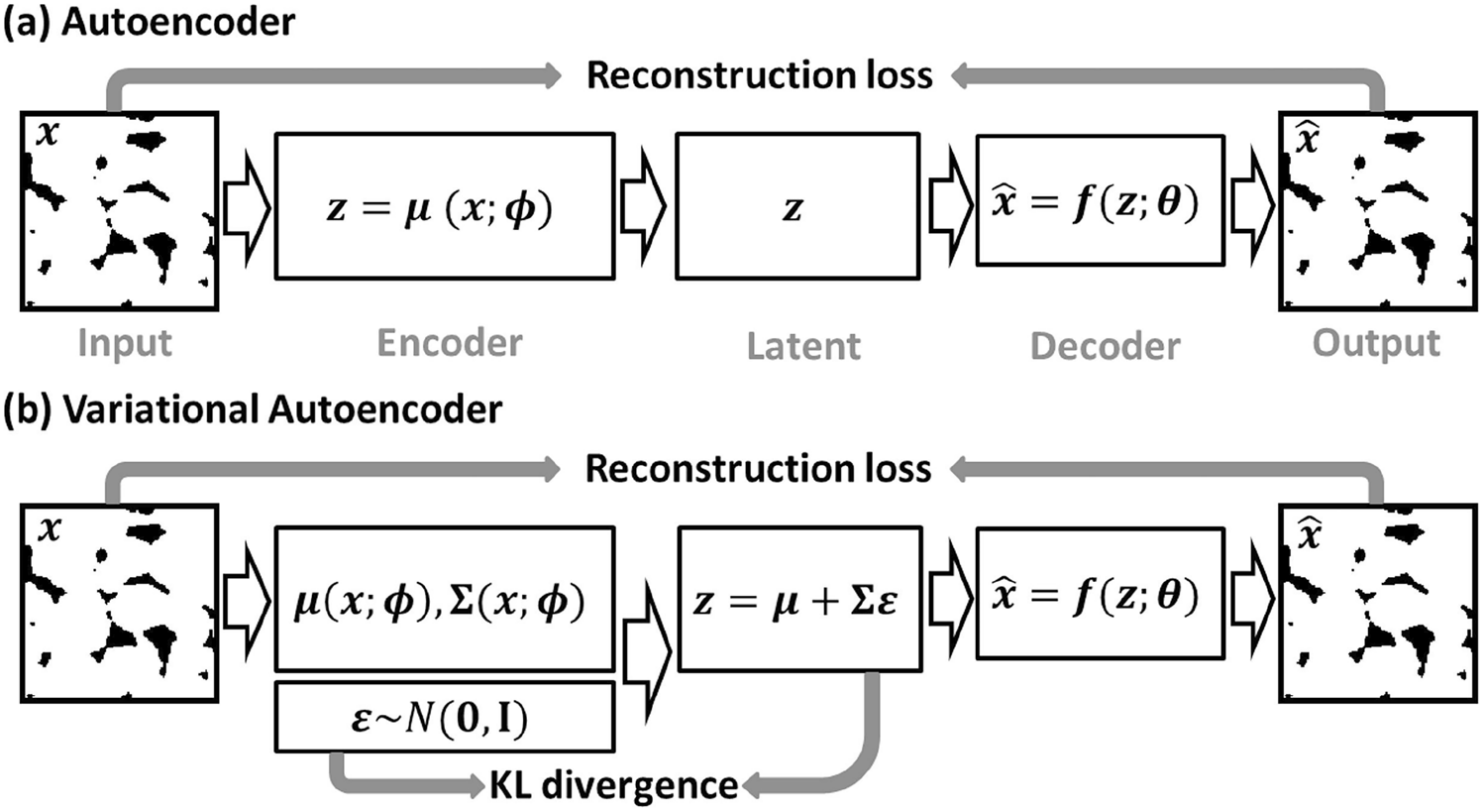
The differences between (**a**) a standard autoencoder and (**b**) a variational autoencoder for microstructure reconstruction [208]

As an extension of the standard autoencoder (AE), the variational autoencoder (VAE) [283] is a relatively new type of probabilistic generative network in deep learning. Both AE and VAE contain two components: encoder and decoder, as illustrated in Fig 22. The encoder maps the input variable *x* to a feature vector $z=f(x)$ in the latent space, and the decoder recovers the input $\hat{x}=g(z)$ from the latent space. However, standard AE lacks an explicit probabilistic framework, meaning the learned latent space may not align with the true data distribution. Consequently, when new samples are generated by randomly sampling from this latent space, the outputs may appear structurally valid but lack statistical consistency with the original dataset.

To address the above mentioned problem, the distribution of latent features *z* is used in VAE, where the original input is compressed into a probability distribution in the latent space, instead of a fixed data point. There are two probability density distributions in VAE. The encoder’s distribution $q_{\varphi }(z\,\vert \, x)$ is used to infer the latent variables from the input data, while the decoder’s distribution $q_{\phi }(\hat{x}\,\vert \, z)$ reconstructs the input by approximating the probability distribution of original data, according to the probability distribution of latent variables. The loss function $\mathcal {L}(\theta , \varphi ; X)$ of a VAE model is defined as follows:

23 $${\mathcal{L}}(\theta ,\varphi ;X) = {\mathbb{E}}_{{q_{\varphi } (z|x)}} \{ \log _{2} \left[ {q_{\phi } (\hat{x}|z)} \right]\}  - D_{{{\mathrm{KL}}}} \left[ {q_{\varphi } (z|x)\left\| {q_{\phi } (\hat{x}|z)} \right.} \right],$$

The first term on the right-hand side measures the difference between the input *x* and the reconstruction $\hat{x}$; and the second term denotes the Kullback-Leibler divergence between the encoded latent distribution $q_{\varphi }(z\,\vert \, x)$ and the posterior $q_{\phi }(\hat{x}\,\vert \, z)$. This loss function under constrained conditions can be minimized via stochastic gradient descent.

**Fig. 23:**
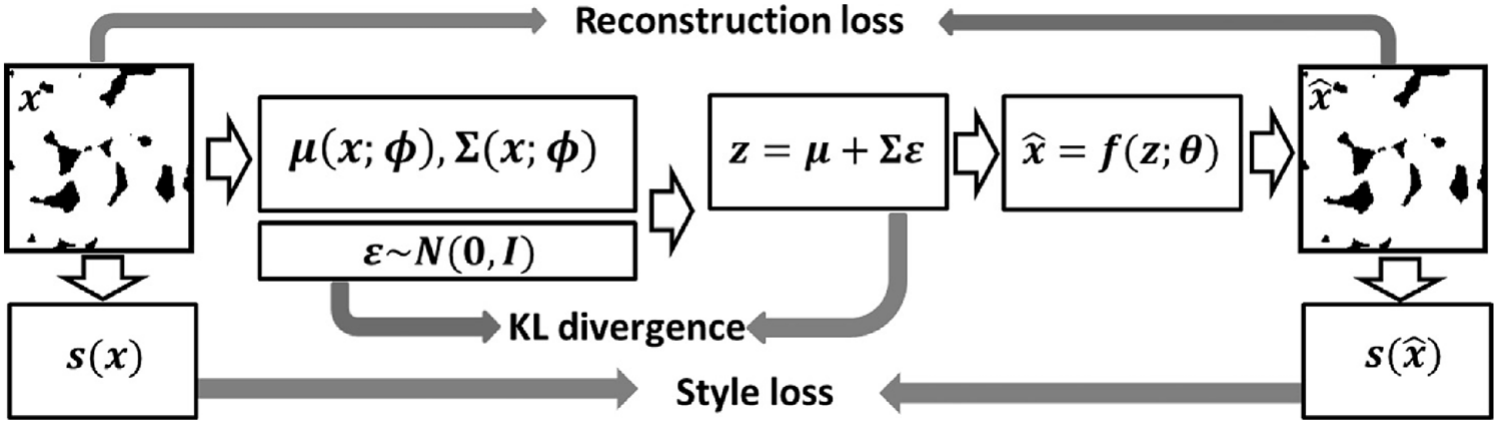
The workflow of the VAE-based method for microstructure characterization and reconstruction [208]

Cang et al. [208] proposed a VAE-based method for microstructure characterization and reconstruction. In this method, both the decoder and the encoder are composed of feed-forward CNNs to extract and generate local morphological patterns respectively, and they are jointly trained to minimize the discrepancy between the generated and real microstructures. The schematic of the proposed morphology-aware VAE is shown in Fig 23. This generative model consists of a VAE for image encoding and decoding and a VGG net for computing the style vector *G*. Specifically, the encoder compresses the dimension of the input image from $128\times 128$ to latent vector $16\times 1$, and the decoder is symmetric to the encoder. A pretrained VGG net is used to generate a style vector that consists of variance-covariance matrices of the hidden state of the input images. The output image is created by minimizing the difference between the generated style vector and the referenced style vector. A style penalty $\mathcal {L}_{\textrm{ST}}$ and the model collapse loss $\mathcal {L}_{\textrm{MC}}$ are added to the total loss function of VAE, given by:

24 $$\begin{aligned} \mathcal {L}\big (\theta , \varphi ; X\big ) = \mathcal {L}_{\textrm{reconstruction}}+\mathcal {L}_{\textrm{KT}}+\mathcal {L}_{\textrm{ST}}+\mathcal {L}_{\textrm{MC}}\,, \end{aligned}$$

This VAE-based method has been validated by analyzing the porous microstructures of sandstones, and it exhibits a much better performance and higher data efficiency in constructing reliable structure-property mappings, compared with the state-of-the-art Markov random field method.

Du et al. [211] and Zhang et al. [212] developed a Fisher information-based VAE for microstructure characterization and reconstruction. Fisher information is an important tool for describing information behavior in information systems, which is introduced here to measure how much valuable information is obtained from the sample data to estimate the unknown parameters in the distribution. The loss function of the Fisher information-based VAE is defined as follows:

25 $$\begin{aligned} \mathcal {L}\big (\theta , \varphi ; X\big ) = \mathcal {L}_{\textrm{reconstruction}}+\mathcal {L}_{\textrm{KT}}-\lambda _z \Big \vert tr\Big (J\big (z\big )\Big )-F_z\Big \vert - \lambda _{\hat{x}} \Big \vert tr\Big (J\big (\hat{x}\big )\Big )-F_{\hat{x}}\Big \vert \,, \end{aligned}$$

where $\big \vert tr\left( J(z)\right) -F_z\big \vert$ controls the Fisher information of the encoder; $\big \vert tr\left( J(\hat{x})\right) -F_{\hat{x}}\big \vert$ controls the Fisher information of the decoder; $\lambda _z$ and $\lambda _{\hat{x}}$ are the adjustment coefficients. This method shows excellent performance in microstructural characterization, and the reconstructed microstructures can accurately preserve the morphological features (such as variogram, multiple-point connectivity and porosity) and transport properties (such as permeability) of the real microstructure. In the later work, Zhang et al. [213] further developed a new VAE-based method (SE-FBN-VAE) for microstructure reconstruction of random porous media, where the squeeze and excitation network (SENet) and the fixed batch normalization (FBN) are combined with VAE. The SENet is used to enhance the feature extraction performance, and the FBN is used for data normalization in neural networks. In addition, Laloy et al. [207], Chan et al. [209] and Kamrava et al. [210] also applied VAE for microstructure characterization and 2D-to-2D microstructure reconstruction.

VAEs prioritize learning a smooth, compact latent space that captures the global statistical properties of the training data. As a result, the reconstructed microstructures tend to closely resemble the original samples, limiting the model’s ability to generate diverse or novel structures beyond the observed variations. Besides, VAE-based reconstructions may struggle with sharp details and long-range dependencies in complex microstructures, as the latent space often favors smoother transitions over fine-grained spatial features. Overcoming this limitation requires either: Enhanced latent space representations (e.g., hierarchical VAEs or hybrid models with GANs) and post-processing techniques (e.g., texture blending or conditional sampling) to introduce more variability.

### Generative Adversarial Network-Based Reconstruction

Generative adversarial networks (GANs) have emerged as a powerful tool for stochastic microstructure reconstruction due to their strong ability to learn complex spatial patterns and generate high-fidelity samples. Their primary advantage lies in adversarial training, where a generator continuously improves at creating realistic microstructure samples while a discriminator enhances its ability to distinguish real from fake samples. This dynamic competition enables GANs to capture intricate morphological patterns and long-range correlations more effectively than traditional methods. Additionally, numerous GAN variants have been adapted for stochastic characterization and reconstruction of random microstructures, including Wasserstein GAN [238, 241], conditional GAN [234, 236], BicycleGAN [250], StyleGAN [245], VAE-GAN [268, 269], AE-GAN [271, 272], pix2pix GAN [251], multi-stage GAN [252], and self-attention GAN [249]. As summarized in Table 2, these GAN-based reconstruction approaches have been systematically categorized based on an extensive literature review.

#### Standard GAN-Based Reconstruction

**Fig. 24:**
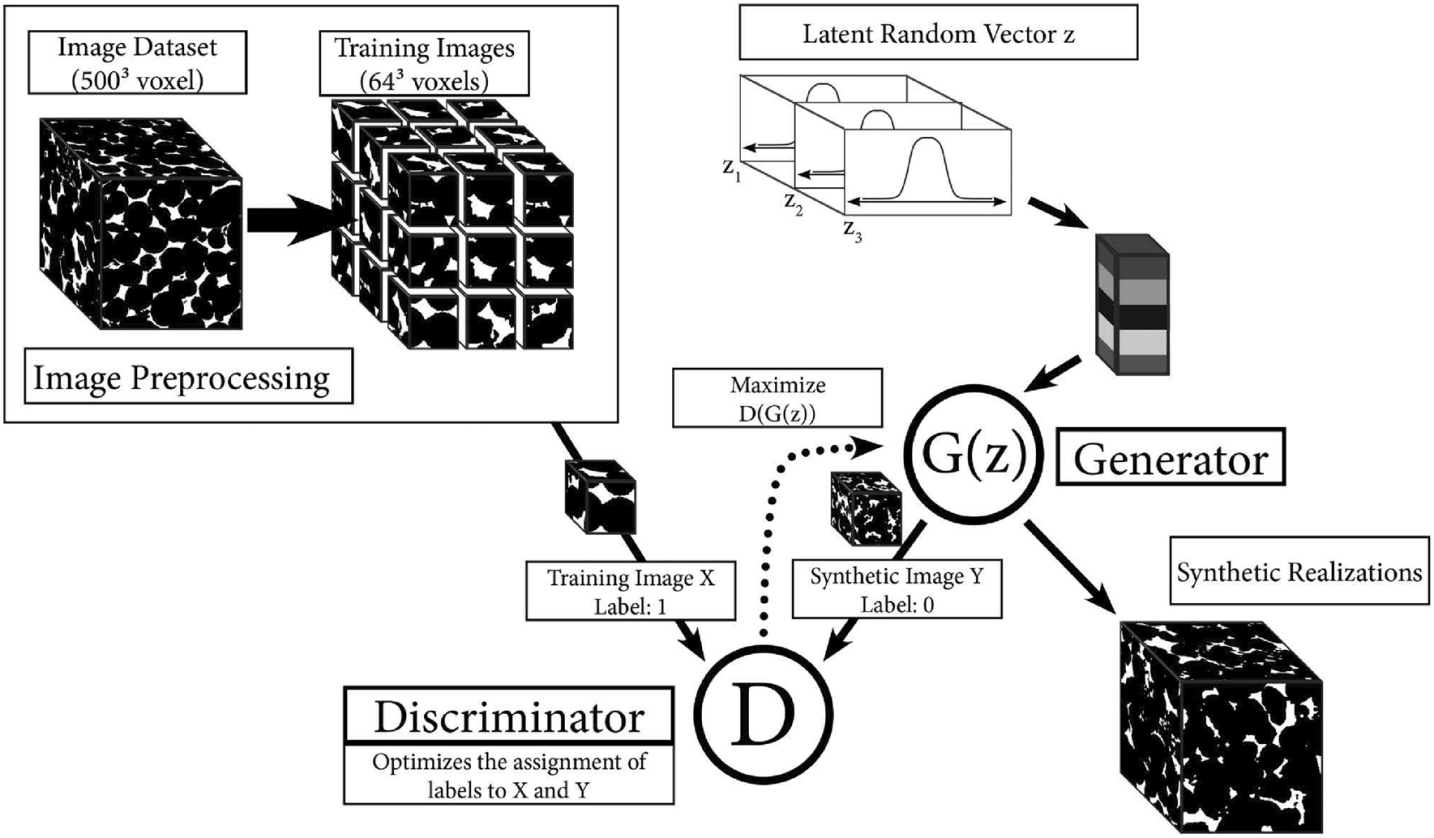
The framework of the standard GAN-based method for stochastic characterization and microstructure reconstruction [103]

A standard GAN model is a set of neural networks that can learn the underlying latent probability distribution from high-dimensional image data. Training GAN models is to statistically characterize the morphology or texture of random porous microstructures, and new microstructure samples can then be randomly generated by honoring the learned statistics. Here, the GAN-based 3D-to-3D (equidimentional) microstructure reconstruction [103, 222] are first introduced and discussed. As graphically illustrated by Fig 24, GAN formulates the learning problem as a two-player zero-sum game between the generator *G* and the discriminator *D*. The generator *G* creates a synthetic realization by mapping random variables from the latent space *Z* into the image space. The discriminator *D* receives both the real samples (label 1) from the training dataset and the"fake"samples $G({\textrm{z}})$ (label 0) created by the generator. The discriminator assesses the probability of a random sample coming from the real data distribution and then assigns a label to each sample. The discriminator tries to label each sample correctly, while the generator tries to"fool"the discriminator by generating morphologically realistic samples.

The training process of a GAN model is to optimally adjust the network’s parameters in an unsupervised manner by solving a minimization-maximization problem [284, 285], which can be mathematically expressed as :

26 $$\begin{aligned} \min \limits _{G} \max \limits _{D}\ \ \mathcal {L}_{\textrm{gan}}\big (G, D\big )&= \mathbb {E}_{{{\boldsymbol{x}}}\sim P_{\textrm{data}}({{\boldsymbol{x}}})} \Big [\log \Big (D({{\boldsymbol{x}}})\Big )\Big ]+\mathbb {E}_{{{\boldsymbol{z}}}\sim P_{{\boldsymbol{z}}}({{\boldsymbol{z}}})}\Big [\log \Big (1-D\big (G({\boldsymbol{z}})\big )\Big )\Big ]\,, \end{aligned}$$

where $P_{\textrm{data}}({\boldsymbol{x}})$ is the probability distribution of real data; $P_{{\boldsymbol{z}}}({\boldsymbol{z}})$ is the probability distribution of the input vector; $D\big (G({\boldsymbol{z}})\big )$ is the probability assessed by the discriminator *D* to judge how possible the fake sample $G({\boldsymbol{z}})$ created by the generator is from the real data set; and $\mathbb {E}$ denotes the mathematical expectation. Once the GAN model is adequately trained, the generator *G* represents a leaned distribution that is very close to the real data distribution, and the discriminator *D* will assign a probability with a value close to 1/2 to each sample, as it can no longer distinguish the real samples from the generated ones. It has been proven that GAN-based characterization is able to capture complicated morphological features of porous media, and the reconstructed microstructure samples are statistically equivalent to the real ones in terms of pore morphology and transport properties such as permeability.

**Fig. 25:**
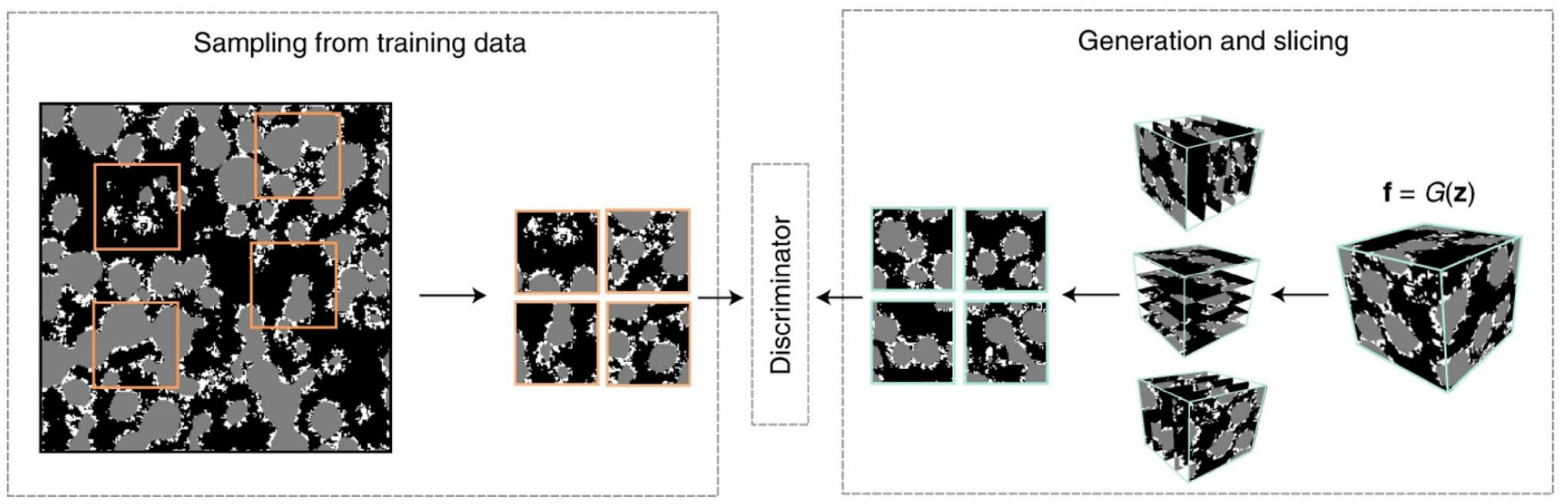
The discriminator assesses the quality of 2D-to-3D reconstructed images via cross-section examining [96]

As to dimensionally-upgraded microstructure reconstruction via GAN [96, 226], the generator *G* needs to create 3D images from 2D slices, and the discriminator *D* needs to distinguish 3D reconstructed images from the 2D real images. Following the same procedure in Fig 24, Valsecchi et al. [225] first tackled this 2D-to-3D reconstruction problem by assessing the quality of reconstructed volumes via cross-section examining, as explained in Fig 25. The generator *G* is designed to produce 3D images while the discriminator *D* works on all 2D cross-sections in the 3D generated volume. Besides, multiple variations have also been developed to further improve the accuracy and efficiency of 2D-to-3D microstructure reconstruction, such as SliceGAN-based method [96], deep convolutional GAN-based method [221], BicycleGAN-based method [233], Wasserstein GAN-based method [239], and StyleGAN-based method [286].

Generally, properly trained GAN models can generate new microstructure samples within minutes and can also be stored for repeated use. However, training volumetric GANs is computationally intensive and time-consuming, requiring significant resources, especially for large datasets with high-resolution 3D images. In terms of reconstruction quality, GAN-based methods [96, 103, 222] have been shown to accurately preserve the statistical characteristics, morphological features, and physical properties of real microstructures. However, the black-box nature of GANs makes them difficult to interpret, posing challenges in ensuring accountability and transparency in microstructural characterization and stochastic reconstruction.

#### Conditional GAN-Based Reconstruction

Although standard GAN models have been successfully applied to reconstruct various types of porous media, the generated samples often exhibit randomness and may lack representativeness. Specifically, standard GAN may struggle to produce a diverse set of samples with user-defined properties. To address this issue, various methods have been proposed to incorporate geometric and/or physical properties, thereby constraining the black-box nature of the reconstruction process.

Conditional GAN (CGAN) [287, 288] represents an improvement over standard GAN by introducing conditional dependencies. In a standard GAN, the model is trained in an unconditional manner, relying solely on the training data to learn the underlying distribution. However, in certain applications, additional conditional information must be explicitly incorporated. In the context of porous media reconstruction, geometric and physical descriptors serve as essential conditional inputs. The architecture of CGAN is similar to that of standard GAN but extends its functionality by integrating conditional data *y* alongside random noise *z* in the generator. Additionally, the conditional information *y* is exposed to the discriminator, influencing the distance between real and generated distributions. An alternative approach involves incorporating conditional information into a separate discriminator and modifying the loss function accordingly. The training process of CGAN models can be mathematically formulated as:

27 $$\begin{aligned} \min \limits _{G} \max \limits _{D}\ \ \mathcal {L}_{\textrm{cgan}}\big (G,D\big )&= \mathbb {E}_{x \sim P_{\textrm{data}}(x)}\Big [\textrm{log}\Big (D(x|y)\Big )\Big ]+\mathbb {E}_{{\boldsymbol{z}}\sim P_{z}({\boldsymbol{z}})} \Big [\log \Big (1-D\big (G(z|y)\big )\Big )\Big ]\,. \end{aligned}$$

**Fig. 26:**
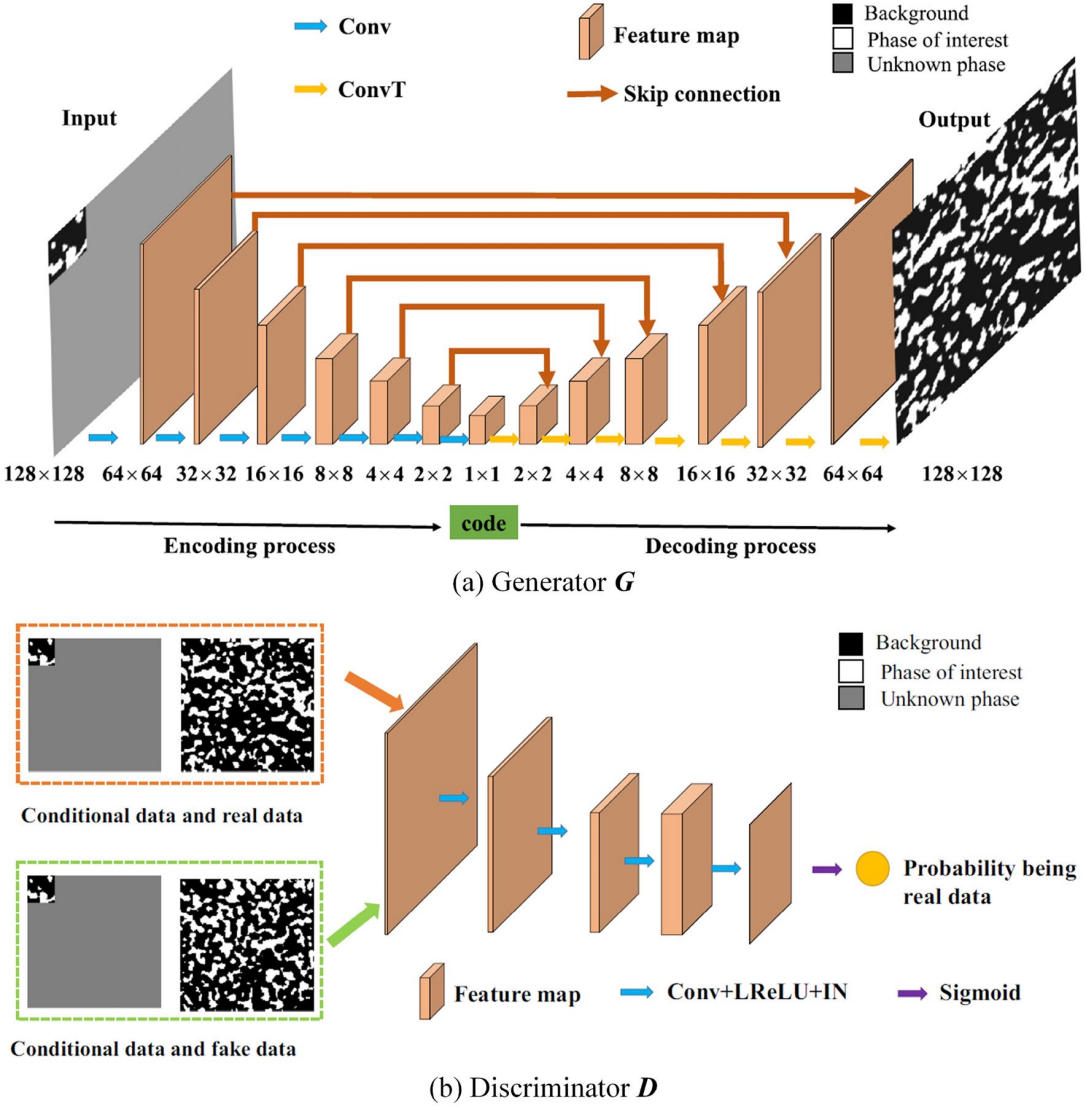
The architectures of a generator ${{\boldsymbol{G}}}$ and a discriminator ${{\boldsymbol{D}}}$ in the CGAN-based reconstruction framework [234]

Besides, another strategy involves incorporating conditional information directly into the loss function to further regulate the training process of CGAN. Feng et al. [233, 234] utilized this method for reconstruction from a small sub-area, allowing the incorporation of user-defined conditional data and an arbitrary number of loss functions, as illustrated in Fig 26. While the discriminator remains the same as in the standard GAN model, the loss functions are modified. In addition to the standard CGAN loss $\mathcal {L}_{\textrm{cgan}}(G,D)$, L1 loss $\mathcal {L}_1$, pattern loss $\mathcal {L}_{\textrm{pattern}}$ and porosity loss $\mathcal {L}_{\textrm{porosity}}$ are introduced. The pattern loss $\mathcal {L}_{\textrm{pattern}}$ measures the discrepancy between predictions and targets based on multi-point configurations, while the porosity loss $\mathcal {L}_{\textrm{porosity}}$ quantifies the deviation in porosity between the generated samples and the training data. The total loss function is formulated as:

28 $$\begin{aligned} \mathcal {L}_{\textrm{total}}&= \mathcal {L}_{\textrm{cgan}}(G,D) + \lambda _1 \mathcal {L}_1 + \lambda _{\textrm{pattern}} \mathcal {L}_{\textrm{pattern}} + \lambda _{\textrm{porosity}} \mathcal {L}_{\textrm{porosity}}\,, \end{aligned}$$

This formulation enhances the control over generated structures by enforcing domain-specific constraints, thereby improving the accuracy and reliability of the reconstructed porous media.

In addition, Yang et al. [286] further developed a hierarchical stacked CGAN-based reconstruction method, capable of generating random microstructures at different resolution levels. Song et al. [289] proposed a CGAN-based method for text-to-image synthesis, which realizes 3D reconstruction of core grayscale images from only pore parameters. This method can randomly generate bi-phase porous microstructure samples with different pore location distributions. Su et al. [235] incorporated porosity as a component of the CGAN loss function, while Chen et al. [231] introduced Minkowski functionals to enhance the loss function. Tang et al. [230] utilized processing parameters within CGAN, enabling regression-based microstructure reconstruction. Furthermore, many researchers [227, 229, 236, 237] have applied CGAN to facilitate dimensionally-upgraded reconstruction of various random microstructures.

#### Wasserstein GAN-Based Reconstruction

Although standard GAN models [96, 103, 222] can generate microstructure samples with repetitive or similar morphologies, determining when the training process reaches maturity remains challenging. The loss function, which measures the probability distance between generated and real samples, does not always ensure stable convergence. This instability can lead to mode collapse, particularly when training on a limited number of images. Mode collapse is a common issue in GAN training, where the generator learns to produce a limited set of outputs (or even identical outputs) regardless of input variation, thereby failing to capture the full diversity of the real data distribution. This leads to reduced variability in generated microstructures and undermines the model’s ability to represent the true morphological complexity of the dataset.

Wasserstein GAN (WGAN) [238, 241, 244] addresses the above issues by incorporating a gradient-penalized loss function, ensuring greater training stability. By integrating the Wasserstein distance under the 1-Lipschitz continuity constraint, WGAN improves optimization and mitigates mode collapse. Since the discriminator *D* in WGAN does not inherently satisfy this constraint, WGAN with gradient penalty (WGAN-GP) introduces an additional term to the loss function:

29 $$\begin{aligned}\\ \min \limits _{G} \max \limits _{D}\ \ \mathcal{L}_{\textrm{wgan}}\big (G,D\big )& = \mathbb {E}_{{\boldsymbol{x}}\sim P_{\textrm{data}}({\boldsymbol{x}})}\Big [\textrm{log}\Big (D({\boldsymbol{x}})\Big )\Big ] + \mathbb {E}_{{\boldsymbol{z}}\sim P_{z}({\boldsymbol{z}})}\Big [\textrm{log}\Big (1-D\big (G({\boldsymbol{z}})\big )\Big )\Big ] +\lambda \underbrace{\mathbb {E}_{\hat{x}\sim P_{\hat{x}}} \left[ \Big (\big \Vert \nabla_{\hat{x}}D(\hat{x})\big \Vert _2 -1\Big )^2\right] }_{\text {gradient penalty}}, \end{aligned}$$

where $\hat{{\boldsymbol{x}}}$ is sampled from the generated images $G({\boldsymbol{z}})$ and the real images *x* with $\eta$ uniformly sampled between 0 and 1, given by

30 $$\begin{aligned} \hat{{\boldsymbol{x}}}= \eta \, G({\boldsymbol{z}})+(1-\eta ){{\boldsymbol{x}}}\,, \end{aligned}$$

Compared to the standard GANs, WGAN and its variants significantly enhance training stability, effectively preventing mode collapse across various experiments.

Iyer et al. [228] proposed an auxiliary classifier WGAN with gradient penalty (ACWGAN-GP) to synthesize microstructures under a given processing condition. This represents the first application of GANs in the characterization and reconstruction of multiphase materials, where the cooling method was incorporated as a conditioning parameter in the GAN model. The auxiliary classifier GAN provides additional side information to both the generator and the discriminator, enhancing the visual quality and diversity of the synthesized images. By incorporating the discriminator gradient with an $L_2$ norm penalty, the quality of the generated microstructures is improved at an accelerated rate. This approach enables tighter control over the generation process and provides deeper insights into the synthesized microstructures by introducing additional processing parameters into the loss functions.

**Fig. 27:**
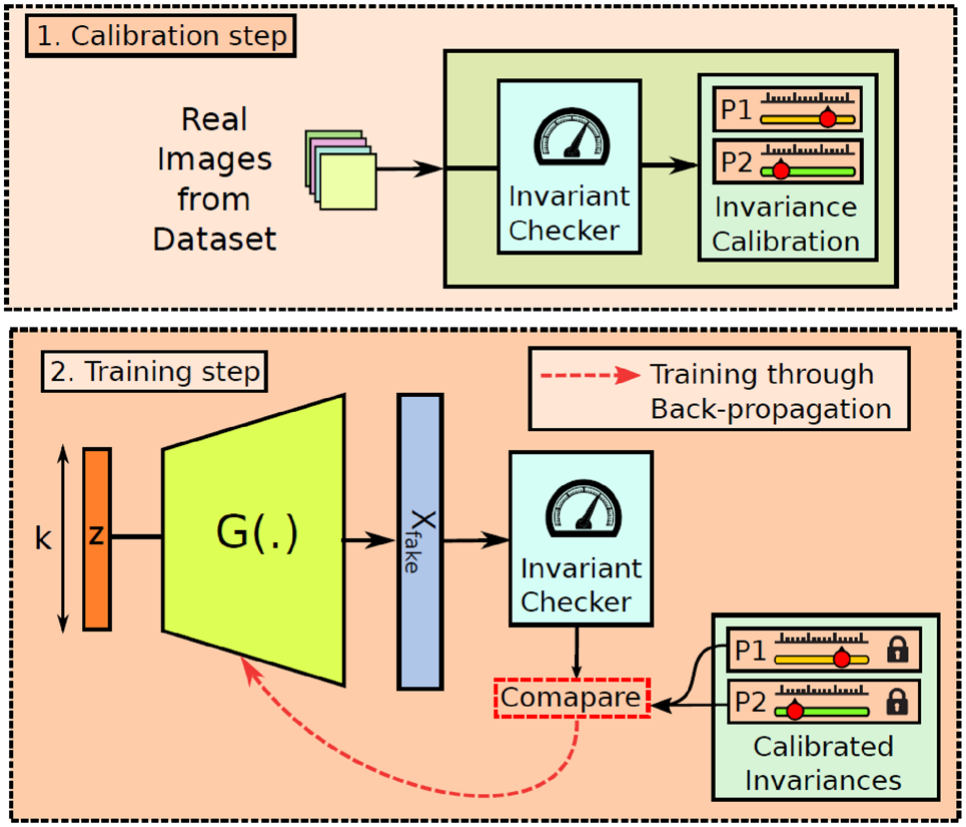
The workflow of generative invariance network network [232]

Singh et al. [232] combined WGAN-GP with a fixed discriminator. In their research, they replaced the traditional trainable discriminator with an Invariance Checker function that verifies whether the generated samples obey known physical invariances. To implement this approach, the Invariance Checker must first be calibrated. Parameters derived from the training data are verified against calibrated threshold parameters, denoted as $p^*_1$ and $p^*_2$. The generator is then trained using a loss function derived from fine-tuned invariance, allowing for fine-grained control over the physical properties of the generated outputs. Their method can be interpreted as a Conditional GAN (CGAN) with a pre-trained and fixed discriminator. They refer to this approach as a *generative invariance network* (GIN), and its architecture is illustrated in Fig 27.

**Fig. 28:**
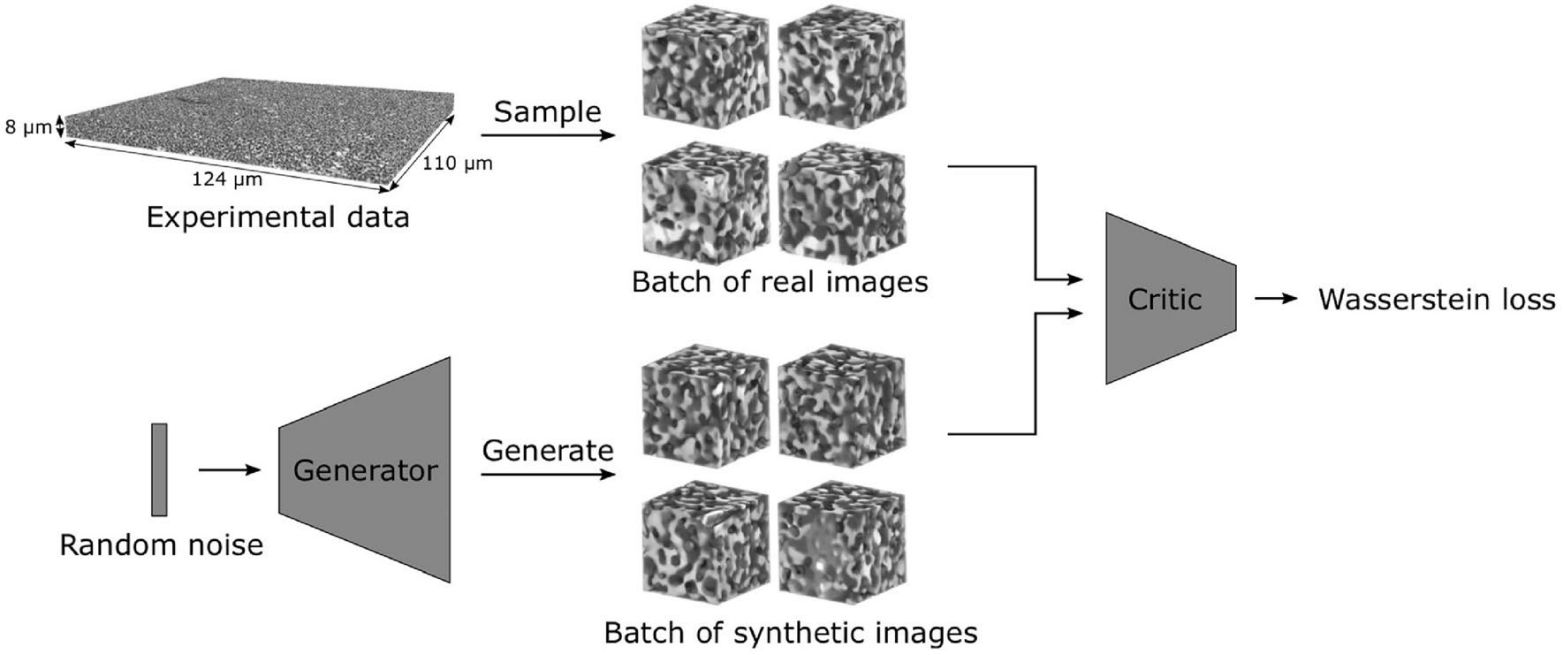
Stochastic microstructure reconstruction via WGAN [239]

As illustrated in Fig 28, Hsu et al. [239] applied WGAN for 3D-to-3D microstructure reconstruction in computational material engineering. During training, batches of subvolumes—randomly sampled from rotated and mirror-flipped experimental 3D microstructures—along with batches of randomly generated 3D images from the generator are fed into the critic network to compute the Wasserstein loss and update gradients. Notably, grayscale 3D images can be directly used as input, and the generated microstructures demonstrate excellent performance in preserving microstructural metrics and electrochemical properties.

**Fig. 29:**
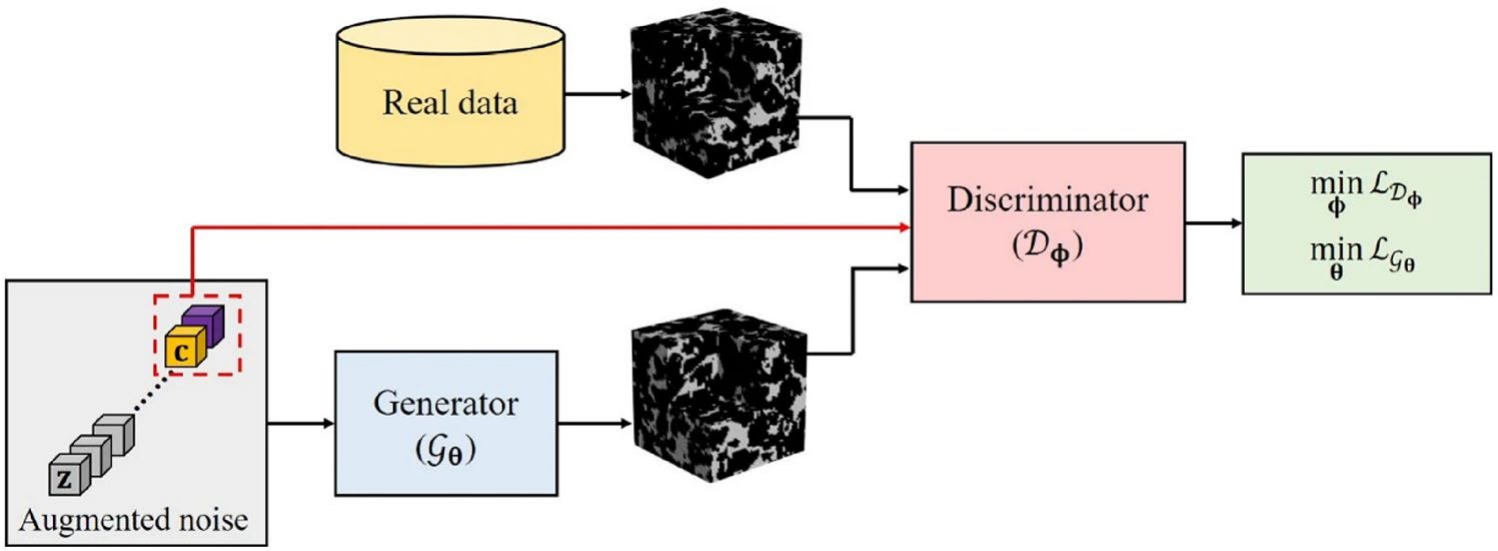
The framework of the conditional ProGAN-based method for stochastic microstructure reconstruction [240]

Further, Zheng et al. [240] combined WGAN-GP with CGAN (as illustrated in Fig 29), enabling the simultaneous characterization of different types of porous media while enhancing the representativeness of generated samples with user-defined properties. Wasserstein GAN with gradient penalty (WGAN-GP) [230, 232, 238, 240, 241, 290] has been widely adopted for stochastic microstructure reconstruction. Coiffier et al. [243, 244] were pioneers in utilizing WGAN for dimensionally-upgraded reconstruction. Kench et al. [96] proposed the SliceGAN method, which synthesizes 3D datasets using 2D images for microstructure reconstruction. Pingquan et al. [242] integrated context-aware WGAN with high-resolution optical flow estimation networks to generate virtual microstructures that effectively preserve morphological descriptors and physical properties. Additionally, Amiri et al. [246] evaluated and compared the performance of DCGAN, WGAN-GP, and StyleGAN2 in reconstructing 2D microstructures of natural rocks.

#### StyleGAN-Based Reconstruction

**Fig. 30:**
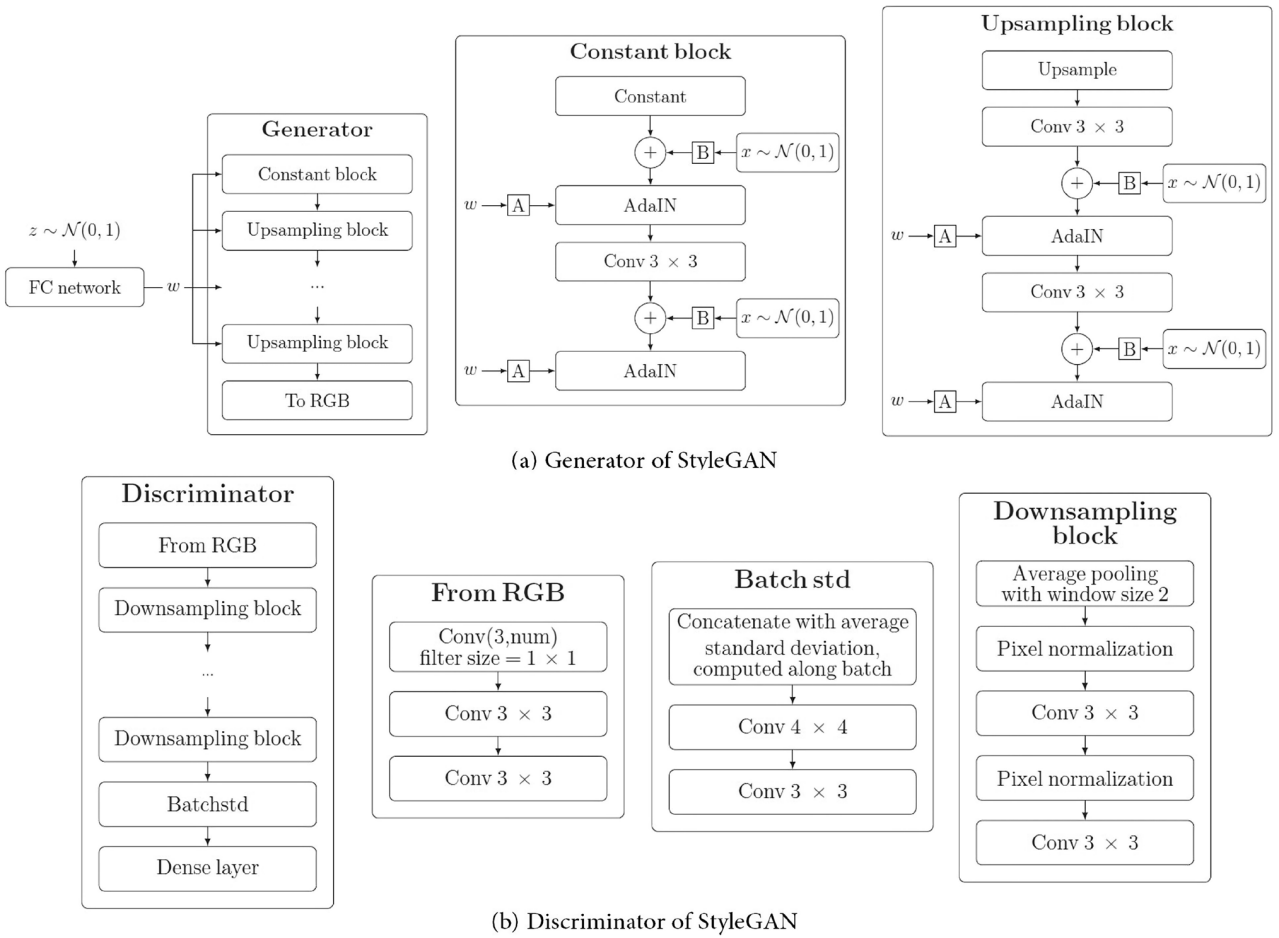
The architecture of a StyleGAN model [286]

StyleGAN was introduced to enhance the decoding of latent space information and generate higher-quality images. Its key innovation lies in incorporating"style"into the generative model, enabling fine-grained control over image details at different levels of abstraction. This is achieved through a technique known as Adaptive Instance Normalization (AdaIN). The architecture of a StyleGAN model is illustrated in Fig 30, where AdaIN is mathematically defined as:

31 $$\begin{aligned} \text {AdaIN}\big (x_i\big ) = \gamma _i\, \frac{x_i-\mu (x_i)}{\sigma (x_i)}+\beta _i\,, \end{aligned}$$

where $x_i$ represents the feature map, while $\mu (x_i)$ and $\sigma (x_i)$ denote its mean and standard deviation, respectively. The learned parameters $\gamma _i$ and $\beta _i$ modulate the normalized feature map, allowing the model to adjust style attributes dynamically.

In terms of applications, Fokina et al. [245] employed StyleGAN with image quilting to generate larger 2D images. Image quilting was specifically used to minimize boundary pixel discrepancies, ensuring a smoother transition between adjacent images of different sizes. Huang et al. [247] further refined this approach for stochastic microstructure reconstruction. Yang et al. [286] introduced a StyleGAN-based reconstruction method, where the generator begins with a set of nonlinear mapping networks and is controlled by AdaIN operations at each convolutional layer. The constant block is initialized with a combination of training data and noise, followed by AdaIN normalization. This process is applied to each channel before being forwarded to convolutional layers, and the output undergoes another round of AdaIN normalization with noise injection. The up-sampling block follows this sequence: upscale the input via a deconvolutional layer, add noise to enhance feature diversity, normalize using AdaIN to adjust style parameters, and repeat the process for further refinement.

Meanwhile, the StyleGAN discriminator consists of a series of blocks, including an input block, down sampling block, batch normalization block, and dense layer. To improve performance on small datasets, StyleGAN2-ADA introduces an adaptive discriminator augmentation (ADA) mechanism, enabling effective training with limited data. The synthesize network incorporated a style scalar to generate artificial images from low resolution ($4\times 4$) to a higher resolution by convolutional layers. The stochastic local features of generated images at different levels are controlled by injecting random noise and leaving the overall styles and high-level details intact.

#### SAGAN-Based Reconstruction

The spatially assembled GAN (SAGAN) was developed by Kim et al. [249] to stochastically generate 2D and 3D microstructures of porous media with arbitrary sizes, independent of the size of training images. The key innovation of SAGAN lies in its approach to estimating the local probability of output images from the generator using the discriminator. These local probabilities are then assembled to derive a global probability for the generated images. In standard GANs, the discriminator $D$ evaluates whether an image is sampled from the true data $X$ or generated images $X'$. However, this approach limits standard GANs in generating arbitrarily sized images while preserving the spatial characteristics of training images.

**Fig. 31:**
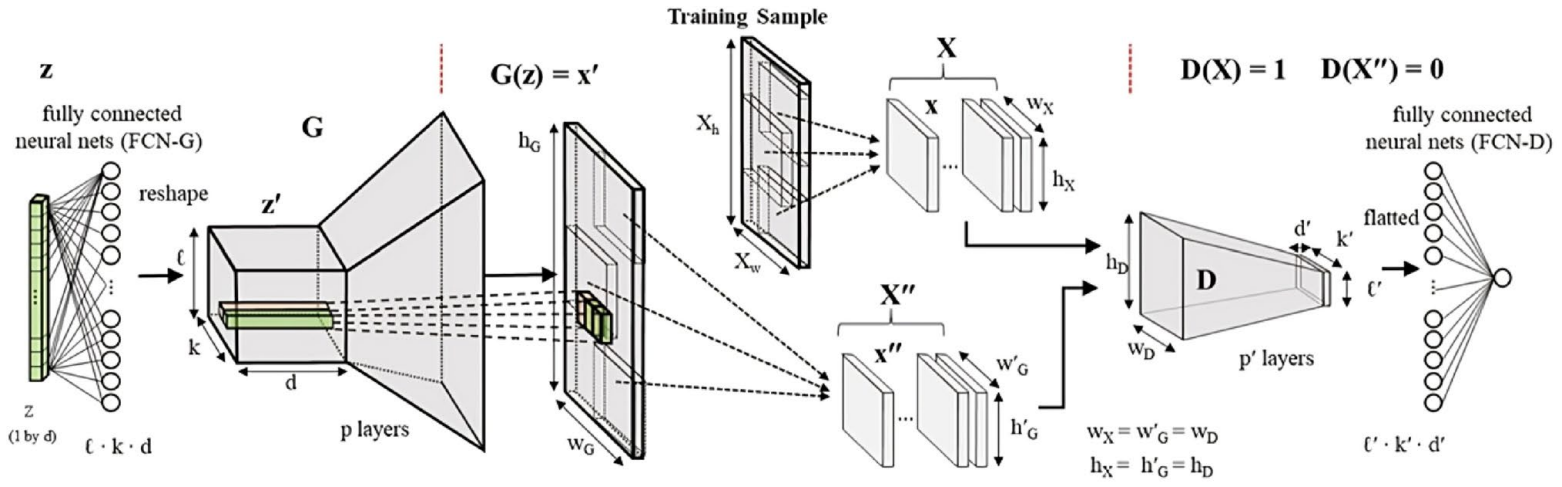
The framework of a SAGAN model [249]

SAGAN overcomes this limitation by evaluating the probability of images of arbitrary sizes that originate from the true data generation process. It does so by analyzing subsets (segments) of both the training images $X$ and the generated images $X'$, as illustrated in Fig 31. Specifically, SAGAN optimizes the generator and discriminator by examining these segments, and the corresponding loss functions are mathematically formulated as follows:

32 $$\begin{aligned} \left\{ \begin{aligned}&G(z) = \Big \{x_1'',x_2'',...,x_n''\Big \},\quad \text {where} \ X' = \bigcup _{i=1}^{n}x_i'',\ X \ni \Big \{x_1, x_2, ..., x_n\Big \}\,; \\&\mathcal {L}_D = -\frac{1}{2n}\sum _{i=1}^{n} \bigg \{ \mathbb {E}_{{\boldsymbol{x}} \sim P_{\text {data}}({\boldsymbol{x}})}\Big [\log \Big (D(x_i)\Big )\Big ] + \mathbb {E}_{{\boldsymbol{z}}\sim P_z({\boldsymbol{z}})}\Big [\log \Big (1-D\big (x_i''\big )\Big )\Big ] \bigg \}\,; \\&\mathcal {L}_G = -\frac{1}{2n}\sum _{i=1}^{n} \mathbb {E}_{{\boldsymbol{z}}\sim P_z({\boldsymbol{z}})}\Big [\log \Big (D\big (x_i''\big )\Big )\Big ]\,; \end{aligned} \right. \end{aligned}$$

where $x_i''$ and $x_i$ represent the $i$th segmented images of $X'$ and $X$, respectively. Each segment contains multiple projective fields to ensure the smoothness and connectivity of the fully generated image.

In SAGANs, segments are designed to contain multiple projective fields (PFs) to ensure the smoothness and connectivity of the generated whole images. Each PF is attached to adjacent ones, creating a high degree of correlation, where a change in one PF affects all neighboring PFs. Consequently, regardless of the size of the images generated by the generator, the architecture of the discriminator remains independent of the generator. The performance of SAGANs has been evaluated for equidimensional reconstruction using metrics such as the two-point probability function, two-point cluster function, and permeability. Additionally, Laloy et al. [248] have applied SAGAN for both 2D-to-2D and 3D-to-3D microstructure reconstruction, further demonstrating its capability in generating realistic porous media structures.

#### Other Variants of GAN-Based Reconstruction

BicycleGAN, a variant of GAN, features symmetric encoding and decoding modules. Feng et al. [233, 234] applied a U-Net-based BicycleGAN for the stochastic reconstruction of random porous microstructures, where the input consists of a random field along with a small patch taken from real images. This BicycleGAN-based method demonstrated both high accuracy and efficiency in generating porous materials with a variety of morphologies. Further extending this work, Feng et al. [250] developed a method for 2D-to-3D microstructure reconstruction using BicycleGAN. To achieve dimensionally-upgraded reconstruction, the network architecture of BicycleGAN was redesigned. The generator’s input consists of a 2D image with dimensions $1 \times N \times N$ and a standard Gaussian noise vector of dimensions $n_z \times 1 \times 1$. The last two dimensions of the noise vector are expanded into 2D images, which are subsequently stacked together to form a 3D structure with dimensions $N \times N \times N$. This approach was applied to both isotropic and non-stationary porous materials, with quantitative comparisons performed between real and reconstructed porous microstructures. The results demonstrated the method’s effectiveness in preserving essential material properties during the reconstruction process.

**Fig. 32:**
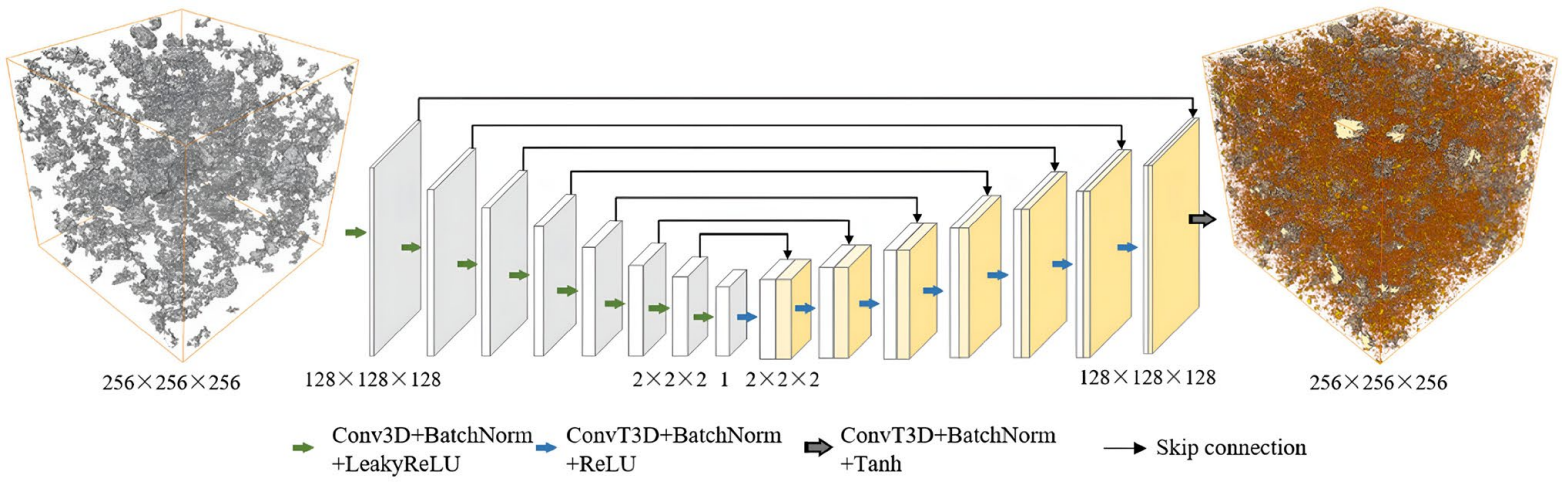
The architecture of U-net in the generator of a pix2pix GAN model [251]

A multiscale reconstruction method was developed by Yang et al. [251], in which the pix2pix GAN [291] was employed for stochastic characterization and reconstruction of random porous microstructures. As illustrated in Fig 32, the generator follows a U-Net framework with skip connections, which help preserve low-resolution information and guide the upscaling process for generating high-resolution morphologies. To train the pix2pix GAN model, high-resolution images with a narrow field of view were paired with their corresponding low-resolution images that encompassed a larger field of view. This strategy enables the generation of multiscale porous structures while maintaining fine morphological details. The pix2pix GAN-based reconstruction method is capable of producing 3D microstructural samples with arbitrary volume sizes, overcoming the limitations of convolution-based generators in most GAN models, which are typically constrained to generate images of the same size and resolution as the training data.

**Fig. 33:**
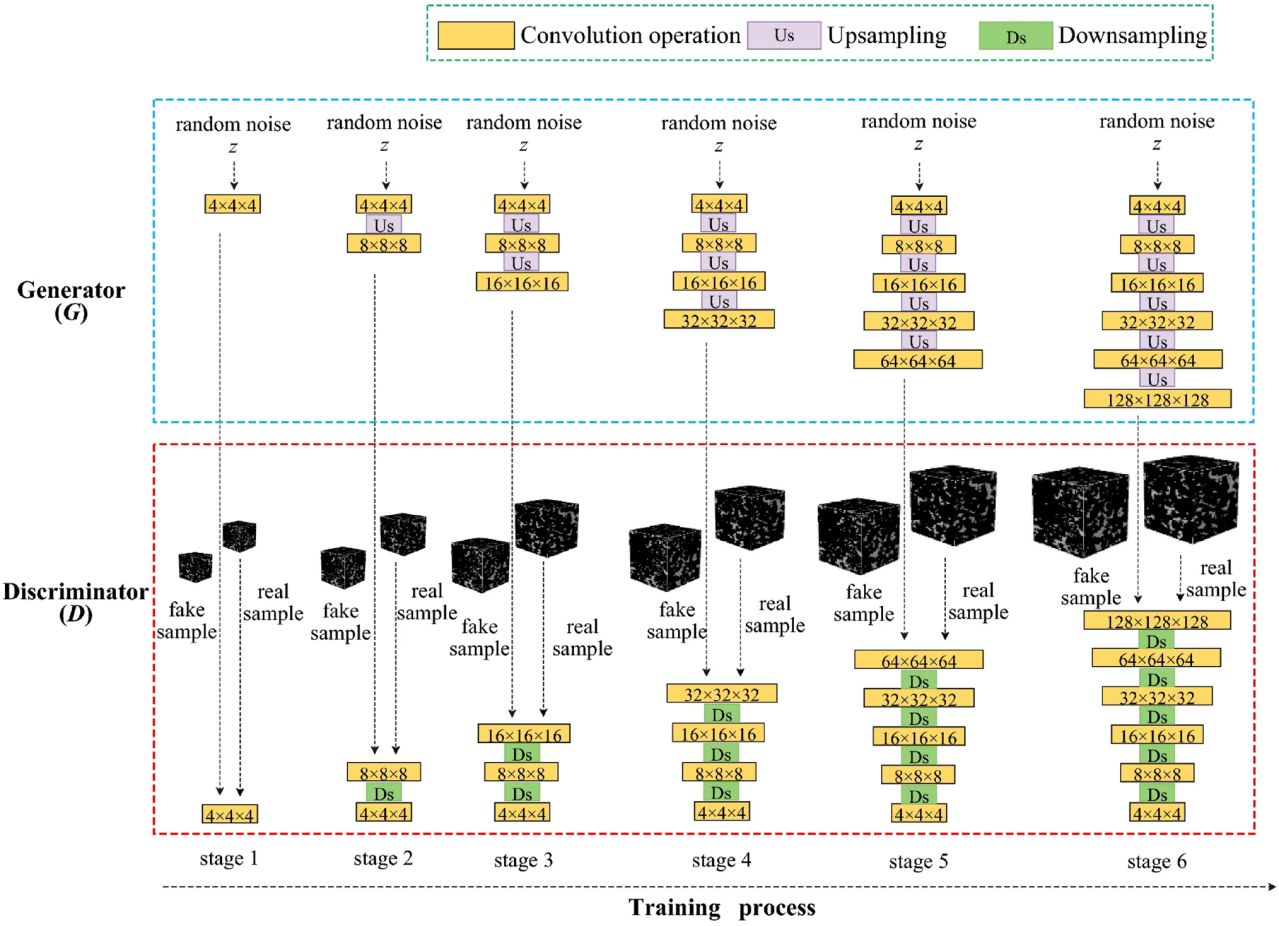
The architecture of the MS-GAN model for stochastic microstructure reconstruction [255]

Inspired by StackGAN [292] and LapGAN [293], Xia et al. [255] proposed a multiscale generative adversarial network (MS-GAN) to generate porous microstructure samples of arbitrary size. In this approach, trilinear interpolation was employed for upscaling and downscaling images, while the Wasserstein GAN with gradient penalty (WGAN-GP) loss function was utilized to stabilize the training process. The architecture of MS-GAN is graphically illustrated in Fig 33. A convolutional layer and an upsampling layer were incorporated into the generator, whereas a convolutional layer and a downsampling layer were added to the discriminator. During the training process, random noise with dimensions $4 \times 4 \times 4$ voxels is input into the generator $G$, which subsequently produces synthetic samples of the same size. These generated samples, along with real microstructures, are then fed into the discriminator $D$ for classification. Once the loss function converges to a stable state, the training process at the current stage is completed. To achieve the desired resolution and scale, a series of upsampling and downsampling operations are applied to the generated microstructure samples.

### Sequence-Based Reconstruction

Sequence-based reconstruction methods leverage deep learning models designed to capture spatial dependencies and reconstruct 3D microstructures in a sequential manner. Unlike direct volumetric generation approaches, these methods adopt a layer-by-layer reconstruction strategy, where each subsequent layer is predicted based on prior layers. This approach effectively preserves spatial continuity and morphological consistency, making it particularly useful for generating large-scale porous media while reducing computational costs.

#### Recurrent Neutral Network-Based Reconstruction

**Fig. 34:**
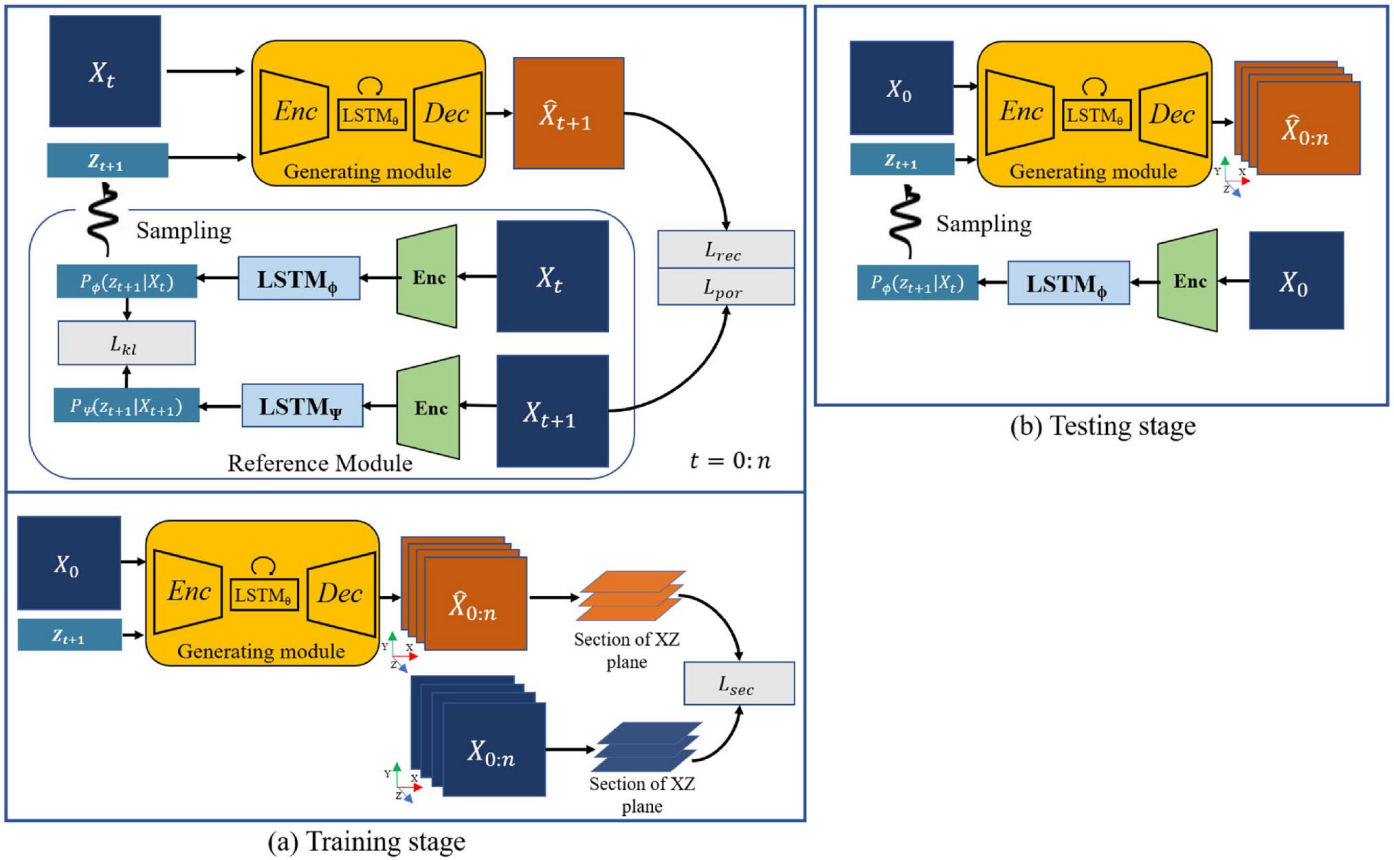
The framework of the RNN-based method for stochastic microstructure reconstruction [259]

Recurrent neural networks (RNNs) have been applied to stochastic reconstruction of 3D porous microstructure samples based on 2D reference images [257]. As illustrated in Fig 34, the RNN-based reconstruction framework consists of a reference module and a generating module. In the training stage, the RNN model learns spatial correlations from sequential image data. During the testing stage, it predicts future layers along the depth direction. The reference module, comprising an encoder, a learned prior network ($LSTM_{\phi }$), and an inference network ($LSTM_{\psi }$), is designed to capture the prior distribution of the predicted layer while establishing spatial dependencies between the current and future layers. The generating module, responsible for predicting subsequent layers based on the current one, includes an encoder, a decoder, and a frame predictor ($LSTM_{\theta }$). This architecture ensures that the generated 3D porous microstructures accurately preserves spatial continuity and morphological consistency.

During the training stage, the input data consists of a sequence of 2D images along the layer direction. These images are compressed into feature vectors using a series of encoders, denoted as $Enc(\cdot )$, for dimensionality reduction. The learned prior network $LSTM_{\phi }$ and the inference network $LSTM_{\psi }$ process these feature vectors to learn the prior distribution $P_{\phi }\big (z_{t+1} \vert X_t \big )$ and the inference distribution $P_{\psi }\big (z_{t+1} \vert X_{t+1}\big )$, respectively. These distributions capture spatial dependencies between consecutive image layers. The generating module then reconstructs the next layer $\widehat{X}_{t+1}$ based on the current layer $X_t$, while the reference module measures the similarity between real and generated data. During the testing stage, all parameters of the generating and reference modules remain fixed, and the generating module is used to predict subsequent layers from the initial reference layer $X_0$. The predictor network of the generator $LSTM_{\theta }$ receives the feature vector $z_{t+1}$ as input and generates a new feature vector ${\boldsymbol{g}}_{t+1}$, which is then passed to the decoder $Dec(\cdot )$ to reconstruct a new image. This process can be mathematically described as follows:

33 $$\begin{aligned} \left\{ \begin{aligned}&P_{\phi }\Big (z_{t+1} \Big \vert X_t\Big ) = LSTM_{\phi }\Big ({Enc\big (X_t\big )}\Big )\,, \\&{\boldsymbol{z}}_{t+1} \sim P_{\phi }\Big (z_{t+1} \Big \vert X_t\Big )\,, \\&{\boldsymbol{g}}_t = LSTM_{\theta }\Big ({Enc\big (X_t\big )},\, z_{t+1}\Big )\,, \\&\widehat{X}_t = Dec\big ({\boldsymbol{g}}_t\big )\,, \end{aligned}\right. \end{aligned}$$

The total loss function of the RNN-based framework is the weighted sum of reconstruction discrepancy, porosity error, and Kullback-Leibler (KL) divergence:

34 $$\begin{aligned} \mathcal {L}_{\textrm{total}}&= \lambda _{\textrm{rec}}\mathcal {L}_{\textrm{rec}}+\lambda _{\textrm{por}} \mathcal {L}_{\textrm{por}} +\lambda _{\textrm{KL}}\mathcal {L}_{\textrm{KL}}\\&=\lambda _{\textrm{rec}} \frac{1}{n} \sum _{i=1}^{n}\bigg \Vert X_{i} - \widehat{X}_{i} \bigg \Vert _{1} +\lambda _{\textrm{por}} \bigg \Vert X_{1:n}^{\textrm{porosity}}-\widehat{X}_{1:n}^{\textrm{porosity}} \bigg \Vert _2 +\lambda _{\textrm{KL}} E\bigg [\log \Big( P_{\phi }\big ({\boldsymbol{z}}_{t+1}\vert X_t\big)\Big) -\log \Big( P_{\psi }\big ({\boldsymbol{z}}_{t+1}\vert X_t\big )\Big) \bigg ]\,, \end{aligned}$$

This RNN-based reconstruction method has been tested on both isotropic and anisotropic porous media, and its effectiveness and accuracy have been validated using three different morphological descriptors.

**Fig. 35:**
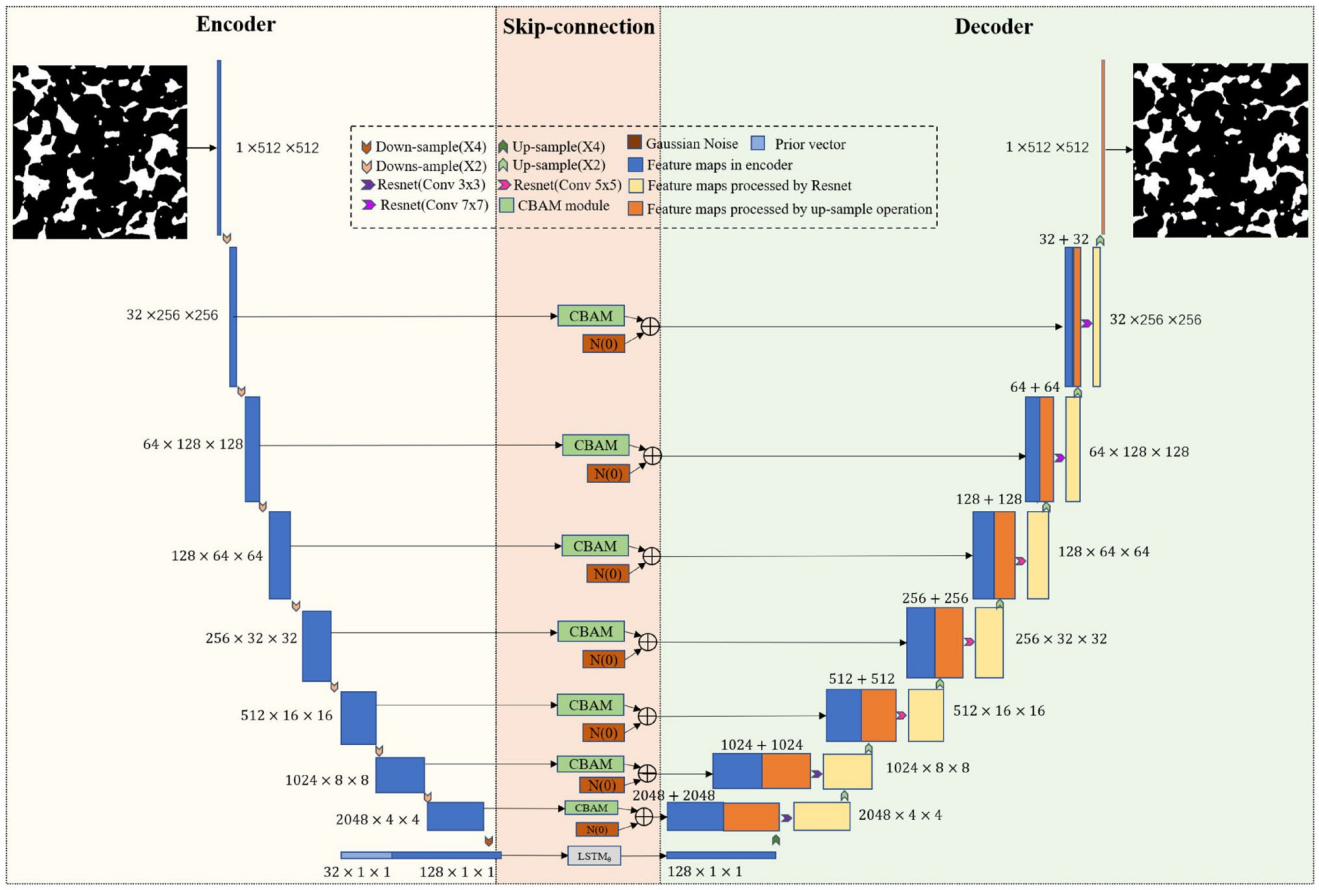
The architecture of the modified 3D-PMRNN model with skip connection [259]

In Zhang’s later work [259], the size of synthetic realizations was increased from $256^3$ to $512^3$. However, continuity along the *z*-direction exhibited poor performance in the original 3D-PMRNN model. To improve this, they introduced a hybrid receptive field combining CNNs in the encoder-decoder stage with an attention-based block in the skip-connection module. This architecture allows for the capture of a wider range of 2D information across different reconstruction stages, as shown in Fig 35. They also modified the loss function by adding a section loss, which provides additional morphological information of the 3D microstructure from the *y*-direction. Specifically, *xz*-planar slices are extracted every 100 layers along the *y*-direction from both the training and generated sequences. A pre-trained VGG-16 network is then employed to capture high-level features and evaluate the perceptual similarity between the input images and their generated counterparts.

Additionally, Zhang’s team proposed an RNN-based generative model, incorporating a GAN, to address the 2D-to-3D reconstruction problem. This model, called PM-ARNN [258], is better at capturing the overall morphological characteristics of porous media and improving the statistical features of synthetic realizations. The RNN-based reconstruction method enables the generation of large-scale realizations (e.g., $256^3$ or larger) with reduced GPU memory demands and a more stable training process. The efficiency of this approach was compared with that of Bicycle-GAN. The method’s accuracy has been validated in terms of morphological descriptors and permeability. Notably, they claim that the RNN-based method requires only a single 3D sample for training, thus making it a true 3D-to-3D reconstruction approach. However, it is important to note that 3D images are still required for training. Furthermore, the method is based on the assumption of directional continuity in one direction, a concept that has not been widely recognized or validated.

#### Generative Pre-Training-Based Reconstruction

**Fig. 36:**
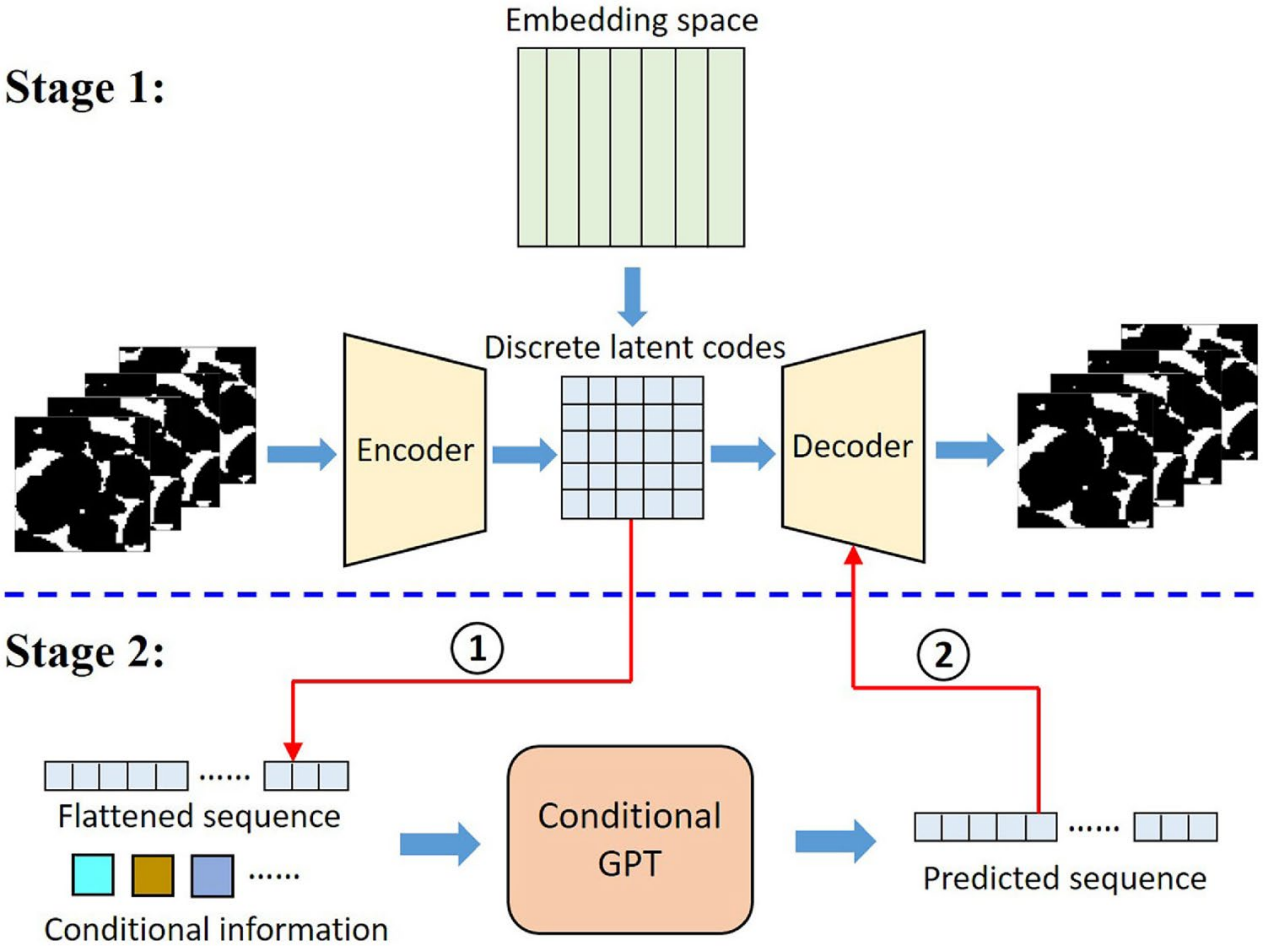
The workflow of RockGPT for stochastic characterization and microstructure reconstruction [260]

Similar to the RNN-based approach, which follows a layer-by-layer reconstruction strategy, Zheng et al. [260] proposed RockGPT, a method that integrates vector quantized variational autoencoders (VQ-VAE) and conditional generative pre-training (GPT) to reconstruct various types of porous microstructures while ensuring user-defined properties. Like RNNs, GPT excels at capturing long-range dependencies in sequential data, due to its attention mechanism. Building on the standard GPT framework, a conditioning block was introduced to incorporate additional contextual information, such as rock type and porosity. The overall architecture of RockGPT is illustrated in Fig 36, which consists of two key stages:

- Stage 1 - Learning a discretized latent space: Vector quantized variational autoencoders (VQ-VAE) is first trained to encode high-dimensional images into a discrete latent space;
- Stage 2 - Sequence modeling and reconstruction: The discrete latent codes are then flattened and processed using a conditional GPT model, which generates a new sequence of latent codes while integrating the additional conditioning information. Finally, the generated latent codes are decoded back into an image representation.

#### Transformer-Based Reconstruction

**Fig. 37:**
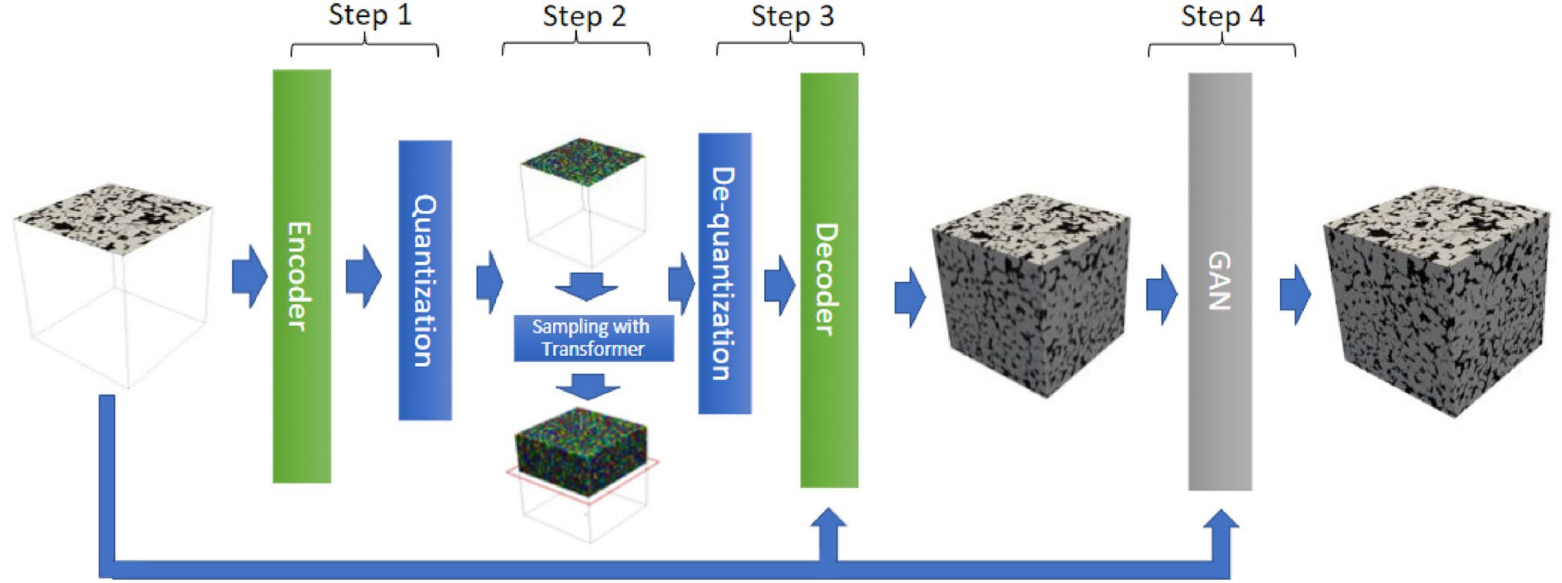
The workflow of microstructure reconstruction via the combination of VQ-VAE, GAN and image transformer [261]

Furthermore, Johan Phan et al. [261] proposed a size-invariant multi-step 3D reconstruction method that integrates VQ-VAE, GAN and an image transformer. In this approach, 2D training images are first processed by an encoder to obtain the first layer of the quantized vector. This quantized vector is then passed through the transformer, which predicts the likelihood distribution of the next data point. Sampling is performed using a top-*k* strategy with $k=10$. This workflow enables the transformer to capture global microstructural features, while the GAN refines the output by enhancing fine-grained textures. The complete workflow transformer-based method for stochastic microstructure reconstruction is illustrated in Fig 37. Additionally, details on VAE and GAN can be found in above sections.

### Generative Flow Network-Based Reconstruction

Generative flow (*Glow*) networks are likelihood-based generative models that employ a series of invertible transformations to construct models with a tractable density function. Anderson et al. [262] and Guan et al. [263] pioneered the use of generative flow models for synthesizing 3D porous microstructures, demonstrating their effectiveness in reconstructing porous sandstones. Flow-based models optimize a likelihood-based objective by minimizing the negative log-likelihood of the data points ${\boldsymbol{x}}$ in the *Glow* model:

35 $$\begin{aligned} \mathcal {L}\big (\mathcal {D}\big )=\frac{1}{N}\sum _{i=1}^{N}-\log {p_{\theta }}\left( {\boldsymbol{x}}^{(i)}\right) \,, \end{aligned}$$

where $\mathcal {L}(\cdot )$ represents the likelihood, ${\boldsymbol{x}}$ is a random vector sampled from the true distribution ${\boldsymbol{x}} \sim p^*({\boldsymbol{x}})$, $\mathcal {D}$ denotes the dataset, and $p_{\theta }$ is the model parameterized by $\theta$.

The *Glow* model represents the data points ${\boldsymbol{x}}$ as:

36 $$\begin{aligned} \left\{ \begin{aligned}&{\boldsymbol{z}} \sim p({\boldsymbol{z}})\,,\\&{\boldsymbol{x}} = {\boldsymbol{g}}_{\theta }({\boldsymbol{z}})\,, \end{aligned} \right. \end{aligned}$$

where ${\boldsymbol{z}}$ is the latent variable sampled from a known density function, often a Gaussian distribution $p({\boldsymbol{z}})=\mathcal {N}\big ({\boldsymbol{\mu }}, {\boldsymbol{\Sigma }}\big )$. The function ${\boldsymbol{g}}_{\theta }(\cdot )$ is invertible, enabling latent variable inference:

37 $$\begin{aligned} {\boldsymbol{z}} = {\boldsymbol{f}}_{\theta }({\boldsymbol{x}}) = {\boldsymbol{g}}^{-1}({\boldsymbol{x}})\,, \end{aligned}$$

**Fig. 38:**
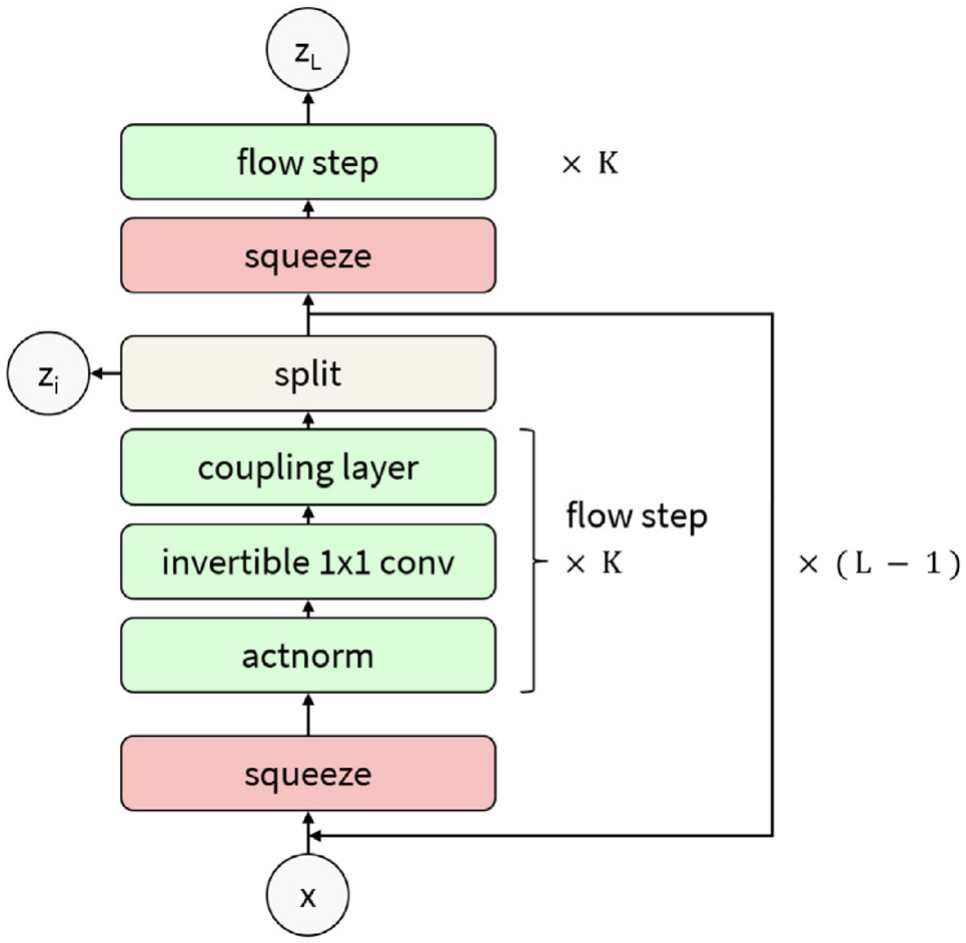
The architecture of a generative flow (*Glow*) network model [263]

Using the change-of-variables formula, the log-likelihood is expressed as:

38 $$\begin{aligned} \log {p_{\theta }}({\boldsymbol{x}})=\log {p_{\theta }}({\boldsymbol{z}})+\sum _{i=1}^{K}\log \Big \vert \det \big (\textrm{d}h_i/\textrm{d}h_{i-1}\big )\Big \vert \,, \end{aligned}$$

where $h_0 \equiv {\boldsymbol{x}}$ and $h_K \equiv {\boldsymbol{z}}$. The architecture of the *Glow* model is illustrated in Fig 38. Each flow step consists of three key components: an actnorm layer (normalizing activations), an invertible 1$\times$1 convolution (ensuring invertibility), and an affine or additive transformation layer (effectively modifying feature representations).

### Diffusion Model-Based Reconstruction

**Fig. 39:**
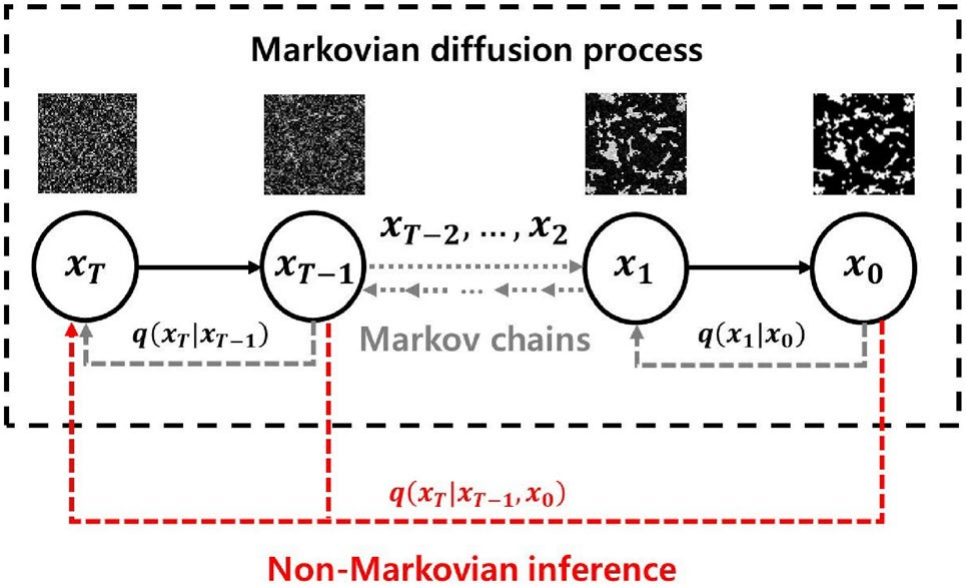
Schematic diagram of Markovian diffusion process and non-Markovian inference models for microstructure synthesis [264]

Lee at al. [264] developed a novel approach for microstructure reconstruction using denoising diffusion probabilistic models (DDPMs), addressing the limitations of traditional descriptor-based and GAN-based methods. Unlike GANs, which suffer from training instability and mode collapse, diffusion models iteratively transform noise into structured microstructures through a learned denoising process. As illustrated in Fig 39, the forward process progressively adds Gaussian noise to a microstructure sample $\textbf{x}_0$, following the Markov chain formulation:

39 $$\begin{aligned} q\big (\textbf{x}_t | \textbf{x}_{t-1}\big ) = \mathcal {N}\Big [\textbf{x}_t; \sqrt{\alpha _t} \textbf{x}_{t-1}, (1 - \alpha _t) \textbf{I}\Big ]\,, \end{aligned}$$

where $\alpha _t$ controls the noise schedule. The reverse process, parameterized by a deep neural network, learns to denoise the sample step by step, approximating the posterior distribution:

40 $$\begin{aligned} p_{\theta }\big (\textbf{x}_{t-1} | \textbf{x}_t\big ) = \mathcal {N}\Big [\textbf{x}_{t-1}; \mu _{\theta }(\textbf{x}_t, t), \Sigma _{\theta }(\textbf{x}_t, t)\Big ]\,. \end{aligned}$$

This diffusion model-based framework has been applied to reconstruct diverse random microstructures, including polycrystalline alloys, ceramics, and fiber composites. To accelerate sampling, the authors integrated denoising diffusion implicit models (DDIMs), which reduce the number of required timesteps while maintaining sample fidelity. The generated microstructures are evaluated using Fréchet inception distance, precision-recall curves, and statistical descriptors such as the two-point correlation function. Compared to GAN-based and descriptor-based reconstruction approaches, diffusion models demonstrate superior generalization, better sample diversity, and enhanced stability. The study highlights diffusion models as a powerful tool for data-driven materials design, with potential for further optimization in hyper-parameter tuning and latent-space exploration.

### Hybrid Model-Based Reconstruction

Hybrid modeling approaches combine multiple machine/deep learning techniques to enhance the stochastic characterization and reconstruction of random porous microstructures. By leveraging the strengths of different models while mitigating their individual limitations, these methods improve accuracy and robustness. Recent advancements in hybrid modeling include coupling variational autoencoders (VAE) or autoencoders (AE) with generative adversarial networks (GANs), merging convolutional neural networks (CNNs) with GANs, and integrating VAEs with CNNs.

#### VAE/AE-GAN-Based Reconstruction

Autoencoders (AE), variational autoencoders (VAE), and generative adversarial networks (GANs) each offer distinct advantages, making them highly complementary in microstructure reconstruction [294]. Leveraging this synergy, Zhang et al. [268] developed a VAE-GAN-based framework for stochastically synthesizing 3D porous microstructures from a single 2D image. Their approach successfully generated both isotropic and anisotropic porous media, demonstrating strong agreement with statistical function matching. Other studies have further explored VAE-GAN models for stochastic microstructure reconstruction. Kononov et al. [269] and Li et al. [270] applied VAE-GAN techniques for 2D-to-3D reconstruction, while Banko et al. [266] developed a VAE-GAN-based method for 2D-to-2D reconstruction. Extending this approach, Zhang et al. [267] introduced a 3D-to-3D microstructure reconstruction model, further broadening its applicability to complex material systems.

**Fig. 40:**
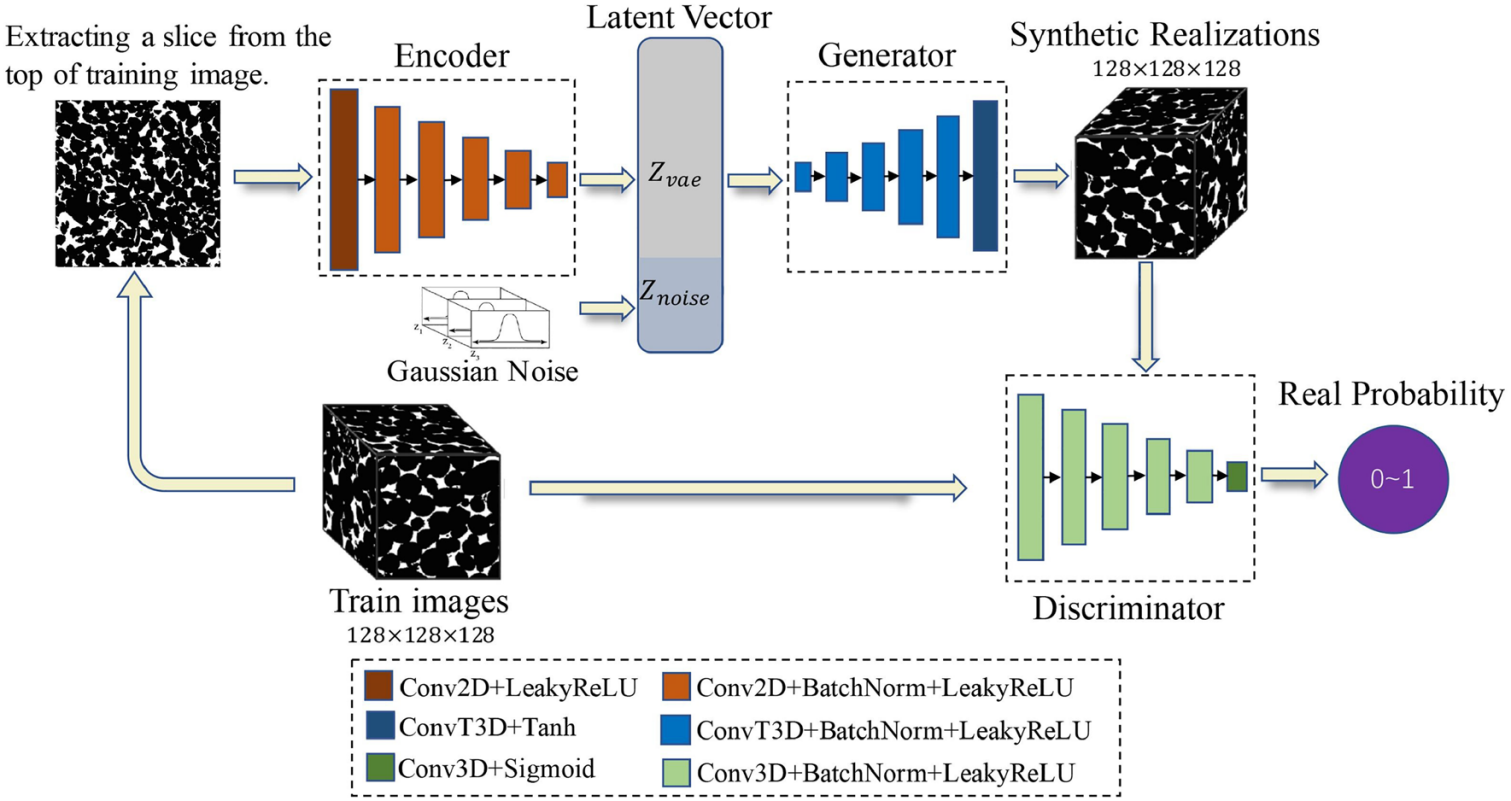
The framework of the VAE-GAN-based method for 2D-to-3D microstructure reconstruction [268]

In Fig 40, the VAE-GAN architecture integrates the encoder of VAE with the generator of GAN to enhance microstructure synthesis. The encoder extracts features from 2D training images, encoding them into latent vectors, which are then fed into the GAN generator. This setup enables the generator to gain a deeper understanding of the microstructural features within the training images. Instead of a VAE decoder, the GAN discriminator is employed to refine the synthetic realizations through an adversarial training strategy. The VAE loss function consists of the sum of the reconstruction error and a prior regularization term, defined as:

41 $$\begin{aligned} \left\{ \begin{aligned}&\mathcal {L}_{\textrm{VAE}} = \mathcal {L}_{\textrm{like}}^{\textrm{pixel}} + \mathcal {L}_{\textrm{prior}}\,,\\&\mathcal {L}_{\textrm{like}}^{\textrm{pixel}} = \mathbb {E}_{{\boldsymbol{z}}} \Big [\log \Big (p_{\theta }({\boldsymbol{x}}\vert {\boldsymbol{z}})\Big )\Big ]\,,\\&\mathcal {L}_{\textrm{prior}} =-D_{\textrm{KL}}\Big [q_{\psi }({\boldsymbol{x}}\vert {\boldsymbol{z}})\big \Vert p({\boldsymbol{x}}\vert {\boldsymbol{z}})\Big ]\,. \end{aligned} \right. \end{aligned}$$

The generator loss function is a weighted sum of the reconstruction error, the standard GAN generator loss, and a porosity error term, formulated as:

42 $$\begin{aligned} \mathcal {L}_{\textrm{total}}^{(G)} = \lambda _{\textrm{vae}}\times \mathcal {L}_{\textrm{like}}^{\textrm{pixel}} + \lambda _{\textrm{gan}}\times \mathcal {L}_{\textrm{gan}}^{(G)} + \lambda _{\textrm{porosity}} \times \mathcal {L}_{\textrm{porosity}}^{(G)}\,, \end{aligned}$$

where $\mathcal {L}_{\textrm{porosity}}^{(G)}$ is the porosity error designed to identify the disagreement of the porosity between the realizations and the training images, and it is defined as follows:

43 $$\begin{aligned} \mathcal {L}_{\textrm{porosity}}^{(G)} = \Big \Vert {\boldsymbol{x}}_{\textrm{porosity}} - {\boldsymbol{G}}_{\textrm{porosity}}\Big ({\boldsymbol{z}}_{\textrm{vae}},{\boldsymbol{z}}_{\textrm{noise}}\Big ) \Big \Vert _2\,. \end{aligned}$$

Additionally, the standard autoencoder (AE) has also been integrated with GAN for microstructure reconstruction, with AE serving as a data compression tool that projects high-dimensional image data onto lower-dimensional latent vectors. Shams et al. [271] combined AE and GAN models for 3D-to-3D microstructure reconstruction, leveraging AE to compress and capture the essential features of the input data. Building on this approach, Volkhonskiy et al. [273] advanced the technique to generate 3D images from 2D slices. Rather than using a series of 2D slices, they extracted the central slice from the 3D training image, encoding it into a latent vector representation. The generator then serves as the decoder, synthesizing the 3D image by inputting the latent representation along with a noise vector. This method highlights the versatility of combining AE with GAN to reconstruct complex microstructures with improved efficiency and flexibility.

#### SinGAN-Attention-Based Reconstruction

**Fig. 41:**
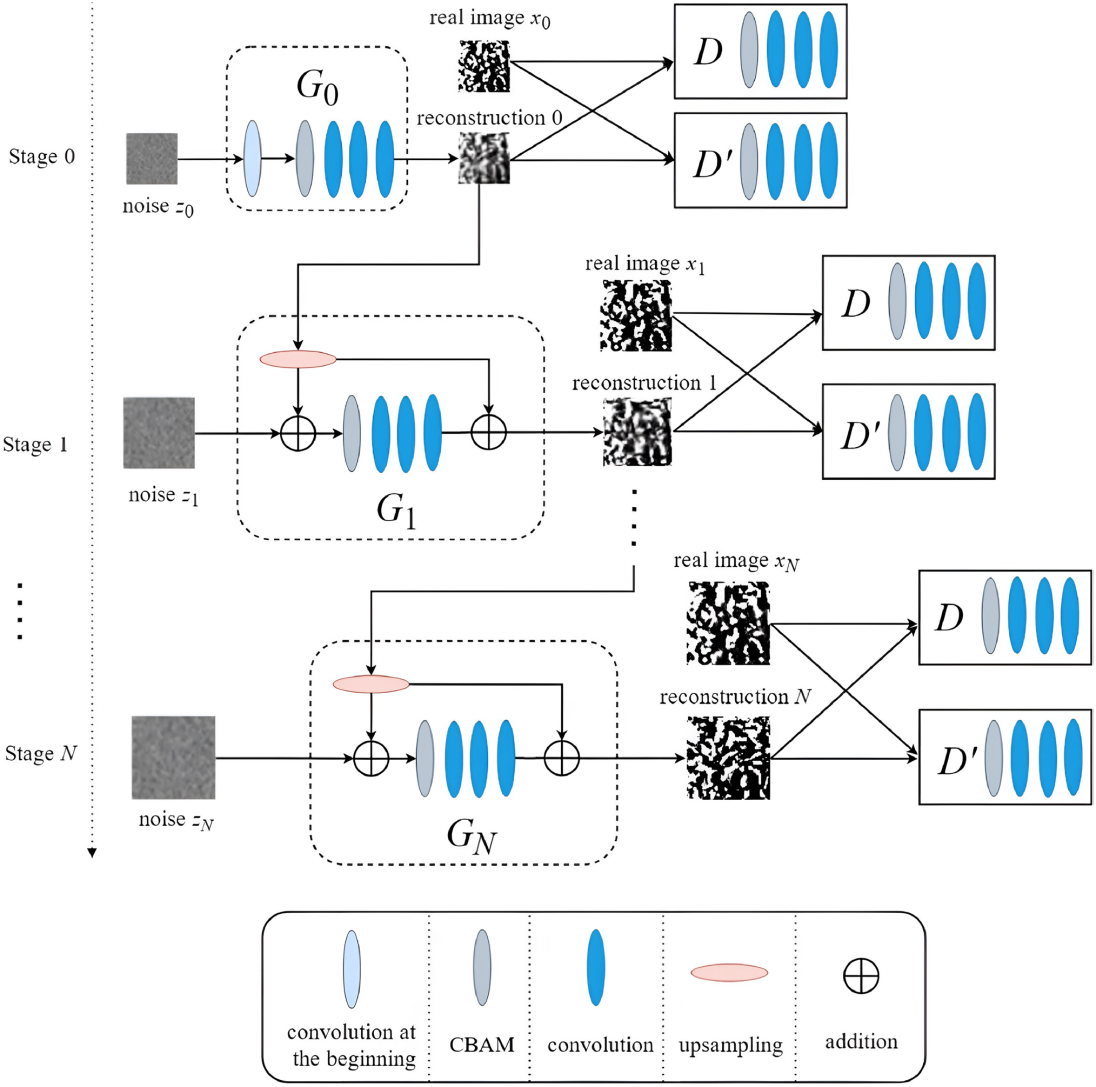
The framework of the multi-stage SinGAN-based method for stochastic microstructure reconstruction [252]

Zhang et al. [252] integrated SinGAN [295] with spatial attention mechanisms to achieve super-resolution reconstruction, requiring only a single scanning image of natural porous media for model training. As depicted in Fig 41, the training process is structured into a series of independent stages, where images with progressively higher resolution are generated at deeper stages. To maintain stability and consistency, each stage must be completed before advancing to the next, with the parameters of the completed stages fixed. Building on this framework, Zhang et al. further refined this hybrid GAN-based reconstruction technique in their subsequent studies [253, 254], introducing novel strategies to enhance reconstruction fidelity and scalability for random porous microstructures.

#### InfGAN-StyleGAN-Based Reconstruction

**Fig. 42:**
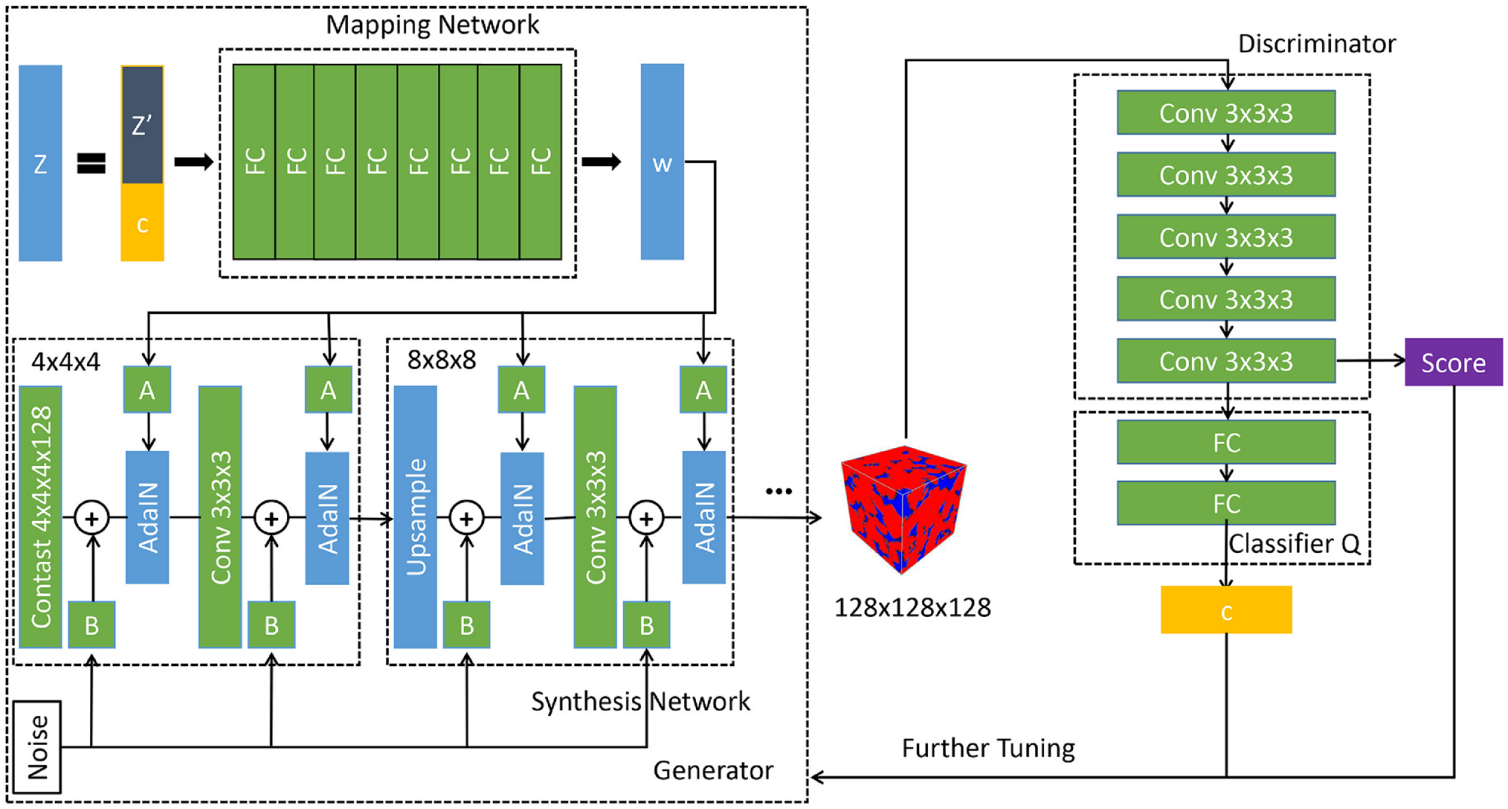
The framework of the InfGAN-StyleGAN-based method for stochastic microstructure reconstruction [274]

Cao et al. [274] introduced a hybrid GAN framework that integrates InfoGAN and StyleGAN, referred to as CISGAN (see Fig 42). This approach enhances microstructure reconstruction by incorporating the volume fraction of porous media, even when training data is limited. In CISGAN, the porosity distribution is embedded within the latent space to regulate pore morphology, while a classifier within the discriminator constrains porosity within reasonable bounds. InfoGAN, structurally similar to CGAN, introduces an additional classifier *Q* in the discriminator to extract conditional information, ensuring that the generator adheres to user-specified constraints. Meanwhile, the style-based GAN component mitigates mode collapse, a common issue when training on small datasets. Experimental results demonstrate that the generated microstructure samples align well with target specifications, showcasing the effectiveness of this InfGAN-StyleGAN-based reconstruction method.

#### StyleGAN-CycleGAN-Based Reconstruction

**Fig. 43:**
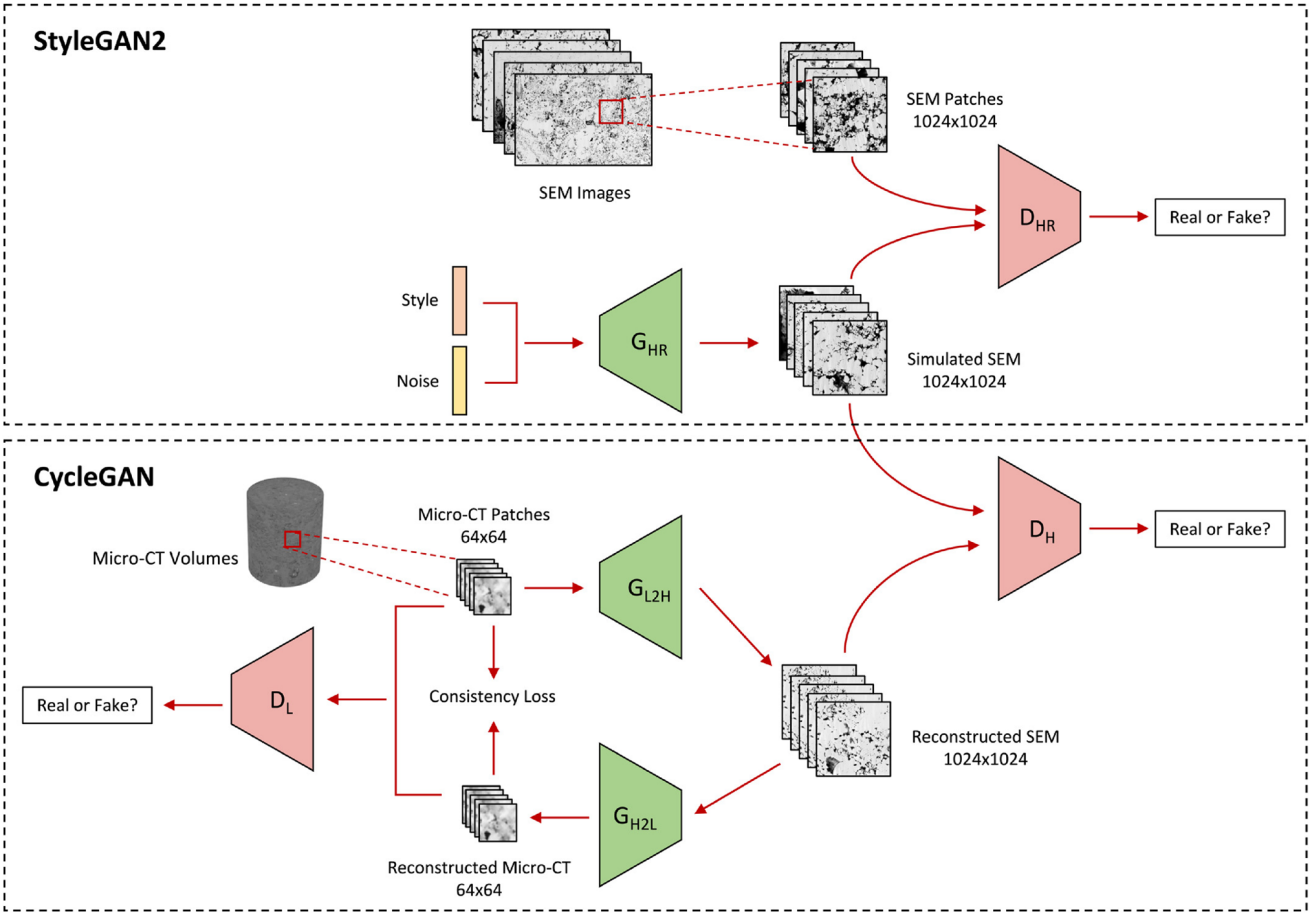
The framework of the StyleGAN-CycleGAN-based method for stochastic microstructure reconstruction [275]

Liu et al. [275] proposed a hybrid reconstruction framework that combines a style-based GAN with a cycle-consistent GAN for digital rock microstructure synthesis, as illustrated in Fig 43. This approach leverages StyleGAN for augmenting limited high-resolution data while utilizing CycleGAN to integrate unpaired low- and high-resolution digital rock datasets from various sources. CycleGAN is an unsupervised approach for image cross-domain transfer, allowing micro-CT images to be mapped from a low-resolution domain to a high-resolution domain while handling the challenge of unpaired training samples. The CycleGAN framework consists of four networks, which are:

- Generator $G_{L2H}$ maps images from the low-resolution domain ($LR$) to the high-resolution domain ($HR$).
- Generator $G_{H2L}$ maps images from the high-resolution domain ($HR$) to the low-resolution domain ($LR$).
- Discriminator $D_H$ encourages $G_{L2H}$ to generate outputs that are indistinguishable from real high-resolution images.
- Discriminator $D_L$ encourages $G_{H2L}$ to generate outputs that are indistinguishable from real low-resolution images.

This framework ensures that $G_{L2H}$ generates high-resolution images conditioned on the input low-resolution images, and vice versa for $G_{H2L}$. During the training process, StyleGAN2-ADA is first trained for scanning electron microscopy (SEM) data augmentation, and its generator is used to synthesize high-resolution images, ensuring compatibility with low-resolution data. Subsequently, CycleGAN is trained, with its generator $G_{L2H}$ applied to transfer low-resolution images from $64 \times 64$ to $1024 \times 1024$ pixels. The effectiveness and accuracy of this hybrid microstructure reconstruction method are evaluated based on porosity, specific perimeter, two-point correlation, and effective permeability.

#### VQVAE-PixelCNN-Based Reconstruction

**Fig. 44:**
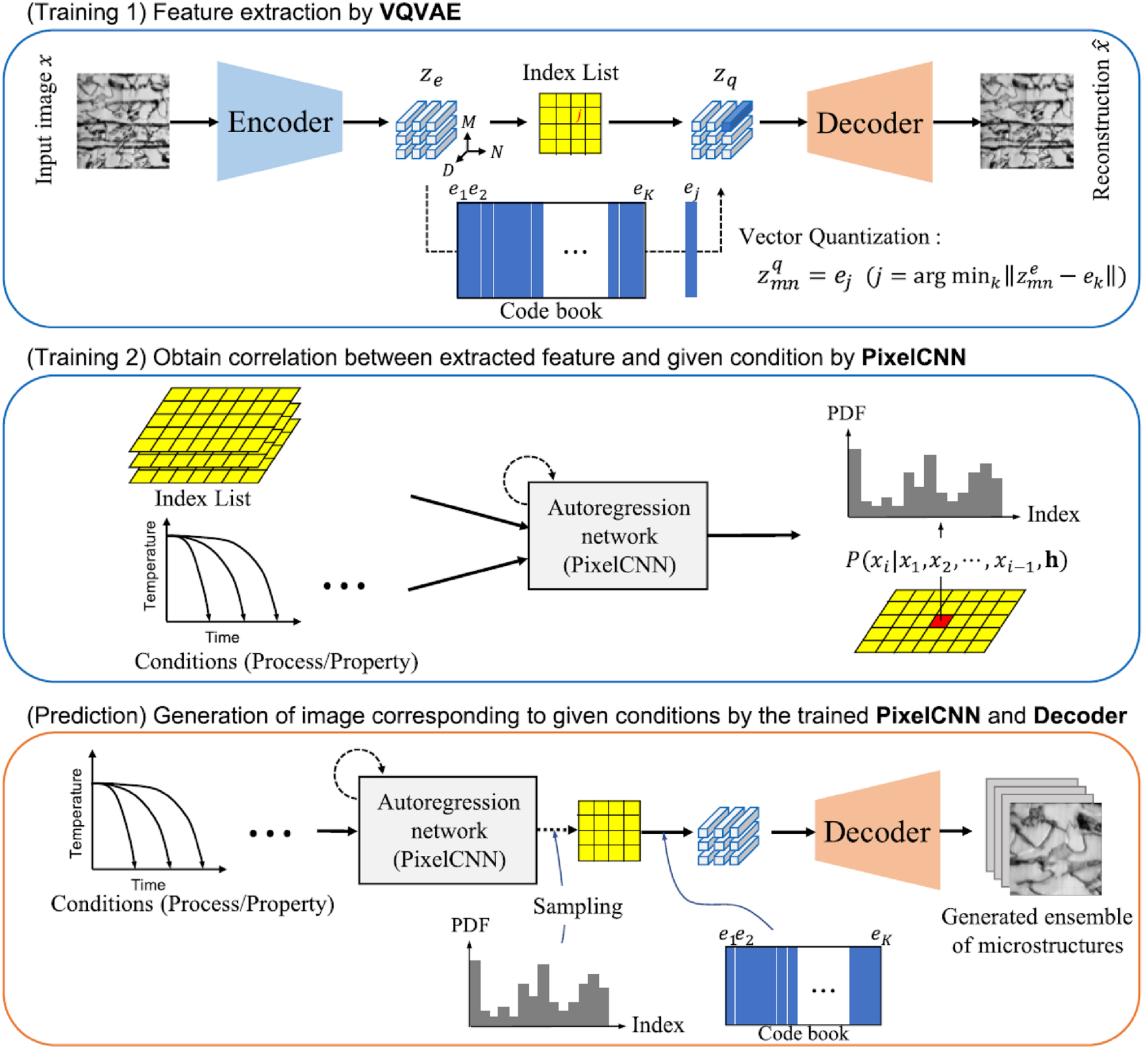
The workflow of the VQVAE-PixelCNN-based method for stochastic microstructure reconstruction [276]

Noguchi et al. [276] introduced a hybrid reconstruction framework integrating a vector quantized variational autoencoder (VQVAE) with a pixel convolutional neural network (PixelCNN), as illustrated in Fig 44. In this approach, VQVAE is employed for microstructural characterization, extracting key morphological features from digital images. Meanwhile, PixelCNN captures the stochastic relationship between process parameters and material properties, enabling accurate and data-driven microstructure synthesis. The variational autoencoder (VAE) utilizes continuous latent vectors, which are assumed to follow a Gaussian distribution. In contrast, the vector quantized variational autoencoder (VQVAE) replaces continuous latent vectors with discrete ones through an additional vector quantization (VQ) process. While VQVAE retains the convolutional encoder-decoder structure of VAE, it introduces the VQ mechanism to enforce discrete latent representations.

Similar to the SINN-based reconstruction method, PixelCNN is used to model the joint distribution of features extracted by VQVAE under conditional distributions:

44 $$\begin{aligned} P\left( {\boldsymbol{x}}\big \vert {\boldsymbol{h}}\right) = \sum _{i=1}^{n} P\left( x_i\big \vert x_1,...,x_{i-1} ,{\boldsymbol{h}}\right)\,, \end{aligned}$$

where ${\boldsymbol{x}}$ represents the input image, $x_i$ is a single pixel in the input image, and ${\boldsymbol{h}}$ denotes the given condition. With the trained VQVAE encoder, each pixel can take one of *K* discrete values, corresponding to the size of the codebook. The model outputs the probability distribution over these *K* values for each pixel, based on dependencies within the image. Once trained, the network effectively captures microstructure patterns in the latent space, representing the image as a conditional distribution of index lists following a sequential order—from left to right and top to bottom. This VQVAE-PixelCNN-based reconstruction method has been applied to steel microstructures, yielding qualitatively satisfactory agreement with experimental results.

#### WGAN-CNN-Based Reconstruction

**Fig. 45:**
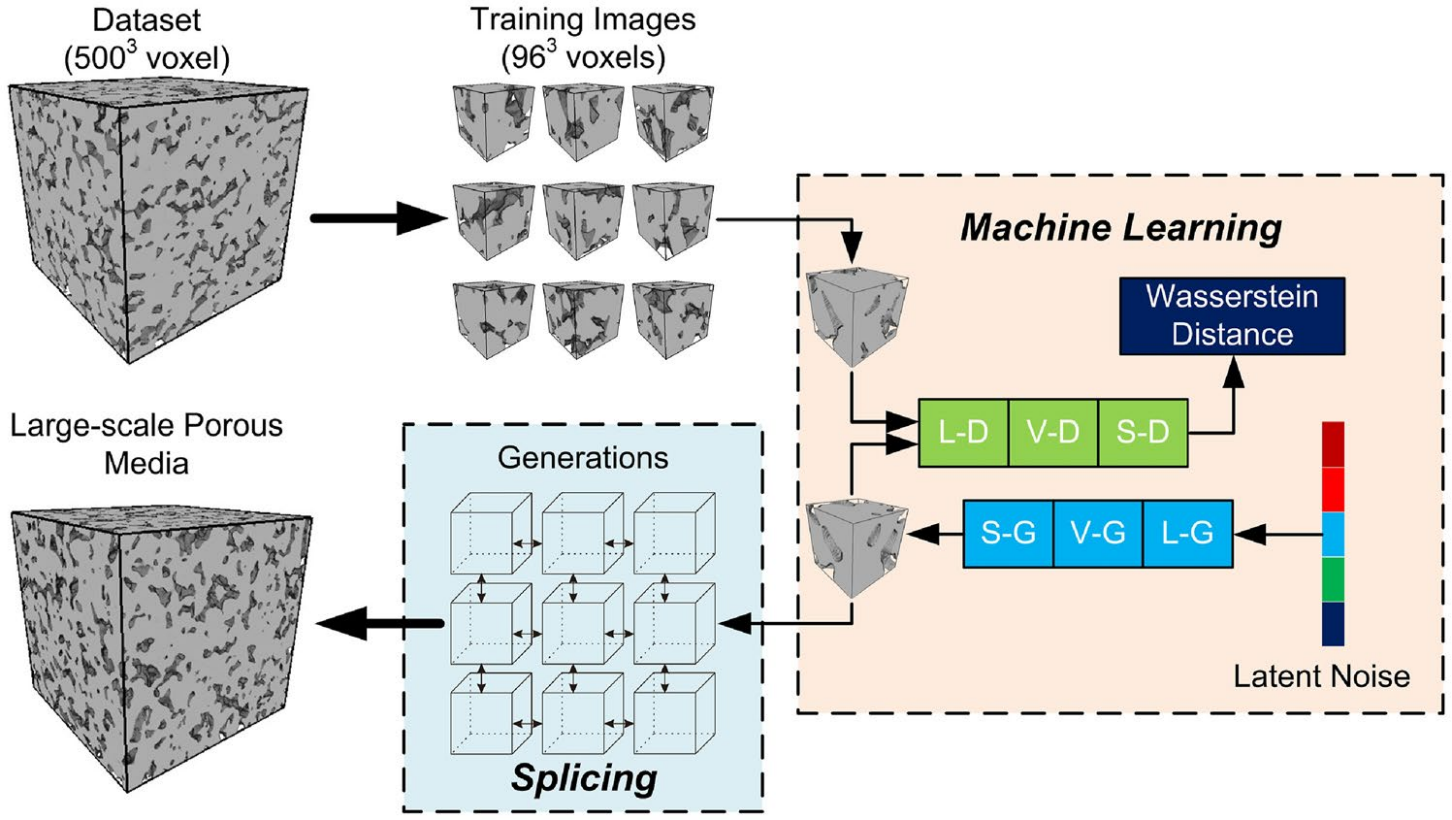
The workflow of the WGAN-CNN-based method for stochastic microstructure reconstruction [277]

To address computational limitations in reconstructing large-scale microstructure samples, Zhang et al. [277] proposed a hybrid WGAN-CNN approach for stochastic reconstruction of 3D random porous microstructures. Inspired by image splicing, this WGAN-CNN-based method divides the reconstruction process into two key stages: small-scale porous media reconstruction and large-scale splicing, where smaller microstructures are assembled into a coherent large-scale representation, as illustrated in Fig 45.

In the first stage, small-scale porous microstructures are generated with partially predefined surfaces and edges, utilizing three generators composed of four different networks: L-WGAN generates 2D porous media surfaces while ensuring edge continuity; V-GAN reconstructs the central porous body; Finally, S-WGAN and S-CNN are combined to generate buffer regions while maintaining boundary continuity. The second stage involves connecting small-scale reconstructed microstructures to form large-scale porous media sample. This process follows a structured generation sequence: Edge-ended blocks are generated first using real porous media data; Edge-centered blocks are then synthesized, ensuring continuity with the previously generated edge-ended blocks; Surface-centered blocks follow, incorporating two unconstrained random surfaces and four constrained surfaces; Finally, body-centered blocks are generated, ensuring consistency across all constrained surfaces. While the splicing process bears similarities to sequential reconstruction, it is included here due to its integration with generative models. This WGAN-CNN-based approach effectively balances computational efficiency with large-scale reconstruction accuracy.

#### GAN-AC-Based Reconstruction

**Fig. 46:**
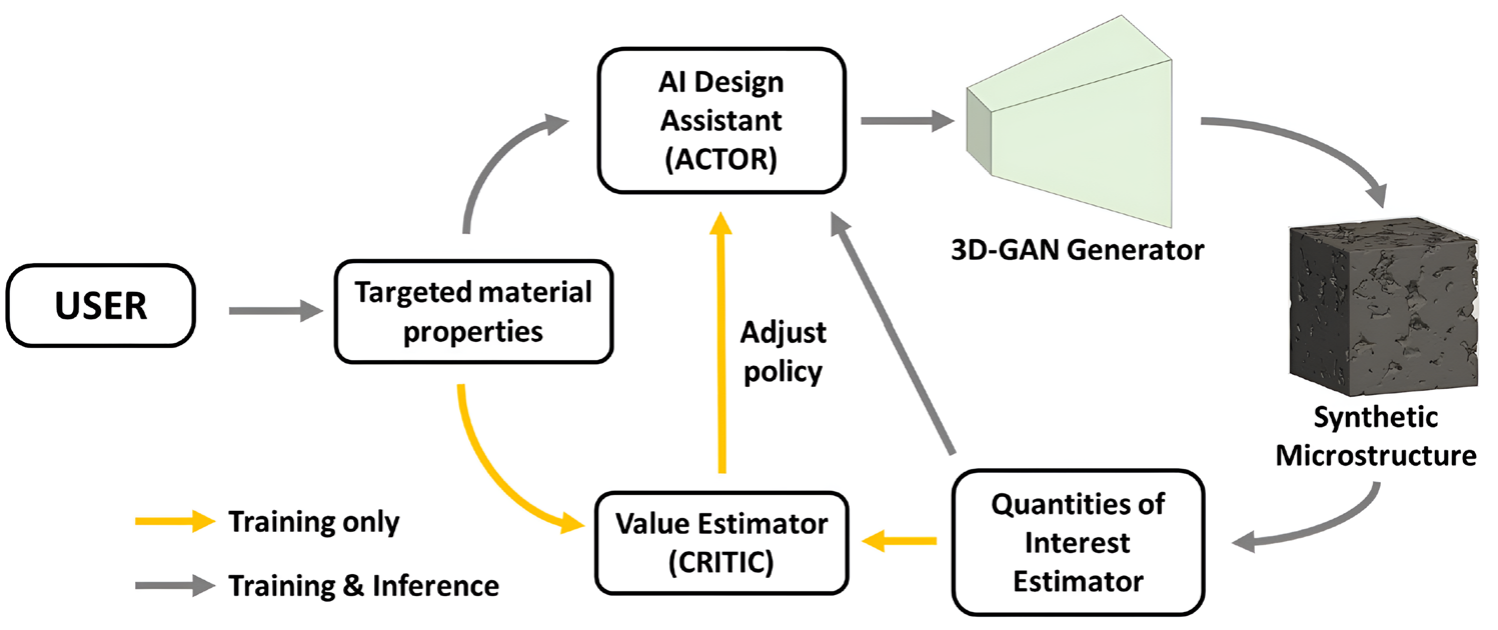
The workflow of the GAN-AC-based method for stochastic microstructure reconstruction [279]

Nguyen et al. [279] integrated actor-critic (AC) reinforcement learning into a GAN-based reconstruction framework to fine-tune the morphological characteristics of reconstructed microstructure samples. The workflow of this GAN-AC-based reconstruction method is illustrated in Fig 46. In this approach, an AI design assistant (actor network) adjusts the input parameters of the 3D-GAN model to achieve target physical properties. The training process consists of two stages: The 3D-GAN model is trained for synthetic microstructure reconstructions in the first step. Then the AC model is trained to generate morphology parameters for the generator based on the given targeted properties, which accurately assess the quality of synthetic microstructures. The AC model iteratively refines the generator’s inputs, ensuring that the reconstructed microstructures closely match the desired physical properties. Quantitative validation demonstrates strong agreement between the target and reconstructed microstructures in terms of porosity, specific surface area, and effective permeability.

#### CGAN-TSS-Based Reconstruction

**Fig. 47:**
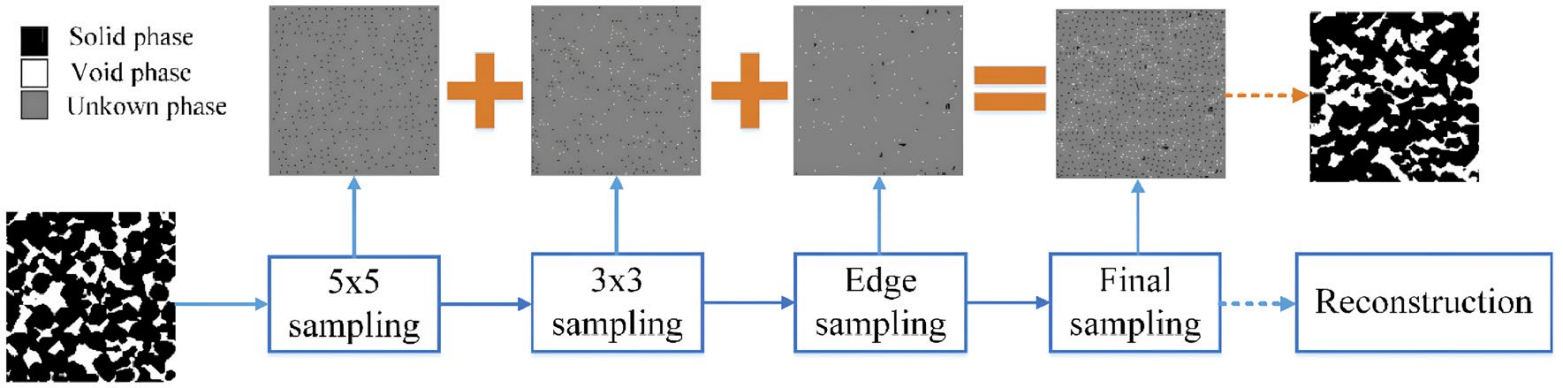
The framework of the CGAN-TSS-based method for stochastic microstructure reconstruction [237]

Another hybrid reconstruction approach combines traditional methods with machine learning (ML) or deep learning (DL) techniques. Feng et al. [237] proposed a method that integrates conditional Generative Adversarial Networks (CGAN) with the three-step sampling (TSS) method, a type of multi-point statistics (MPS)-based approach. The TSS method consists of three sequential steps: $5\times 5$ sampling, $3\times 3$ sampling and edge sampling, as illustrated in Fig 47. Unlike conventional CGANs, which take random noise as input, this method utilizes the final sampling points (that encode two-point correlation function information) as input to the CGAN. By incorporating MPS-based techniques, this hybrid approach accelerates dimensionally upgrade reconstruction while requiring only a limited number of training images.

#### ST-CGAN-Based Reconstruction

**Fig. 48:**
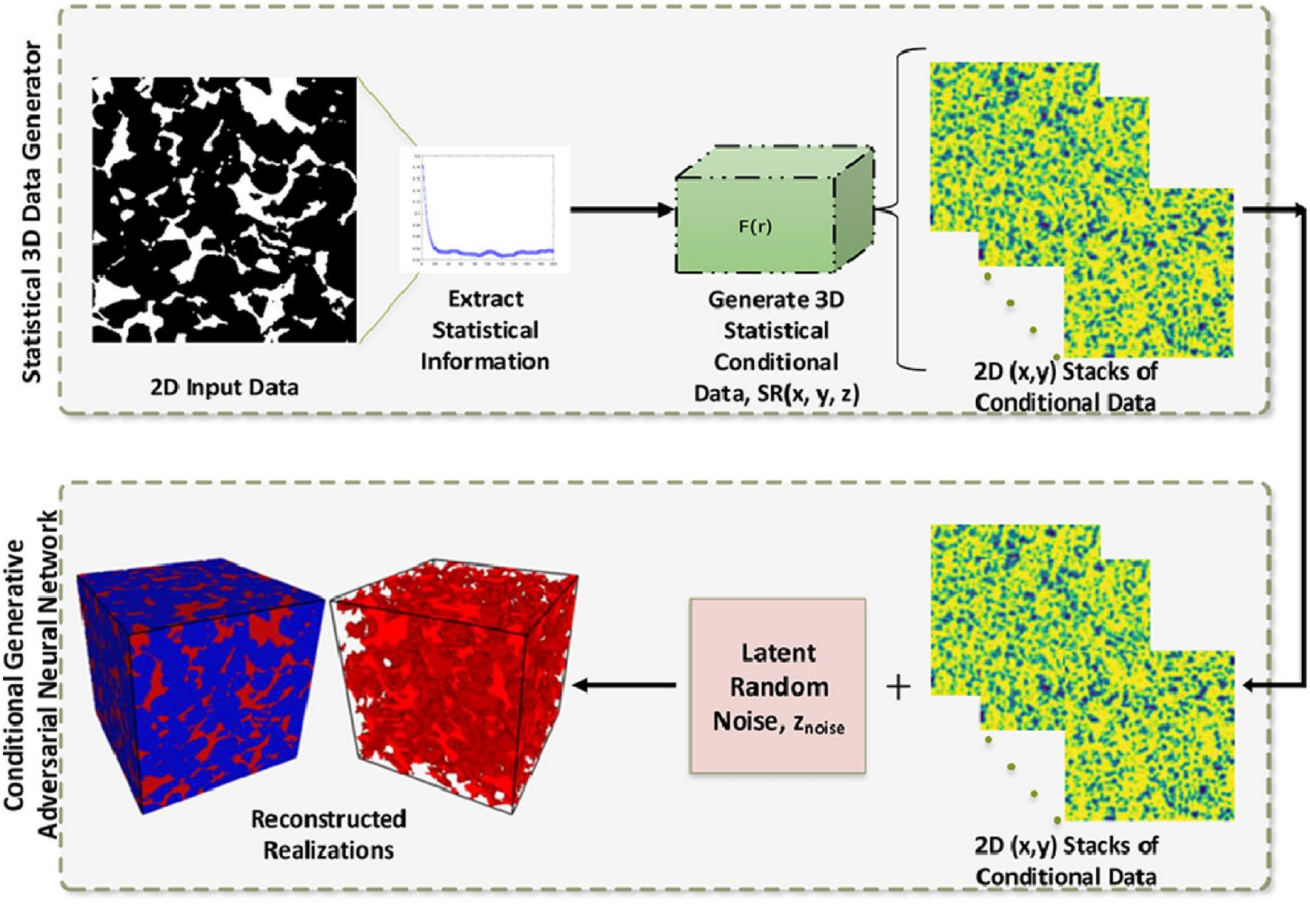
The framework of the ST-CGAN-based method for stochastic microstructure reconstruction [236]

Shams et al. [236] introduced a hybrid statistical and conditional generative adversarial network (ST-CGAN) for the 3D reconstruction of both homogeneous and heterogeneous porous media from a single 2D image. The approach first applies a statistical method to provide conditional data, which is then used to train the generative network. The conditional nature of the model ensures network stability and convergence, which is further optimized through gradient-descent learning. This hybrid approach is particularly effective for reconstructing heterogeneous samples, which have been difficult to achieve with traditional methods. The primary contribution of this work is the development of an adaptable framework that can efficiently reconstruct heterogeneous porous media using CGANs, as shown in Fig 48. The approach also offers a 1000-fold speedup compared to traditional statistical reconstruction methods. The model’s performance is assessed using both morphological and physical matching criteria. The reconstructed results are compared to models produced by a conventional 3D GAN and a widely recognized statistical method, with the findings confirming that this framework provides a reliable and efficient solution for extracting 3D microstructures from a single 2D image.

### Merits and Limitations

Computational intelligence-based reconstruction methods, leveraging machine learning (ML) and deep learning (DL), have significantly enhanced the characterization and synthesis of random porous microstructures. Unlike traditional algorithm-based methods that rely on explicitly defined statistical descriptors or models, DL-based approaches autonomously extract complex hierarchical features from digital image data, enabling a more flexible and efficient reconstruction process. One of the key advantages of ML/DL-based methods is their strong ability to learn high-order statistical dependencies and hidden patterns from training datasets, which are often too intricate to be explicitly formulated using traditional algorithms. This allows for stochastic reconstruction of porous microstructures with detail-similar morphology, which accurately preserves pore geometry, topological features, and transport-relevant characteristics. Besides, DL-based approaches, particularly generative models such as GANs, VAEs and diffusion models, enable the rapid generation of diverse, statistically equivalent microstructures while maintaining scalability and efficiency.

Another crucial advantage of AI-driven methods is their reusability and computational efficiency after training. Once a ML/DL model has been properly trained on a dataset, it can be stored and used to generate an unlimited number of microstructural realizations within seconds to minutes, without the need for iterative optimization. This contrasts with traditional stochastic optimization-based methods, which often involve computationally intensive iterative updates. Furthermore, ML/DL models can be combined with multi-resolution or multi-scale strategies, allowing microstructure reconstructions at different length scales to be seamlessly integrated, making them well-suited for hierarchical porous materials. These models also offer adaptability, as they can be trained on a diverse range of microstructures, including isotropic, anisotropic, and multi-phase porous media. This adaptability makes AI-driven methods highly effective for applications in materials informatics, digital rock physics, and computational materials science.

However, despite the above mentioned advantages, computational intelligence-based reconstruction methods also come with significant limitations. One of the most critical challenges is low interpretability and explainability. Deep learning models often function as black boxes, meaning that it is difficult to understand what specific morphological features are being learned and embedded into the generated microstructure samples. This lack of transparency raises concerns regarding validation, generalization, and physical realism, particularly when dealing with porous materials where physical constraints must be strictly preserved. Unlike physics-aware methods that explicitly incorporate physical constraints into AI models, data-driven ML/DL-based reconstruction methods rely on learned morphological patterns, which may not always guarantee physically meaningful outcomes.

Data dependency is a major drawback, because the effectiveness of ML/DL-based reconstruction largely depends on the quality and quantity of training data. Many DL models require large, high-quality 3D digital images to learn representative microstructural features, which contradicts one of the primary motivations for stochastic reconstruction, i.e., generating new microstructure samples from minimal data. When only a small number of training samples are available, models risk overfitting, producing artifacts that do not generalize well to unseen microstructures. However, it should be noted that several approaches stand out for their ability to perform well with limited training data. For examples, SINN-based methods [51, 82] have demonstrated the ability to perform robust stochastic reconstruction of porous microstructures using only a few training images. StyleGAN-based reconstruction [245, 247] employs adaptive discriminator augmentation mechanism, enabling effective training with limited data. Transfer learning-based reconstruction [105] leverages pretrained models and adapt them to the target domain using only a small number of samples, thus significantly reducing data requirements. SinGAN model [252] is uniquely designed to be trained on a single image, using multi-scale learning of internal patch statistics. These methods are particularly advantageous in practical scenarios where acquiring a large collection of labeled microstructure images is expensive or infeasible.

In addition, another challenge is the lack of precise control over generated microstructure samples. Unlike traditional algorithmic methods where specific statistical descriptors can be explicitly set as reconstruction targets, ML/DL-based reconstruction methods often lack direct tunability, requiring hyperparameter adjustments or conditional generative models (such as CGANs and VAEs) to achieve desired microstructural characteristics. Moreover, DL models typically require extensive computational resources for training, particularly for high-resolution 3D microstructure reconstructions. This includes powerful GPUs or specialized hardware such as TPUs, making these methods less accessible for researchers without high-performance computing (HPC) infrastructure.

## Validation of Microstructure Reconstruction

Validating reconstruction results is essential to ensure that the generated samples accurately replicate the statistical, morphological and physical characteristics of the real microstructures. Since microstructural characteristics of random porous media directly influence effective macroscopic properties, rigorous validation is required to assess their reliability for scientific and engineering applications. A comprehensive validation should ensure that the synthetic microstructures not only resemble real samples visually but also exhibit equivalent functional properties [210]. Therefore, reconstruction validation for random microstructures are broadly categorized into two key aspects:

- **Microstructural morphology** validation via quantitative image analysis;
- **Physical property** validation via image-based poro/micro-mechanical modeling.

### Microstructural Morphology Validation

**Fig. 49:**
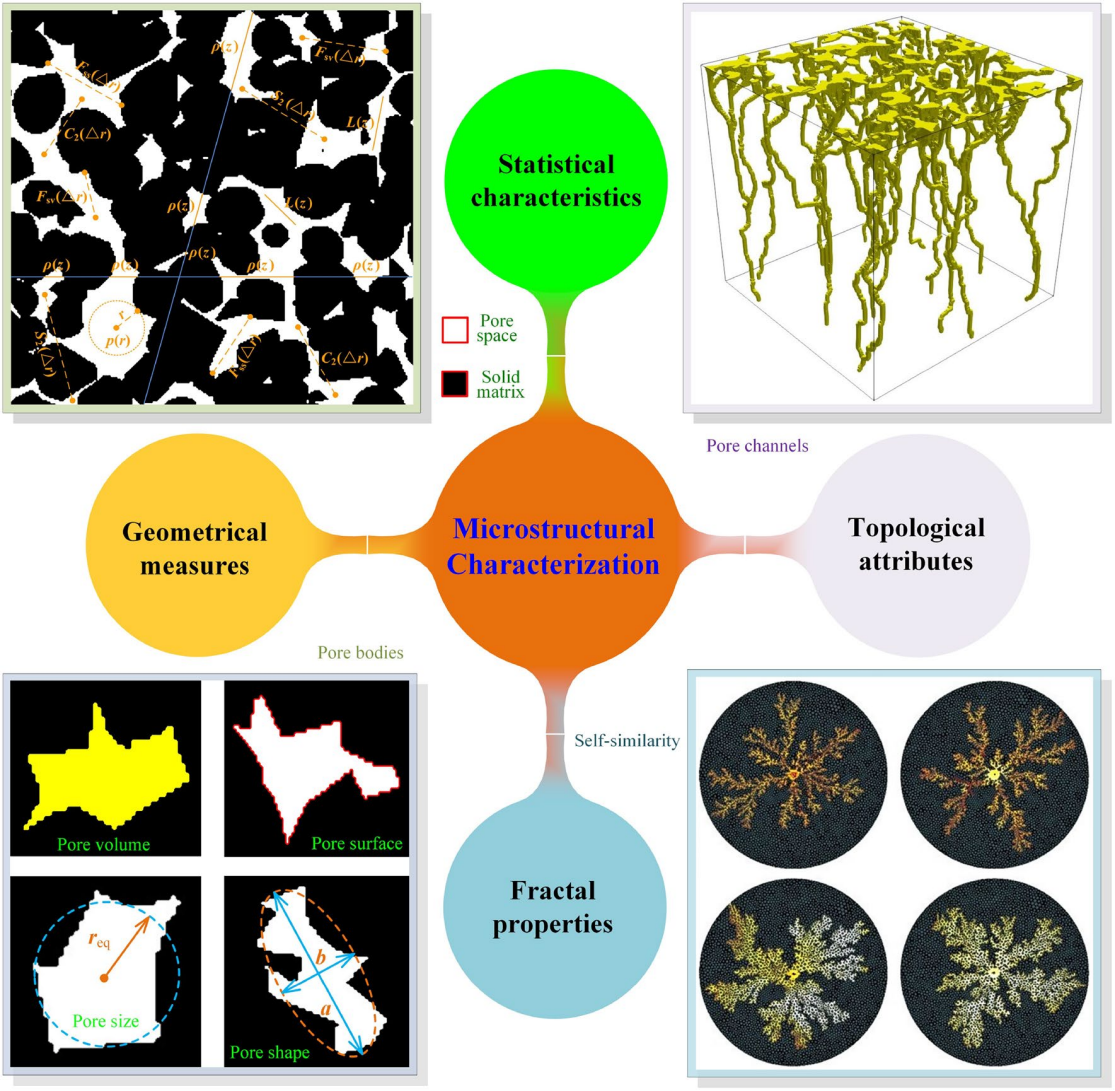
Quantitative microstructural characterization of random porous media from from four key perspectives: statistical characteristics, geometrical measures, topological attributes, and fractal metrics

Microstructural morphology defines the internal architectures of porous media and plays a crucial role in determining their physical behaviors, such as fluid transport, mechanical response, and thermal conduction. To ensure that reconstructed microstructure samples accurately capture the morphological features of real materials, morphology validation should involve a comprehensive quantitative analysis from four key perspectives: statistical characteristics, geometrical measures, topological attributes, and fractal metrics, as illustrated in Fig 49.

#### Statistical Characteristics

Statistical descriptors [85, 107, 296] provide quantitative measures of microstructural randomness, heterogeneity, and phase distributions. These descriptors ensure that reconstructed microstructures maintain statistical equivalence with real samples, enabling accurate predictions of physical properties. The most commonly used statistical descriptors include:

- Two-point correlation function $S_2(\textbf{r})$ [107, 297]: Measures the probability that two points separated by a length vector $\textbf{r}$ belong to the same phase, capturing spatial phase distributions. It is defined as:
  45 $$\begin{aligned} S_2(\textbf{r}) = \frac{1}{V} \int _V I(\textbf{x}) I(\textbf{x}+\textbf{r}) \textrm{d}\textbf{x}\,, \end{aligned}$$
  where $I(\textbf{x})$ is the phase indicator function.
- Lineal path function $L(\textbf{r})$ [296, 298]: Assesses the probability of encountering a continuous phase along a straight path of length $\textbf{r}$, reflecting connectivity. It is particularly useful for evaluating transport properties in porous media.
- Two-point cluster correlation function $C_2(\textbf{r})$ [107, 299]: Evaluates whether two points belong to the same connected cluster, providing insights into percolation and phase continuity. This cluster correlation function is essential for predicting the percolation threshold in porous materials.
- Chord length distribution $\rho (z)$ [107, 300]: Provides a probability distribution of chord lengths, revealing characteristic pore and grain sizes. It is defined as the probability density function of the chord length $z$ measured along a random line through the microstructure.

By comparing these statistical descriptors between real and reconstructed microstructures, the fidelity of the synthetic microstructures can be rigorously evaluated. A high degree of statistical consistency indicates that the generated samples effectively capture the essential characteristics of the original material. This suggests that their derived physical properties can be statistically equivalent, ensuring the reliability of the reconstructed microstructure samples for scientific and engineering applications.

#### Geometrical Measures

Geometrical measures [12, 30, 296] characterize the size, shape, and spatial distributions of pore and solid phases, playing a fundamental role in transport, mechanical, and reactive properties of porous media. These metrics provide insights into pore geometry, which are essential for evaluating fluid dynamics, permeability, and mechanical strength. The key geometrical measures include:

- Porosity ($\phi$) [14, 85]: Represents the volume fraction of pore space within a given volume. It is a fundamental measure that governs transport and mechanical properties:
  46 $$\begin{aligned} \phi = \frac{V_{\textrm{pores}}}{V_{\textrm{total}}}\,, \end{aligned}$$
  where $V_{\textrm{pores}}$ is the volume of pore space, and $V_{\textrm{total}}$ is the total volume of the material. Higher porosity generally enhances permeability but may reduce mechanical strength.
- Pore size distribution $P(d)$ [301, 302] characterizes the relative abundance of pores of different sizes within a porous medium. It quantifies how the volume of the pore space changes with respect to the pore diameter $d$, defined as:
  47 $$\begin{aligned} P(d) = -\frac{\textrm{d}F(d)}{\textrm{d}d}\,, \end{aligned}$$
  where $S(d)$ represents the remaining pore space fraction after applying a morphological opening with a spherical structuring element of diameter $d$. Commonly derived using *morphological granulometry* or *distance transform* methods. It should be noted that the definition of a “pore” and its associated size can vary depending on the physical process of interest, which introduces a degree of non-uniqueness in how PSD is defined and applied.
- Specific surface area ($SSA$) [14, 303]: Defined as the total interfacial surface area per unit volume, SSA directly influences transport efficiency, reaction kinetics, and adsorption capacity. It is mathematically expressed as:
  48 $$\begin{aligned} SSA = \frac{S}{V_{\textrm{total}}}\,, \end{aligned}$$
  where $S$ is the total surface area of the pore-solid interface. A higher SSA typically indicates greater complexity of the pore microstructure, leading to enhanced diffusion and reaction rates.
- Shape factor ($SF$) [304, 305]: Quantifies the deviation of pore or grain shapes from ideal geometries. It is defined as:
  49 $$\begin{aligned} SF = \frac{P^2}{4\pi A}\,, \end{aligned}$$
  where $P$ is the perimeter, and $A$ is the cross-sectional area. A shape factor close to 1 indicates a near-circular shape, whereas larger values signify more irregular morphologies, which can impact mechanical stability and flow dynamics.
- Mean surface curvature ($H$) [12, 30]: Represents the local curvature of pore-solid interfaces, which influences capillary-driven transport, mechanical stability, and phase interactions. It is computed as:
  50 $$\begin{aligned} H = \frac{1}{2} (\kappa _1 + \kappa _2)\,, \end{aligned}$$
  where $\kappa _1$ and $\kappa _2$ are the principal curvatures of the surface. Higher curvature values indicate sharper interface variations, which can enhance fluid retention and affect stress concentration within the solid matrix.

By comparing these geometrical measures between real and reconstructed microstructures, the accuracy and representativeness of the generated samples can be rigorously assessed. A high degree of agreement ensures that the synthetic microstructure samples will exhibit realistic transport and mechanical behaviors.

#### Topological Attributes

Topological attributes [306–308] provide insights into interconnectivity and percolation of pore networks within porous media, which are crucial for fluid transport and mass diffusion. The most commonly used topological descriptors include:

- Euler characteristic ($\chi$) [12, 30]: Quantitatively measures pore connectivity and distinguishes between isolated pores and interconnected pathways. It is defined as:
  51 $$\begin{aligned} \chi = C - H\,, \end{aligned}$$
  where $C$ represents the number of connected components, and $H$ denotes the number of enclosed cavities. A higher Euler characteristic suggests a more fragmented microstructure, while a lower value indicates a well-connected porous network.
- Betti numbers ($\beta _0, \beta _1, \beta _2$) [309, 310]: These algebraic topology invariants describe the number of independent topological features:
  - $\beta _0$: Number of connected components;
  - $\beta _1$: Number of independent loops or circular voids, which influence transport pathways;
  - $\beta _2$: Number of fully enclosed voids or cavities within the material;
  The combination of three numbers provides a quantitative fingerprint of microstructural complexity.
- Pore network connectivity [30, 311]: Connectivity is assessed using percolation theory, which determines whether a continuous pathway spans the entire microstructure. The percolation threshold, the critical porosity at which a spanning cluster forms,plays a vital role in predicting transport properties.
- Coordination number ($Z$) [312, 313]: Represents the average number of direct connections per pore, which is mathematically defined as:
  52 $$\begin{aligned} Z = \frac{\sum _{i=1}^{N} Z_i}{N}\,, \end{aligned}$$
  where $Z_i$ is the number of neighboring pores connected to the $i$th pore, and $N$ is the total number of pores. A higher coordination number indicates enhanced transport efficiency.
- Total fraction of percolating cells ($T_{\alpha }(L)$) [314, 315]: Quantifies the proportion of pore cells that form a continuous percolating network, which is critical for assessing large-scale transport properties, such as permeability and electrical conductivity.
- Tortuosity ($\tau$) [308]: Defines the sinuosity and complexity of transport pathways inside porous media, impacting fluid flow, ion diffusion, and electrical conductivity. It is given by:
  53 $$\begin{aligned} \tau = \frac{L_{\textrm{eff}}}{L}\,, \end{aligned}$$
  where $L_{\textrm{eff}}$ is the actual transport path length, and $L$ is the straight-line distance between two points. A higher tortuosity value suggests more convoluted pathways, increasing resistance to flow and diffusion.

#### Fractal Metrics

Fractal metrics [36, 316, 317] describe the complexity, self-similarity, and heterogeneity of porous architectures across multiple length scales. These metrics are particularly useful for characterizing hierarchical and disordered porous media, where traditional Euclidean geometry fails to capture intricate pore structures. Common fractal metrics include:

- Fractal dimension ($D_f$) [316, 318]: Quantifies how the structural complexity of a porous medium changes with scale. It provides insight into the space-filling capacity of the microstructure and is typically determined using box-counting or Minkowski-Bouligand methods. It is mathematically defined as:
  54 $$\begin{aligned} D_f = \lim _{r \rightarrow 0} \frac{\log N(r )}{\log (1/r)}\,, \end{aligned}$$
  where $N(r )$ is the number of boxes of size $r$ required to cover the entire sample. A higher $D_f$ suggests a more intricate and space-filling structure, which is often associated with enhanced transport properties and mechanical strength.
- Lacunarity ($\Lambda(r)$) [36, 319]: Measures the degree of spatial heterogeneity in a porous medium by evaluating the distribution of void sizes. It provides complementary information to fractal dimension, capturing structural gaps and clustering effects. It is commonly computed using the gliding box algorithm or power-law scaling analysis.
  55 $$\begin{aligned} \Lambda(r) = \frac{\sigma ^2(r)}{\mu ^2(r)}\,, \end{aligned}$$
  where $\sigma ^2(r)$ and $\mu ^2(r)$ are the variance and mean of the pore distribution at scale $r$, respectively. A higher lacunarity value indicates greater heterogeneity and variability in pore sizes, which can influence fluid retention, permeability, and mechanical strength.
- Succolarity [320, 321]: Assesses the degree of connectedness and accessibility of pore networks, reflecting how effectively fluid or mass can traverse porous structures. It is particularly useful in transport phenomena, such as permeability and diffusion, where connectivity plays a crucial role. Succolarity is often computed by analyzing the shortest path of fluid percolation or by evaluating the pore network’s largest connected component. A higher succolarity value signifies better transport efficiency and increased permeability, making it a critical metric for characterizing highly connected porous media.

### Physical Property Validation

**Fig. 50:**
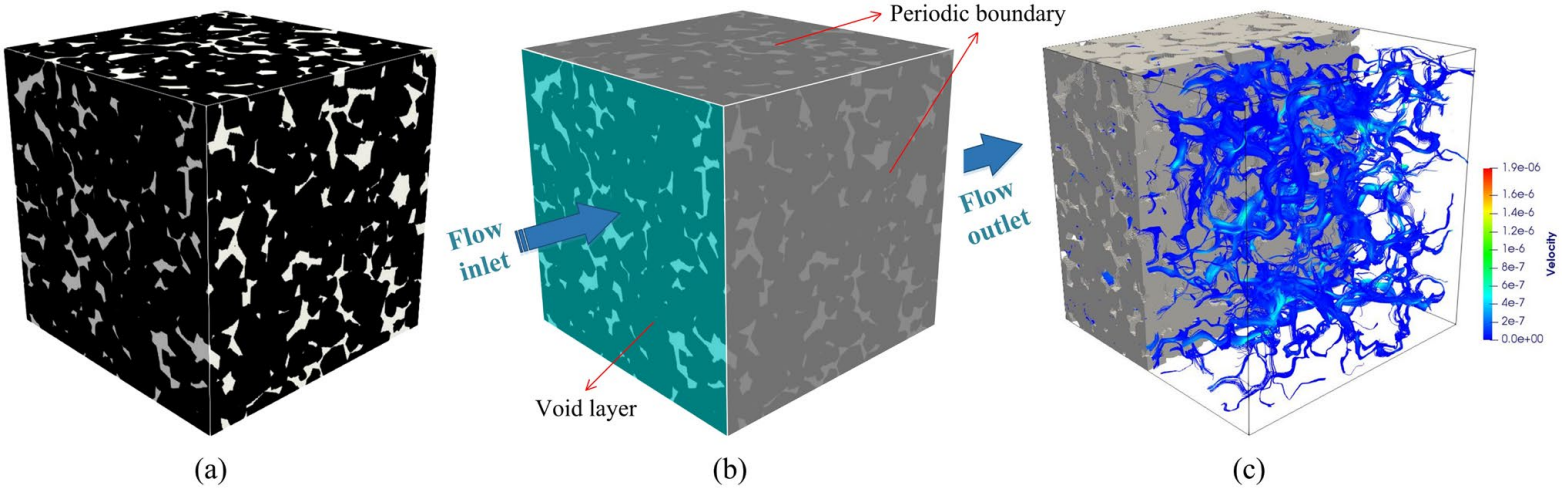
Numerical evaluation of absolute permeability using LBM: (**a**) The 3D digital microstructure of a porous medium (white regions represent pore space, and black regions represent solid phases); (**b**) Boundary condition settings for numerical simulation; and (**c**) The steady-state fluid velocity field obtained from image-based pore-scale modeling [14]

**Fig. 51:**
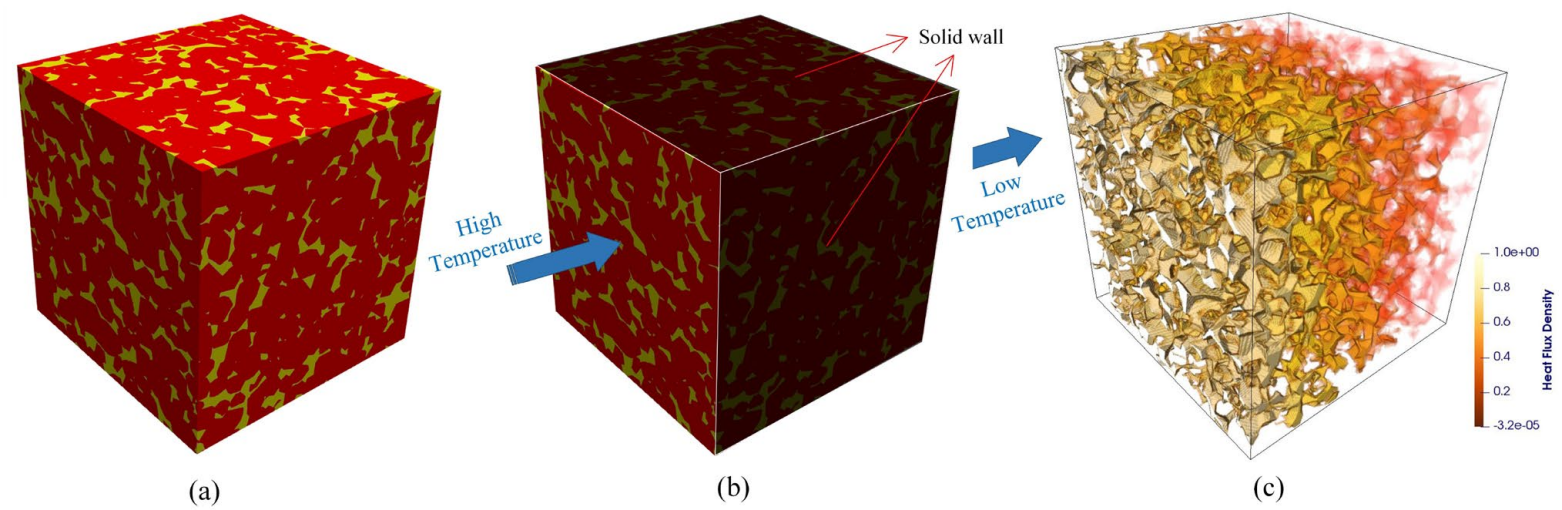
Numerical evaluation of thermal conductivity using voxel-based FVM: (**a**) The 3D digital microstructure of a porous medium (yellow regions represent pore space, and red regions represent solid phases); (**b**) Boundary condition settings for numerical simulation; and (**c**) The steady-state heat flux density obtained from image-based pore-scale modeling [308]

**Fig. 52:**
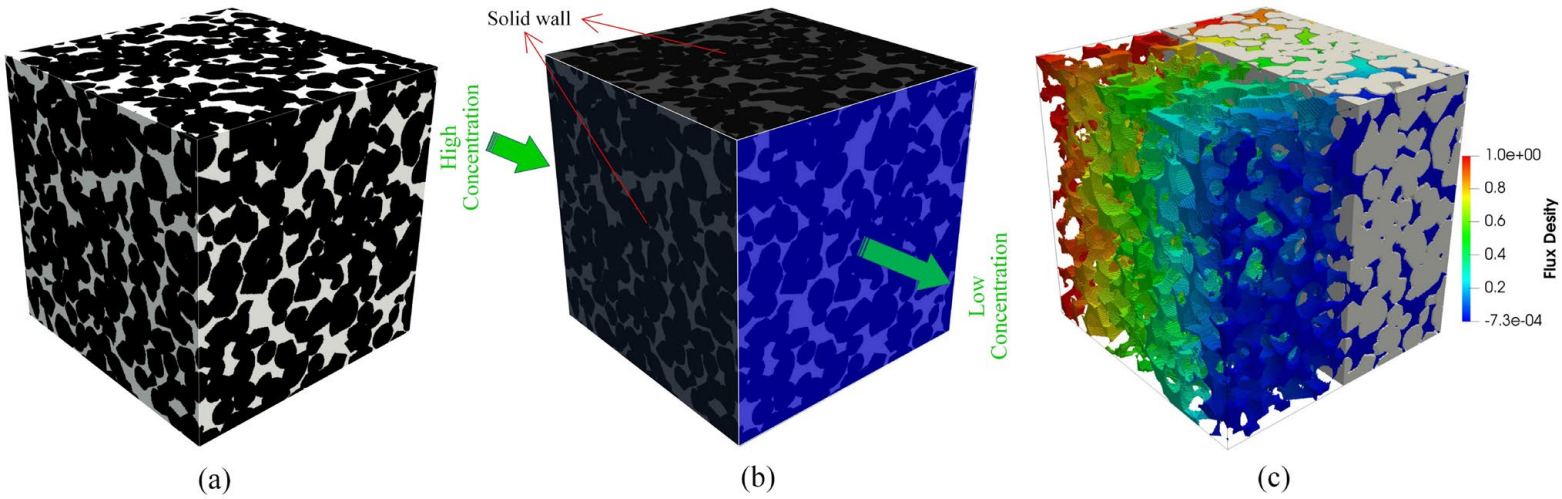
Numerical evaluation of effective diffusivity using FDM: (**a**) The 3D digital microstructure of a porous medium (white regions represent pore space, and black regions represent solid phases); (**b**) Boundary condition settings for numerical simulation; and (**c**) The steady-state diffusional flux density obtained from image-based pore-scale modeling [192]

**Fig. 53:**
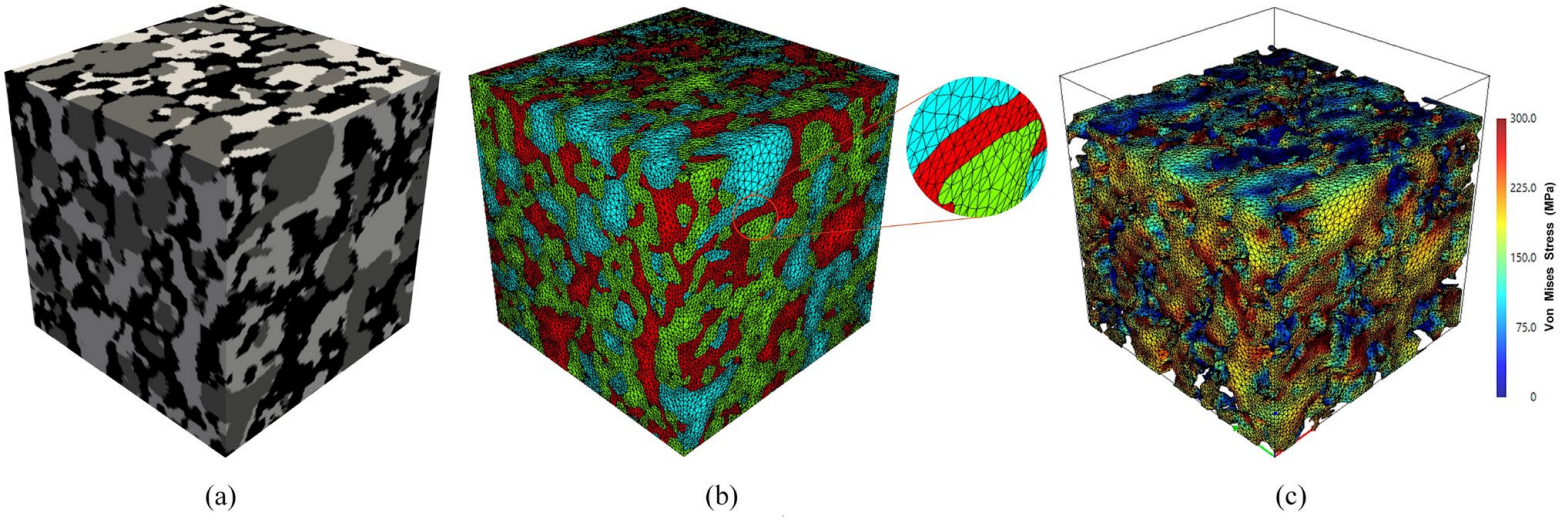
Numerical evaluation of effective Young’s modulus and compressive strength using FEM: (**a**) The 3D microstructure of a porous SOFC anode (white is YSZ, grey is Ni, and black is pore); (**b**) Image-based meshing of the multiphase microstructure for numerical simulation; and (**c**) The Von Mises stress field under axial compression obtained from image-based micro-mechanical modeling [51]

**Fig. 54:**
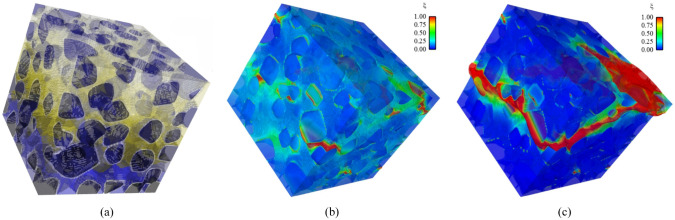
Numerical evaluation of fracture toughness and energy release rate using phase field method: (**a**) The 3D microstructure of a porous ceramic composite; (**b**) The fracture order parameter $\xi$ obtained from the phase field model when the normal strain $\bar{\varepsilon }$ reaches 0.048; and (**c**) The fracture order parameter $\xi$ when the normal strain $\bar{\varepsilon }$ reaches 0.060 [322]

Accurate validation of physical properties is crucial to ensuring that reconstructed microstructure samples can reliably replicate the behavior of real porous materials in practical applications. Physical property validation typically focuses on transport and mechanical properties, which govern key material performance characteristics such as permeability, thermal conductivity, effective diffusivity, mechanical strength, and failure behaviors. A well-validated reconstruction method should not only match statistical descriptors and morphological features but also demonstrate equivalent physical responses under various conditions. Various numerical techniques, such as the Lattice Boltzmann method (LBM) [14, 57], Finite difference method (FDM) [58], Finite volume method (FVM) [59], and Finite element method (FEM) [55, 56], are commonly employed in microstructure-based simulations, as shown in Figs 50, 51, 52, 53, 54. These numerical simulations help ensure that the reconstructed microstructures are not only visually similar but also functionally equivalent to their real counterparts.

It should be noted that each numerical simulation method has distinct characteristics that make it more or less suitable for specific applications. LBM and FDM operate naturally on regular voxel grids, making them highly compatible with image-based simulations of fluid flow and transport phenomena. These methods are typically easier to implement but may be limited in handling complex boundary conditions or material heterogeneity. In contrast, FEM and FVM generally require an additional meshing step to discretize the domain, which increases preprocessing effort but offers greater geometric flexibility and higher accuracy, especially in solid mechanics and multi-physics simulations. FEM is particularly well-suited for simulating mechanical deformation, stress distribution, and elasticity in heterogeneous materials. Computational demand tends to be higher for FEM and FVM compared to LBM or FDM, especially when working with unstructured meshes. Additionally, Pore network modeling (PNM) [323–325] is a simplified and computationally efficient approach frequently used in applied single-phase or multi-phase flow simulations. Although it is less rigorous and relies on idealized representations of the pore structure (e.g., pores as spheres and throats as cylinders), PNM offers a valuable tool for upscaled analysis, especially in large-scale porous media where direct numerical simulations are computationally prohibitive.

#### Transport Properties

Transport properties play a critical role in determining the efficiency of fluid permeation, mass diffusion, heat transfer, and electrical conduction within porous media. Validating these properties ensures that the reconstructed microstructures accurately replicate the real material’s transport behavior. Key validation metrics include:

- Permeability ($\kappa$) [14, 326]: Quantifies the ability of a porous medium to transmit fluid under a pressure gradient. It is commonly evaluated using Darcy’s law and is computed through numerical methods such as LBM, FEM or PNM (as illustrated in Fig 50):
  56 $$\begin{aligned} \kappa = \frac{Q \mu L}{A \Delta P}\,, \end{aligned}$$
  where $Q$ is the volumetric flow rate, $\mu$ is the dynamic viscosity of the fluid, $L$ is the sample length, $A$ is the cross-sectional area, and $\Delta P$ is the applied pressure difference. Although a good match in absolute permeability between real and reconstructed microstructures is often used as a validation criterion (particularly in hydrogeology, fuel cells, and filtration), it is fundamentally a bulk-averaged parameter. This means it can be replicated even by geometrically simplified models or empirical relationships (e.g., Carman–Kozeny equation) [14, 326], and may not reflect detailed morphological accuracy. In contrast, relative permeability, which governs multiphase transport, is significantly more sensitive to microstructural features such as pore connectivity, throat constrictions, and wettability. However, relative permeability is also more challenging to measure both experimentally and numerically, and while its non-dimensional form makes general trends easier to replicate, subtle deviations can still lead to meaningful differences in performance predictions.
- Electrical conductivity ($\sigma$) [308, 327]: Describes how efficiently a porous material transmits electric current, which is critical in applications such as battery electrodes and semiconducting foams. It is determined using Ohm’s law:
  57 $$\begin{aligned} \sigma = \frac{I}{V}\,, \end{aligned}$$
  where $I$ is the electrical current, and $V$ is the applied voltage. Computational techniques, such as FDM and FEM, are commonly employed to compare the conductivity of real and synthetic microstructures.
- Thermal conductivity ($\lambda$) [308, 328]: Dictates heat transfer efficiency within a porous medium and is a key property in thermal insulation materials, heat exchangers, and fuel cells. It is evaluated using Fourier’s law (as illustrated in Fig 51):
  58 $$\begin{aligned} q = -\lambda \frac{\textrm{d}T}{\textrm{d}x}\,, \end{aligned}$$
  where $q$ is the heat flux, and $\textrm{d}T/\textrm{d}x$ is the temperature gradient. The reconstructed microstructures should exhibit similar thermal conductivity to real materials to ensure comparable heat dissipation performance.
- Effective diffusivity ($D_{\textrm{eff}}$) [192, 329]: Governs mass transport in porous media and is crucial for applications such as gas diffusion layers in fuel cells and drug delivery systems. It is computed as:
  59 $$\begin{aligned} D_{\textrm{eff}} = D_0 \frac{\phi }{\tau }\,, \end{aligned}$$
  where $D_0$ is the intrinsic diffusivity, $\phi$ is the porosity, and $\tau$ is the tortuosity. Lower tortuosity enhances effective diffusivity, enabling more efficient molecular transport through the pore network. Direct numerical simulations and random walk algorithms are often used to validate diffusion properties, as illustrated in Fig 52.

#### Mechanical Properties

Mechanical properties play a crucial role in determining the structural integrity, load-bearing capacity, and failure behavior of porous materials. These properties are essential for validating whether reconstructed microstructure samples can accurately replicate the mechanical response of real materials. The key mechanical metrics include:

- Young’s modulus ($E$) [51, 156]: Quantifies the stiffness of a porous material, representing the ratio of axial stress to axial strain:
  60 $$\begin{aligned} E = \frac{\sigma }{\varepsilon }\,, \end{aligned}$$
  where $\sigma$ is the applied stress and $\varepsilon$ is the resulting strain. Higher values of *E* indicate stiffer materials with lower deformation under load. FEM simulations are often used to numerically evaluate effective Young’s modulus, as illustrated in Fig 53.
- Compressive strength ($\sigma _c$) [330, 331]: Defines the maximum axial stress that a porous material can withstand before failure under uniaxial compression:
  61 $$\begin{aligned} \sigma _c = \frac{F_{\max }}{A}\,, \end{aligned}$$
  where $F_{\max }$ is the maximum applied load before failure, and $A$ is the cross-sectional area. This property is particularly crucial for porous materials used in load-bearing applications.
- Fracture toughness ($K_{IC}$) [62, 63, 322]: Measures a porous material’s resistance to crack propagation, which is defined by:
  62 $$\begin{aligned} K_{IC} = Y \sigma \sqrt{\pi a}\,, \end{aligned}$$
  where $Y$ is a geometric factor, $\sigma$ is the applied stress, and $a$ is the crack length. A higher $K_{IC}$ indicates a greater level of resistance to fracture, which is critical for porous materials subjected to cyclic loading or impact forces. The phase field method is widely used to numerically simulate fracture propagation inside porous materials, as illustrated in Fig 54.
- Failure modes [332, 333]: The crack initiation and propagation patterns inside porous materials should be analyzed through micro-mechanical modeling. This numerical validation involves assessing:
  - Crack propagation paths and branching behaviors.
  - Energy release rate ($G_c$) to quantify the fracture energy:
    63 $$\begin{aligned} G_c = \frac{1}{E} \left( K_{IC}^2 \right) \,, \end{aligned}$$
  - Strain localization and stress distribution within the porous matrix.

## Discussions on Effective Microstructure Reconstruction

**Fig. 55:**
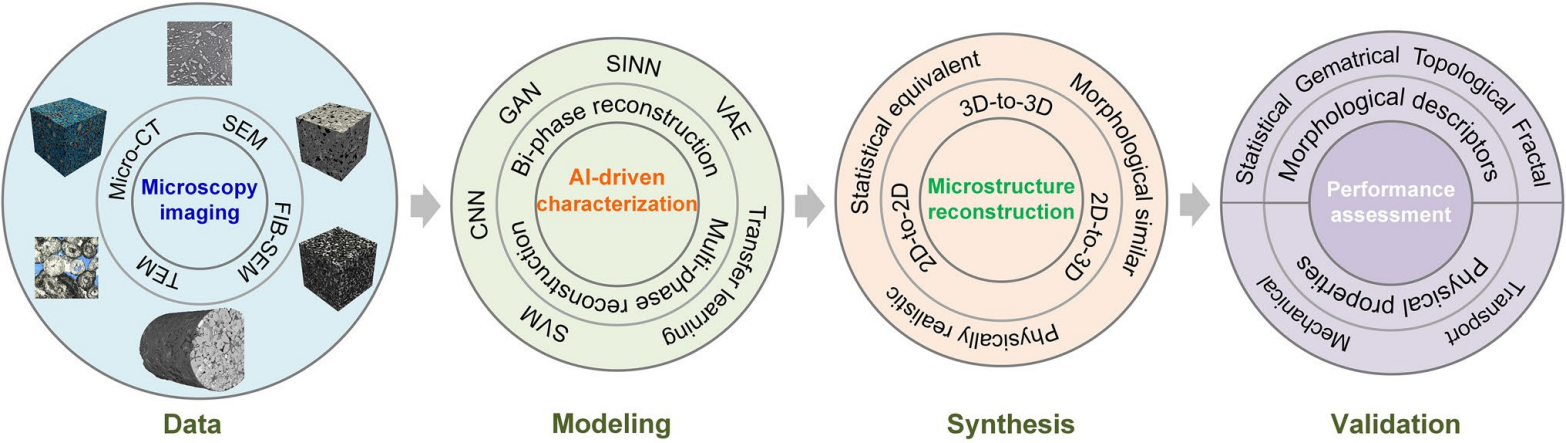
The procedure of develping a computational intelligence-based microstructure reconstruction method

Computational intelligence-based methods for stochastic microstructure reconstruction have gained significant attention and are widely applied across various domains due to their ability to characterize complex porous microstructures and uncover hidden patterns from image datasets. These approaches leverage advanced machine/deep learning algorithms to generate random microstructure samples, offering new possibilities for investigating microstructure-property relationships in porous materials. After detailed discussions in Sections 1-4, the procedure of developing a computational intelligence-based reconstruction method can be structured into four key components: microscopy imaging, AI-driven characterization, microstructure synthesis, and performance validation, as graphically illustrated in Fig 55.

Assessing the effectiveness of microstructure reconstruction techniques requires a rigorous, multi-faceted framework that considers key factors such as data efficiency, reconstruction accuracy, computational feasibility, interpretability, and application relevance. Specifically, an ideal microstructure reconstruction method for random porous media should (i) function effectively even with limited training data, (ii) accurately capture microstructural morphology and physical properties, (iii) maintain computational efficiency for large-scale 3D reconstructions, (iv) offer model interpretability and parameter control, and (v) ensure applicability in engineering and scientific domains. In this section, the key evaluation criteria for assessing the effectiveness of computational intelligence-based methods for stochastic microstructure reconstruction are systematically discussed.

### Training Data Requirement

The effectiveness of machine/deep learning-based microstructure reconstruction is heavily dependent on the quality and quantity of training data. Unlike traditional algorithm-based reconstruction methods that rely on predefined statistical descriptors or models, AI-driven approaches learn microstructural patterns directly from image datasets. This data dependency poses a major challenge in scenarios where only a small number of training samples are available. Many deep learning models require large, high-quality 3D datasets to learn representative microstructural features. This requirement contradicts the motivation behind stochastic reconstruction to generate realistic microstructure samples from minimal data. When training datasets are too small, machine/deep learning models may memorize noise rather than generalizing microstructural patterns, leading to unrealistic microstructure samples that do not match the morphological properties of real porous media. Addressing data scarcity through augmentation techniques (e.g., rotations, noise injection, generative models) and transfer learning can enhance model robustness. However, the challenge remains in ensuring that the augmented data sufficiently captures real microstructural variability. In addition, recent advancements in self-supervised learning and few-shot learning may further alleviate data dependence by enabling AI models to extract meaningful features from smaller datasets.

### Characterization Hierarchy

Random porous media usually exhibit extreme heterogeneity, with interconnected, isolated and dead-end pores forming intricate networks spanning multiple length scales. Accurately characterizing these complexities is a fundamental challenge in microstructure reconstruction, as porous architectures contain features ranging from nanometer-scale pore throats to millimeter-scale grain boundaries. An effective reconstruction method must preserve these hierarchical characteristics to ensure both small-scale morphological details and large-scale connectivity of percolating pathways are faithfully represented. Traditional algorithm-based reconstruction approaches explicitly describe statistical and microstructural characteristics, whereas machine/deep learning-based methods learn hidden representations of morphology patterns. The critical challenge lies in ensuring that the learned representations preserve physically meaningful features rather than irrelevant artifacts. Given the complexity of real porous media, no single method can perfectly capture all microstructural features. A promising approach involves integrating explicit statistical characterization with deep learning-based feature extraction. Hybrid methods combining physical or statistical constraints with deep neural networks may provide enhanced accuracy in characterizing complex pore networks while preserving physically meaningful attributes.

### Reconstruction Accuracy

An effective stochastic reconstruction method should generate microstructure samples that not only look visually similar to real materials but also accurately replicate their geometrical, topological, transport, and mechanical characteristics. Geometrical and topological metrics, such as porosity, specific surface area, shape factor, pore connectivity and tortuosity, should be quantitatively evaluated. Meanwhile, the reconstructed microstructures should exhibit permeability, effective diffusivity, electrical conductivity, or mechanical strength values statistically consistent with these of the real samples. Furthermore, microstructural features at different scales should be captured, ensuring that phase distribution and pore connectivity remain valid across resolutions. In summary, a comprehensive validation should compare the reconstructed microstructures with the real ones in terms of morphological characteristics and physical properties; and ensuring reconstruction accuracy is critical for the digital 3D microstructures to be utilized in scientific and engineering applications, particularly for image-based pore/micro-mechanical modeling.

### Computational Efficiency

The computational cost of stochastic reconstruction increases exponentially with sample size, making large-scale reconstruction of 3D microstructures challenging. Many deep learning models struggle to reconstruct high-resolution 3D microstructure samples (e.g., $512^3$, $1024^3$ or larger) due to GPU memory constraints and prolonged training times. To enhance scalability, efficient architectures such as autoencoders, hierarchical GANs, and implicit neural representations can reduce memory consumption while maintaining fidelity. While some generative models can produce 3D microstructure samples within seconds, others require extensive iterative refinement, necessitating a careful balance between accuracy and efficiency, particularly for real-time applications. Probabilistic models offer an alternative by generating multiple plausible realizations, capturing statistical variability while reducing computational overhead. Strategies such as parallel computing, transfer learning, and lightweight surrogate models further improve computational feasibility. Therefore, optimizing efficiency without compromising reconstruction accuracy is essential for the widespread adoption of AI-driven microstructure reconstruction techniques in scientific and industrial applications.

### Reconstruction Representativeness

In the context of multiscale modeling, the concept of representative volume element (RVE) plays a critical role in bridging microscale features and macroscopic properties. A RVE is defined as the smallest volume over which spatially averaged microstructural properties converge to effective macroscopic quantities, such as permeability or stiffness. However, the definition and identification of a RVE remain challenging, especially in materials with high heterogeneity, anisotropy, or complex pore connectivity. In practice, a volume is often deemed representative if the computed effective properties stabilize with increasing domain size. In practice, the size of RVE is highly dependent on the microstructural or physical property of interest. For example, porosity may stabilize over a much smaller volume than permeability, which is sensitive to pore-scale connectivity and long-range spatial correlations. As such, the definition of the RVE must be carefully contextualized based on the targeted property, the degree of heterogeneity in the microstructure, and the application-specific tolerance for variability. The use of stochastic microstructure reconstruction facilitates the generation of multiple statistically equivalent volume elements, enabling a systematic assessment of representativeness through ensemble averaging. This provides a practical pathway for defining and validating RVEs in image-based poro/micro-mechanical modeling.

For a reconstructed microstructure to be truly representative, it must capture essential statistical and morphological features of real porous media while maintaining structural homogeneity at a sufficiently large scale. If the generated microstructure is too small, it may fail to reflect bulk material properties accurately, leading to inconsistencies in predicted transport or mechanical behaviors. Additionally, many porous materials exhibit large-scale heterogeneity, such as stratified sedimentary layers, fracture networks, or gradient porosity distributions. A robust microstructure reconstruction method should generate diverse realizations that account for these spatial variations rather than merely replicating a single training sample. This is particularly crucial for natural porous media, such as sedimentary rocks, where heterogeneity plays a dominant role in fluid transport and mechanical strength. Consequently, ensuring reconstruction representativeness requires techniques that balance local statistical accuracy with large-scale structural diversity, preventing overfitting while preserving essential heterogeneity.

### Interpretability and Explainability

Enhancing interpretability and explainability in computational intelligence-based microstructure reconstruction is crucial for ensuring reliable and physically meaningful results. Machine/deep learning models often operate as black-box systems, creating difficulties in understanding how random microstructure samples are synthesized. *Interpretability* refers to understanding the relationships between input features and output predictions, while *explainability* concerns how internal parameters and learned representations justify generated results. A lack of interpretability presents several challenges:

- Feature transparency: Identifying which microstructural features are extracted, learned, and retained during the training process is difficult.
- Lack of physical constraints: Data-driven models may struggle to enforce physically consistent reconstructions, potentially leading to unrealistic microstructural geometries or topologies.
- Verification and validation: Without an explicit decision-making process, verifying whether reconstructed microstructure samples statistically reflect the target morphological and physical characteristics requires additional validations.

Investigating how porous microstructures are embedded in the latent space of deep generative models (e.g., GANs, VAEs) can improve transparency and help disentangle meaningful microstructural representations. Furthermore, developing physics-aware machine learning models that integrate domain knowledge into the learning process can ensure physically meaningful reconstructions of random porous microstructures.

### Parameter Controllability

Improving parameter controllability in computational intelligence-based reconstruction methods is essential for generating microstructure samples with desired characteristics. Unlike traditional algorithm-based methods, which allow explicit control over statistical models or descriptors (e.g., porosity, correlation functions), deep learning models often function as black boxes, presenting several challenges:

- Generate microstructures with user-defined features: The absence of explicit input parameters makes it challenging to tailor the morphology to meet specific application requirements.
- Fine-tune reconstruction parameters: Hyperparameters such as latent space vectors, loss functions, and training datasets significantly influence the synthesized microstructures, but their effects are often unclear and difficult to adjust systematically.
- Ensure consistency in reconstructed samples: The stochastic nature of deep generative models (e.g., GANs, VAEs) can lead to unintended randomness, resulting in inconsistencies across generated microstructure samples.

To enhance parameter controllability, future work may focus on conditional generative models (e.g., CGAN, InfoGAN) and reinforcement learning-based optimization frameworks, allowing for user-defined constraints or objectives and enabling partial control over morphological attributes like porosity, anisotropy, and connectivity.

### Effectiveness and Applicability

One of the ultimate objectives of stochastic reconstruction is to facilitate image-based modeling for investigating fundamental microstructure-property relationships. The reconstructed microstructures must preserve key morphological attributes and multiscale fidelity, such as pore connectivity, tortuosity, and percolation characteristics, to ensure reliable simulations of fluid flow, electrical conductivity, heat transfer or mechanical deformation. Besides, a robust reconstruction approach should be adaptable across different material systems, length scales, and engineering applications. However, many existing methods focus on achieving mathematical or visual similarity to real microstructures, often overlooking their usability in practical simulations. To enhance applicability, future research should emphasize physics-aware reconstruction strategies that integrate domain knowledge, ensure compatibility with numerical solvers, and provide reconstructions that are not just statistically equivalent but also functionally reliable for predictive modeling in engineering and scientific applications.

## Future Perspectives and Concluding Remarks

### Future Perspectives

**Fig. 56:**
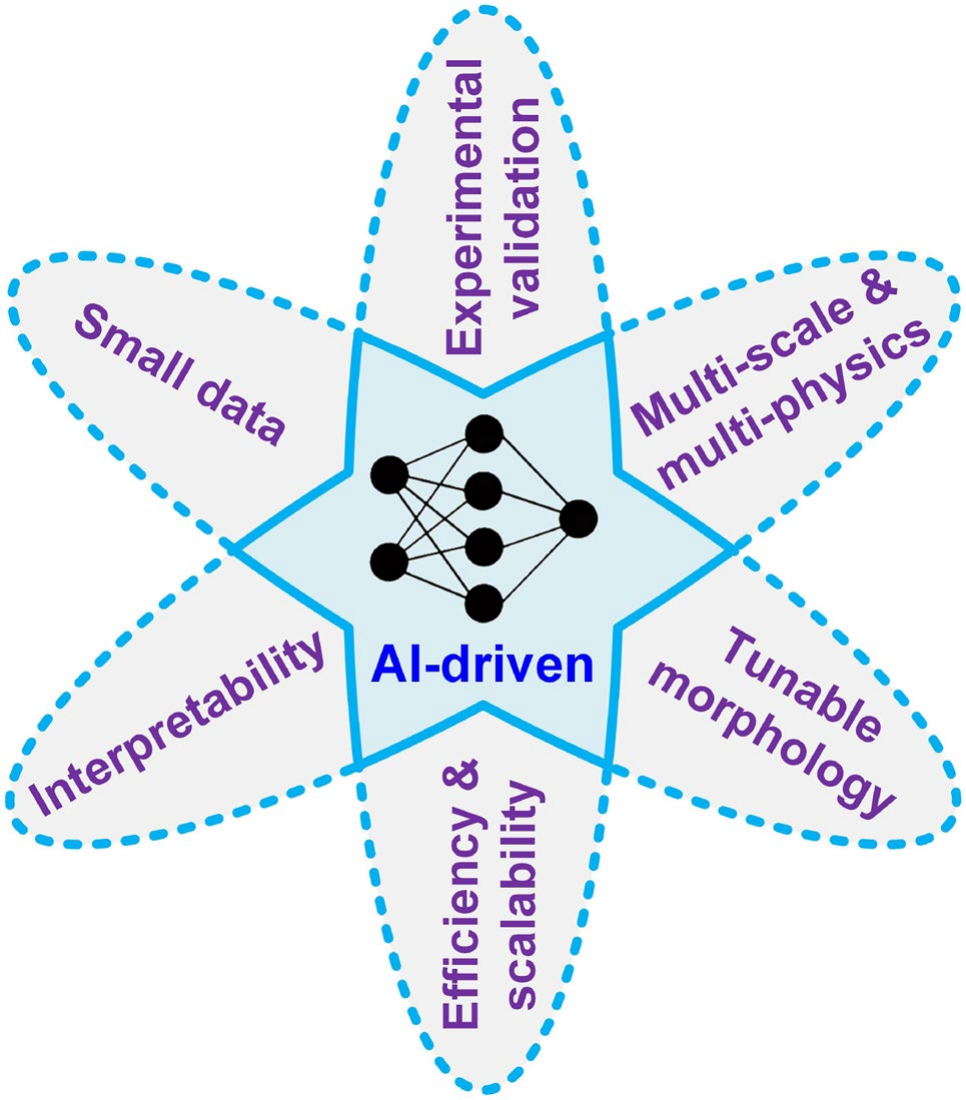
Future perspectives on computational intelligence-based microstructure reconstruction

Despite the remarkable progress in stochastic characterization and microstructure reconstruction using computational intelligence, critical challenges and opportunities remain. This section presents future perspectives on computational intelligence-based microstructure reconstruction, as summarized in Fig 56.

#### Towards Data-Efficient Reconstruction

Developing data-efficient reconstruction methods is essential for advancing machine/deep learning-based microstructure generation. A major challenge in this field is the dependence on large and high-quality training datasets, which are often difficult and costly to obtain using high-resolution 3D imaging techniques such as micro-CT and FIB-SEM. To address this limitation, future efforts should focus on optimizing learning frameworks that require minimal labeled data while maintaining microstructure reconstruction accuracy. Approaches such as transfer learning, self-supervised learning, and few-shot learning can leverage prior knowledge from related porous materials, reducing the need for extensive datasets. Additionally, data augmentation strategies, including physics-aware noise injection, generative adversarial data synthesis, and domain adaptation, can further enhance model robustness in small-data scenarios.

#### Enhancing Interpretability and Explainability

Most machine/deep learning-based reconstruction methods function as black-box models, making it difficult to interpret how specific microstructural features are learned and represented. This lack of transparency complicates the verification of whether reconstructed microstructures faithfully preserve high-order statistical dependencies, topological characteristics, and functional properties. To address this, future research should integrate explainable AI techniques, such as attention mechanisms, feature attribution methods, and visualization tools, to provide deeper insights into the model’s decision-making process. Moreover, incorporating physics-based constraints and domain knowledge into the training process can enhance explainability while maintaining critical morphological and functional attributes. Physics-aware machine learning, can enforce fundamental physical laws during training, ensuring that generated microstructure samples remain physically realistic.

#### Improving Computational Efficiency and Scalability

High-resolution reconstruction of 3D microstructures remains computationally expensive, especially for domains requiring voxel sizes exceeding $1024^3$. The exponential increase in computational cost with sample size limits the feasibility of reconstructing large domains, essential for capturing RVE properties. To address this, future work should focus on improving computational efficiency by leveraging model compression techniques, sparse representations, and hardware acceleration (e.g., tensor processing units, TPUs). Additionally, meta-learning strategies can be employed to train models that rapidly adapt to new microstructure samples without retraining from scratch. Scalable cloud-based solutions and parallelized deep learning architectures can further enhance the accessibility of high-resolution microstructure reconstruction.

#### Tunable Microstructure Generation

Future microstructure reconstruction methods should prioritize tunable generation, enabling precise control over key properties such as volume fraction, specific surface area, tortuosity, and anisotropy. While existing approaches focus on producing statistically equivalent and morphologically similar microstructure samples, they often lack the flexibility to tailor reconstructions to specific functional requirements. Conditional generative models, including CGANs and InfoGANs, offer promising solutions by allowing users to impose constraints and guide the synthesis process toward desired characteristics. Additionally, inverse design techniques that leverage deep generative models can facilitate the on-demand fabrication of optimized microstructures for advanced manufacturing applications.

#### Multi-Scale and Multi-Physics Reconstruction

Random porous media possess hierarchical architectures spanning multiple length scales, from nanometer-scale pore throats to millimeter-scale grain boundaries. However, most current machine/deep learning models struggle to effectively capture this multi-scale heterogeneity. Future advancements should focus on hierarchical generative models, such as multi-scale GANs, variational autoencoders (VAEs), and graph neural networks (GNNs), that can explicitly model structural dependencies across scales. Furthermore, extending stochastic reconstruction beyond morphology to multi-physics properties (such as coupled fluid-solid interactions, thermo-mechanical behavior, and electrochemical transport), will enable more holistic modeling of porous materials. This can be achieved by integrating physics-based simulations into the machine/deep learning pipeline, where microstructure generation is dynamically linked to performance predictions.

#### Bridging the Computation-Experiment Gap

A critical aspect of validating stochastic reconstruction methods is ensuring that the generated microstructure samples accurately reflect real material properties. Experimental validation, including direct comparisons with micro-CT and FIB-SEM images, is necessary to confirm that the reconstructed microstructures align with the real samples [334]. Future research should establish standardized benchmarks for evaluating reconstruction accuracy, integrating metrics such as geometric similarity, percolation thresholds, transport properties, and mechanical response validation. In addition, incorporating experimental feedback into the learning loop can improve reconstruction fidelity and bridge the gap between computational and experimental domains.

### Concluding Remarks

Stochastic microstructure reconstruction is a crucial component of understanding the MPRs and predicting the physical behaviors of random porous media across various applications, including computational materials science, energy storage, geomechanics, and filtration technologies. The advent of computational intelligence has enabled a paradigm shift, allowing for the data-driven generation of complex microstructures that were previously challenging to model using traditional algorithms. Despite the significant advancements, key challenges persist in terms of data efficiency, model interpretability, hierarchical characterization, and computational feasibility. To address these challenges, future research should move towards hybrid methodologies that combine deep learning with physics-based modeling, ensuring that the reconstructed microstructure samples not only visually resemble real materials but also faithfully replicate their functional properties. Enhancing explainability and interpretability should be prioritized to transform machine/deep learning models from black-box solutions into insightful tools for microstructural characterization and stochastic synthesis. In addition, improving computational scalability and efficiency will enable high-resolution and large-size reconstructions that capture the full statistical variability of random porous media. Ultimately, the success of computational intelligence-based microstructure reconstruction will be defined by its ability to generate statistically equivalent, morphological realistic, and physically meaningful microstructure samples that can be directly utilized in image-based poro/micro-mechanical simulations, and real-world applications. By integrating domain knowledge, refining model controllability, and improving computational scalability, future research can unlock the full potential of AI-driven microstructure reconstruction, paving the way for more accurate, efficient, and application-ready solutions.

**Table:** 

**Open-access dataset**	**Website URL**
microlib	https://microlib.io/
Digital Porous Media	https://digitalporousmedia.org/
Materials Commons	https://materialscommons.org/uhcsdb/
Imperial Digital Rocks	https://www.imperial.ac.uk/earth-science/research/research-groups/pore-scale-modelling/micro-ct-images-and-networks/
Stuttgart Digital Rocks	https://www2.icp.uni-stuttgart.de/microct/
DREAM.3D	https://dream3d.bluequartz.net

## Data Availability

The website URLs of several open-access microstructure data repositories are provided in the above table. These repositories encompass a wide range of material systems and imaging modalities, and they offer valuable datasets that can be used for the experimentation, training, and validation of newly developed stochastic microstructure reconstruction methods.
